# Quantified convergence of general homodyne measurements with applications to continuous variable quantum computing

**DOI:** 10.1088/1751-8121/aea45c

**Published:** 2026-09-23

**Authors:** Emanuel Knill, Ezad Shojaee, James R van Meter, Akira Kyle, Scott Glancy

**Affiliations:** 1 National Institute of Standards and Technology, Boulder, CO 80305, United States of America; 2 Center for Theory of Quantum Matter, University of Colorado, Boulder, CO 80309, United States of America; 3 Department of Physics, University of Colorado, Boulder, CO 80309, United States of America; 4 IonQCollege Park, MD 20740, United States of America

**Keywords:** quantum optics, homodyne detection, continuous variable quantum computing

## Abstract

In E. Shojaee *et al* (2025 *Phys. Rev. A* **112** 063708) we introduced *broadband pulsed (BBP) homodyne measurements* as a generalization of standard pulsed homodyne quadrature measurements. BBP can take advantage of detectors such as calorimeters that have the potential for high efficiency over a broad spectral range. BBP homodyne retains the advantages of standard pulsed homodyne, enabling measurement of arbitrary quadratures in the limit of large amplitude local oscillators (LO). Here we quantify the convergence of standard and BBP homodyne quadrature measurements to those of the quadrature of interest. We obtain lower bounds on the fidelity of the post-measurement classical-quantum state of outcomes and unmeasured modes, and the fidelity of the states obtained after applying operations conditional on measurement outcomes. The bounds depend on the LO amplitude and the moments of number operators. We demonstrate the practical relevance of these bounds by evaluating them for standard pulsed homodyne used for estimating values of the characteristic function of the Wigner distribution, expectations of moments, for quantum teleportation and for continuous variable error correction with GKP codes.

## 1. Introduction

Homodyne measurements of the complex amplitude of quantum light are indispensable in quantum optics. Homodyne measurements have a fundamentally simple setup. This setup consists of no more than a beamsplitter combining the signal with a local oscillator (LO), and two detectors after the beamsplitter. The two detector outputs are subtracted and rescaled to obtain the measurement outcomes. The detectors are often photodiodes that record intensity as a function of time. When the LO is always on, the time-resolved record of the subtracted intensities gives information about many amplitudes in parallel. These amplitudes are quadratures of modes of light. Alternatively, one can use a pulsed LO, and measure the total number of photons in each arm without time resolution to measure a specific quadrature determined by the reference pulse.

Our work on general homodyne quadrature measurements [1] was motivated by the problem of measuring extremely broadband, octave-spanning modes of light. These are modes of light whose photons are created into a wavepacket that has a multi-octave spectral width. Such photons are present in femto-second optical frequency combs used for metrology [2] and occur in the inertial-frame description of the Unruh effect associated with accelerating detectors in relativistic quantum optics [3]. For octave-spanning modes of light, there are no efficient, sufficiently fast detectors for time-resolved homodyne measurements. At the cost of a significantly more involved experimental setup, one can separate the frequency band into its components and measure each component separately, or one can use broadband pre-amplification followed by a classical measurement of the spectrum [4]. Alternatively, one can simply use a short pulsed LO and measure each arm as before but with efficient broadband detectors that can be slow. One class of such detectors that may achieve the necessary broadband efficiency consists of calorimeters [5, 6]. Because calorimeters measure energy instead of photon number, the pulsed homodyne measurement scheme needs to be generalized. For this purpose, we introduced broadband pulsed (BBP) homodyne in [1] as a generalization of standard homodyne. We showed BBP homodyne retains all the basic properties of standard pulsed homodyne. In particular, as the amplitude of the LO grows large, the moments of the measurement outcomes converge to those of the target quadrature, and the probability distribution of the outcomes converges weakly to the distribution of the ideal quadrature measurement. Standard homodyne is obtained from BBP in the narrow-band limit where all photons have approximately the same energy.

In order to achieve a desired measurement precision in homodyne measurements it is necessary to use a sufficiently high amplitude LO. The fundamental results of homodyne measurement theory guarantee convergence of moments and weak convergence of probability distributions [1, 7]. Standard arguments for convergence of homodyne to quadrature measurements are based on one-mode semiclassical approximations such as in [8], or on convergence heuristics of expressions for the homodyne observables at finite amplitude, for examples see [9–11]. However, it is necessary to quantify this convergence in order to determine a minimum amplitude sufficient to achieve the desired precision. Such quantifications are available for signal states in specific families of states such as Gaussian states, and observables such as low-degree quadrature moments. The standard advice is to ensure that the average number of photons in the LO pulse is much larger than the average number of photons of the signal. This advice needs modification depending on the goal of the measurement. For example, see [9, 10, 12]. Besides convergence of observables, modern applications of homodyne measurements require convergence of the quantum state of the unmeasured mode after these modes have been modified conditional on measurement outcomes. Such modifications are essential in continuous variable (CV) quantum teleportation [13, 14] and CV quantum computation [15]. Both of these situations involve non-Gaussian quantum states, for which general, quantified bounds on convergence are lacking.

Here we develop and implement strategies for obtaining general bounds on convergence for BBP homodyne measurements of quadratures. We use state distances and fidelities to directly compare the states resulting from homodyne measurement at finite LO amplitude with the states that would be obtained with a direct but realistic quadrature measurement, where we model realistic quadrature measurements as an ideal measurement with realistic noise that can be arbitrarily small. In order to compare the states, we take advantage of a representation of the measurements in terms of von Neumann measurement models [16]. These models involve prepared ancilla modes of a quantum apparatus that are unitarily coupled to the modes being measured. The ancilla modes may then be measured with a fixed von Neumann measurement. Because fidelity between states can only increase under identical data processing [17], it suffices to lower bound the fidelity between the states before the final measurement.

We develop fidelity bounds as a function of LO amplitude for two broad scenarios. The first is for measurement, where we require high fidelity for the joint outcomes and outcome-conditional states remaining in unmeasured systems. The bounds for this scenario can be used to quantify the difference between the expectations of quadrature functions such as moments and the corresponding functions of BBP measurement outcomes. The second scenario involves applying quantum operations conditional on measurement outcomes. This scenario applies to CV quantum teleportation and computation.

To demonstrate the relevance of our convergence bounds, we apply them to standard pulsed homodyne. We calculate fidelity bounds as a function of LO amplitude for the classical-quantum state involving measurement outcomes and unmeasured systems, and for measuring values of the characteristic function of the Wigner distribution and expectations of quadrature moments. The bounds for the classical-quantum state and values of the characteristic function are practically useful and are expressed in terms of expectations of number operators. The bound on the difference of expectations of moments depends as expected on high-order moments. The dependence on the LO amplitude is not optimal, but the bound can still serve as a guide. For examples involving conditional actions, we apply our bounds to CV quantum teleportation and CV quantum error correction. These bounds depend on expectations of low-degree products of quadratures and number operators. The bounds for CV quantum error correction can be used to choose the LO amplitude to ensure small added error without unnecessarily large LO powers. We show that the required LO amplitude is comparable to amplitudes used in existing implementations of pulsed homodyne measurements with a pair of matched photodiodes [18, 19].

## 2. Overview

We establish notation and review the principles and formalism of BBP homodyne in section 3. We then introduce the measurement models used to represent direct quadrature measurements and BBP measurements of the quadrature (section 4). The models are based on the von Neuman measurement models involving couplings between the measured system and a quantum apparatus. We use these models to mathematically represent any actual measurement with classical outcomes as a quantum process followed by a positive operator-valued measure (POVM) on the apparatus with classical output. When comparing direct and BBP measurements of a quadrature, we use the same POVM on the apparatus. Because of monotonicity of fidelity under quantum processing [17], the fidelity between the states obtained by the quantum process serves as a lower bound on the final fidelity of the joint state of the unmeasured quantum systems and the classical outcomes. This fidelity serves as a measure of joint closeness of the classical outcome distributions and of the states of the unmeasured quantum systems.

Our results quantifying the fidelity of BBP measurement outcomes and remaining quantum states are in section 5. The main result, theorem 5.1 provides a general bound for the distance between the state obtained by coupling the quantum apparatus to the target quadrature, and the state obtained by coupling the apparatus to the effective BBP observable defined in equation (6). Theorem 5.2 applies the general bound to the specific case where the apparatus is initialized with a pure-state wavefunction according to the von Neumann measurement model. We also determine bounds on the distance between states obtained by applying functions of the target quadrature or the BBP observable, which can be interpreted as a quantified strong convergence for certain bounded continuous functions. For other functions we establish a regularization procedure in proposition 5.4 that makes it possible to replace the function with a better-behaved one.

We analyze fidelities for outcome-conditional actions such as those required for quantum teleportation in section 6. We first consider conditional unitaries acting on the unmeasured systems and defined by operators $\hat{U}(x)$ for each real x. They can either be applied conditional on the target quadrature’s values or conditional on the BBP observable’s values. In experimental settings, measurements such as those defined in section 5 are performed, and $\hat{U}(x)$ is applied if the outcome is x. We split the fidelity bounds into two steps. In the first step, we compare the conditional unitaries applied without measurement directly to the system of the target quadrature and the unmeasured systems. Proposition 6.2 establishes fidelity bounds for the case where the unitaries $\hat{U}(x)$ are commuting displacements. Applying this proposition may require regularization of the operators $\hat{U}(x)$ by applying lemma 6.3. For the second step, proposition 6.4 determines the added loss of fidelity associated with first measuring, then applying the appropriate unitary conditional on the outcome. This proposition is a general prescription that is implemented for conditional displacements in proposition 6.5.

To illustrate the use of these bounds, we apply them to standard pulsed homodyne in section 7. We calculate the bound on the classical-quantum fidelity of realistic homodyne measurements of quadratures. The bounds depend linearly on the total photon number expectation in the modes being measured, as expected from the heuristic guidelines for setting LO amplitudes. The fidelity approaches 1 quadratically in the inverse LO amplitude. The bounds also depend on the type and amount of added noise of the measurement implementation. The less the added noise, the worse is the bound. This effect is expected for fidelity measures that include a comparison of the complete probability distributions. It can be attributed to the impossibility of performing ideal quadrature measurements. For measures based on differences between expectations of observables there is no such effect, as we demonstrate by applying the bounds to measuring the values of the characteristic function of the Wigner distribution. The fidelities again depend linearly on total photon number expectation. The bounds worsen when evaluating the characteristic function at points distant from the origin, requiring high LO amplitude for a given fidelity. Although our bounds are estimated conservatively, the constants are moderate and the bounds can be applied in practice. As another example, we determine conservative bounds on the difference between the expectation of the moments of the target quadrature and the moments of the homodyne observable. In this case, the bounds depend on high-order moments of quadratures. To illustrate the use of our distance bounds for quadrature-conditional unitaries, we apply the bounds to quantum teleportation. For the final example, we estimate the added error from homodyne quadrature measurements in CV quantum error correction. For this example, we consider correction of displacement noise for finite-energy Gottesman–Kitaev–Preskill (GKP) codes. By treating the Gaussian added noise from photodiodes as added displacement noise, it is possible to apply the results for the fidelity of states and measurement outcomes. To illustrate the relevance of the bounds, we evaluate them for the case where the measurement is implemented with a matched pair of photo-diodes, where we use the added noise reported in [18, 19] for specific values. We find that our error bounds are acceptably small for these values of added noise and commonly used LO amplitudes.

For a summary of functional analysis conventions and background required for the results of this paper, see appendix A. We assume without mention that functions that we introduce are measurable, domains of projection-valued measures are standard Borel spaces, and Hilbert spaces are separable. The appendix also contains frequently used identities and inequalities (appendix B), including relationships between fidelity, overlaps and distances.

## 3. BBP homodyne

Standard pulsed and BBP homodyne measurements aim to measure a quadrature of one mode out of many, where the mode measured is determined by the LO pulse shape. Thus we consider finitely many modes defined by mode annihilation operators $\hat{a}_{k}$ for k=1,…,N. The annihilation operators are assumed to define orthogonal modes satisfying $[\hat{a}_{k}^{\dagger},\hat{a}_{l}] = \delta_{k,l}\unicode{x1D7D9}$, where $\unicode{x1D7D9}$ is the identity operator on the modes’ Hilbert space. In general, we consider multiple families of modes consisting of modes $a_{k}, b_{l},c_{m},\ldots$ with associated mode operators $\hat{a}_{k},\hat{b}_{l}, \hat{c}_{m},\ldots$. For simplicity, the mode indices all run from 1 to N, where we fill in extra orthogonal modes as needed to match the numbers of modes. We write $\boldsymbol{a} = (a_{k})_{k = 1}^{N}$ and $\hat{\boldsymbol{a}} = (\hat{a}_{k})_{k = 1}^{N}$, and similarly for other families of modes, where we think of vectors such as $\hat{\boldsymbol{a}}$ as column vectors with operator entries.

The Hilbert space associated with a family of orthogonal modes $\boldsymbol{a}$ is the Fock space, which factors as a tensor product of single-mode Hilbert spaces. The Hilbert space of one mode is that of a quantum harmonic oscillator and spanned by the number states $\ket{0},\ket{1},\ldots$. We label kets, bras and operators with mode labels as needed. For example, the state $\otimes_{k = 1}^{N} \ket{n_{k}}_{a_{k}}$ is the state with exactly $n_{k}$ photons in mode $a_{k}$ for each k.

For identical length vectors $\boldsymbol{x}$ and $\boldsymbol{y}$, the inner product $\boldsymbol{x}\cdot\boldsymbol{y} $ is evaluated as $\sum_{i}x_{i}y_{i}$, where the product is the scalar product or operator product according to the type of the vectors. For complex vectors $\boldsymbol{x}$, $\boldsymbol{x}^{*}$ is the entry-wise complex conjugate. The length of $\boldsymbol{x}$ is $|\boldsymbol{x}| = \sqrt{\boldsymbol{x}^{*}\cdot\boldsymbol{x}}$. The Hadamard product $\boldsymbol{x}*\boldsymbol{y}$ is the vector obtained by multiplying each coordinate separately, that is $(\boldsymbol{x}*\boldsymbol{y})_{k} = x_{k}y_{k}$. The complex zero vector is denoted by $\boldsymbol{0}$. For vectors $\boldsymbol{x}$ whose entries are operators, $\boldsymbol{x}^{\dagger}$ is a vector of the same shape whose entries are the adjoints of entries of $\boldsymbol{x}$.

If $\boldsymbol{\alpha}$ is a vector of complex numbers satisfying $\boldsymbol{\alpha}^{*}\cdot\boldsymbol{\alpha} = 1$, then for the family of modes $\boldsymbol{a}$, the operator $\hat{a}_{\boldsymbol{\alpha}} = \boldsymbol{\alpha}\cdot\hat{\boldsymbol{a}}$ satisfies the properties of a mode annihilation operator. We refer to the mode defined by $\hat{a}_{\boldsymbol{\alpha}}$ as the mode with shape $\boldsymbol{\alpha}$. The quadratures of this mode are of the form

\begin{align*} \hat{x}_{\theta} = -\mathrm i \require{physics}\qty(\mathrm e^{\mathrm i\theta}\hat{a}_{\boldsymbol{\alpha}}^{\dagger}- \mathrm e^{-\mathrm i\theta}\hat{a}_{\boldsymbol{\alpha}}).\end{align*}

Because $\hat{x}_{\theta} = -\mathrm i\qty(\qty(\hat{a}_{\mathrm e^{-\mathrm i\phi}\boldsymbol{\alpha}})^{\dagger}-\hat{a}_{\mathrm e^{-\mathrm i\phi}\boldsymbol{\alpha}})$, instead of specifying a quadrature in terms of a mode and a phase, we directly specify quadratures according to

\begin{align*} \hat{q}_{{\alpha}} = -\mathrm i\qty(\boldsymbol{\alpha}\cdot\hat{\boldsymbol{a}}^{\dagger} - \boldsymbol{\alpha}^{*}\cdot\hat{\boldsymbol{a}}),\end{align*}

where we do not require $\boldsymbol{\alpha}$ to be normalized. The familiy of modes is implicit in the notation for $\hat{q}_{\boldsymbol{\alpha}}$. The commutation relationships are $[\hat{q}_{\boldsymbol{\alpha}},\hat{q}_{\boldsymbol{\beta}}] = \qty(\boldsymbol{\alpha}^{*}\cdot \boldsymbol{\beta} -\boldsymbol{\beta}^{*}\cdot\boldsymbol{\alpha})$. For this and other operators associated with a family of modes, we always make it clear which family of modes they are associated with. The quadrature $\hat{q}_{\boldsymbol{\alpha}}$ is normalized if $|\boldsymbol{\alpha}|^{2} = 1$. This differs from the normalization required for canonical commutation relationships in natural units with ℏ=1. The quadratures satisfy canonical commutation relationships if $|\boldsymbol{\alpha}|^{2} = 1/2$. For example, see [8], equation (13). Thus, the canonical quadrature with respect to vacuum proportional to $\hat{q}_{\boldsymbol{\alpha}}$ is $\hat{q}_{\boldsymbol{\alpha}/(\sqrt{2}|\boldsymbol{\alpha}|)} = \frac{1}{\sqrt{2}}\hat{q}_{\boldsymbol{\alpha}/|\boldsymbol{\alpha}|}$.

For complex vectors $\boldsymbol{\alpha}$, coherent states $\ket{\boldsymbol{\alpha}}$ of a family of modes $\boldsymbol{a}$ satisfy $\hat{a}_{k}\ket{\boldsymbol{\alpha}} = \alpha_{k} \ket{\boldsymbol{\alpha}}$ for all k. The vacuum state of the modes, that is, the state not containing any photons, is the coherent state $\ket{\boldsymbol{0}}$. We fix the phases of coherent states relative to the vacuum state by requiring that $\braket{\boldsymbol{\alpha}}{\boldsymbol{0}}$ is real and positive. For one mode, this is equivalent to requiring that $\ket{\beta}$ have amplitude $\mathrm e^{-|\beta|^{2}/2}$ at $\ket{0}$ when expressed in the number basis. Coherent states are product states with respect to every orthogonal mode basis. In particular $\ket{\boldsymbol{\alpha}} = \otimes_{k = 1}^{N}\ket{\alpha_{k}}_{a_{k}}$.

Every unnormalized quadrature $\hat{q}_{\boldsymbol{\alpha}}$ is associated with a displacement operator

\begin{align*} \hat{D}_{\boldsymbol{\alpha}} = \mathrm e^{\mathrm i\hat{q}_{\boldsymbol{\alpha}}}.\end{align*}

This displacement operator can be thought of as being generated by the Hamiltonian given by the normalized quadrature $\hat{q}_{\boldsymbol{\alpha}/|\boldsymbol{\alpha}|}$ where the evolution time is $|\boldsymbol{\alpha}|$. The displacement operators for one mode satisfy

\begin{align*}\hat{D}_{\beta}\ket{\alpha}&amp; = \mathrm e^{\mathrm i\Im{\alpha^{*}\beta}}\ket{\alpha+\beta},\nonumber\\ \hat{D}_{-\beta} \hat{a} \hat{D}_{\beta} &amp; = \hat{a} + \beta,\nonumber\\ \hat{D}_{-\beta}\hat{q}_{\alpha}\hat{D}_{\beta} &amp; = \hat{q}_{\alpha}+\mathrm i\alpha^{*}\beta -\mathrm i\alpha\beta^{*}, \nonumber\\ \hat{D}_{-\alpha-\beta} \hat{D}_{\beta}\hat{D}_{\alpha}&amp; = \mathrm e^{\mathrm i\Im{\alpha^{*}\beta}}.\end{align*}

**Figure 1.**
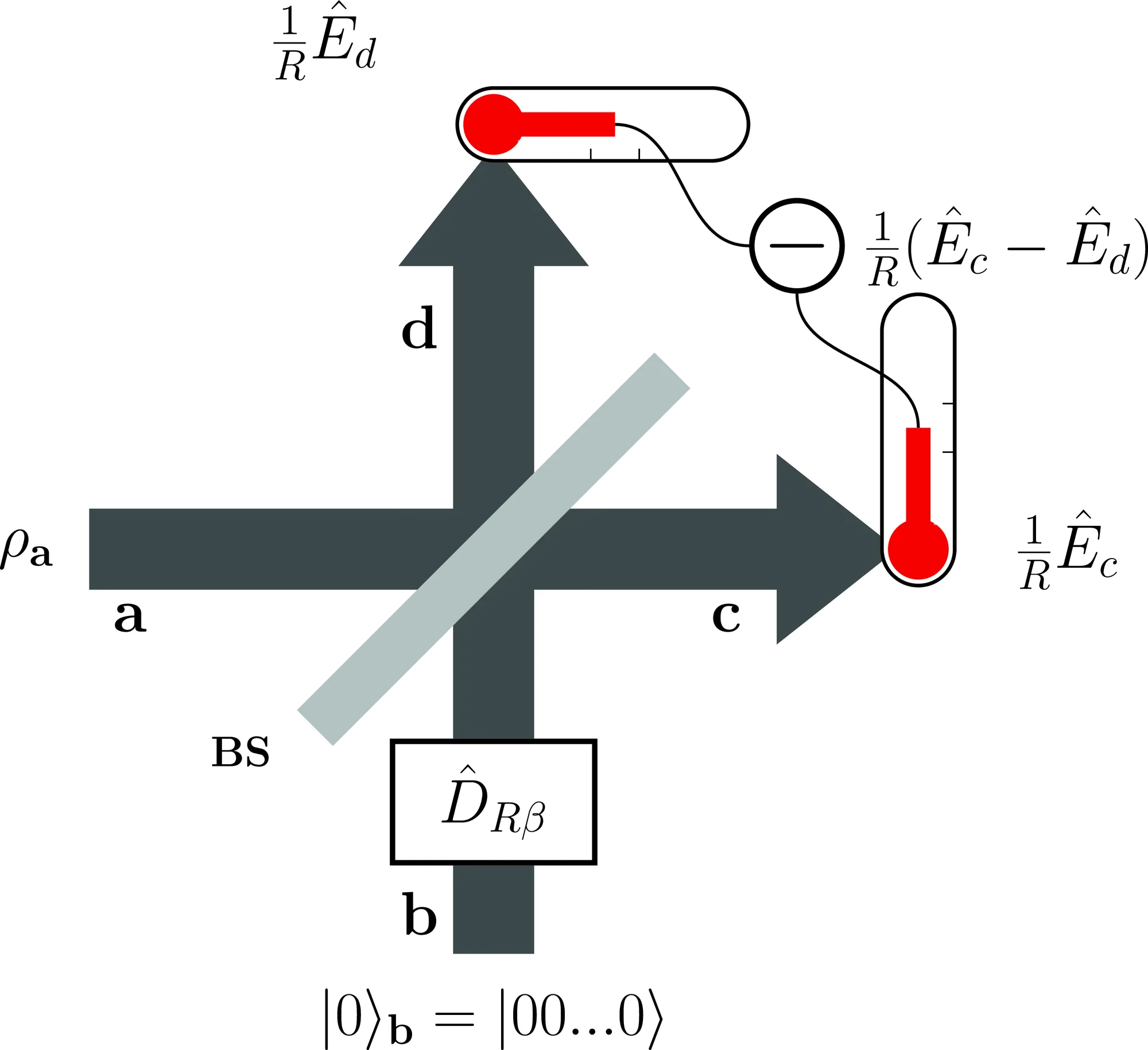
Multi-mode BBP homodyne configuration. The signal modes enter from the left on modes ${\boldsymbol{a}}$. The LO modes enter from the bottom on modes ${\boldsymbol{b}}$. For mathematical convenience, we represent the LO modes as initially in vacuum, with the LO coherent state prepared by the displacement operator $\hat{D}_{R\boldsymbol{\beta}}$. Modes ${\boldsymbol{a}}$ and ${\boldsymbol{b}}$ are combined on a balanced beam splitter (BS). The beamsplitter’s outgoing modes are ${\boldsymbol{c}}$ and ${\boldsymbol{d}}$. They are measured with detectors such as calorimeters. The observables associated with the detectors are of the form $\hat{E}_{\boldsymbol{f}} = \sum_{k}\omega_{k}\hat{f}_{k}^{\dagger} \hat{f}_{k}$ with $\boldsymbol{f} = \boldsymbol{c}$ or $\boldsymbol{d}$, where $\omega_{k}$ are mode energies or other known positive weights. The homodyne measurement result is determined by subtracting one detector’s output from the other and rescaling the result by a factor of 1/R. The corresponding effective measurement operator is shown.

The BBP homodyne configuration is shown in figure 1. The only difference from the standard pulsed homodyne configuration are detectors that measure total energy or another weighted combination of mode number operators instead of total photon number. The measurement operator for modes $\boldsymbol{f}$ is $\hat{E}_{\boldsymbol{f}} = \sum_{k}\omega_{k}\hat{f}_{k}^{\dagger}\hat{f}_{k}$, where $\omega_{k}$ are energies or other weights for mode k. The state to be measured arrives in the signal modes $\boldsymbol{a}$. The LO is prepared in the LO modes $\boldsymbol{b}$. We find it mathematically convenient to make the LO preparation explicit by initializing the LO modes in vacuum, then applying the appropriate displacement $\hat{D}_{R\boldsymbol{\beta}}$, where R is an adjustable positive real scale factor. In practice, the LO pulse is derived from a laser and modified by pulse shaping if necessary. Modes $\boldsymbol{a}$ and $\boldsymbol{b}$ interfere on a balanced beamsplitter. The outgoing modes $\boldsymbol{c}$ and $\boldsymbol{d}$ are measured with the detectors, the measurement outcome of modes $\boldsymbol{d}$ is subtracted from that of modes $\boldsymbol{c}$, and the result of the subtraction is multiplied by 1/R.

The effective BBP measurement operator after subtraction and rescaling can be computed by expressing the outgoing mode operators according to Heisenberg evolution in terms of the incoming modes. With a specific sign and phase convention for the balanced beamsplitter, the Heisenberg forms of the outgoing mode operators are

\begin{align*} \hat{\boldsymbol{c}}^{H} &amp; = \frac{1}{\sqrt{2}}\qty(\hat{\boldsymbol{a}}+\hat{\boldsymbol{b}} + R\boldsymbol{\beta}) ,\nonumber\\ \hat{\boldsymbol{d}}^{H} &amp; = \frac{1}{\sqrt{2}}\qty(\hat{\boldsymbol{a}}-\hat{\boldsymbol{b}} - R\boldsymbol{\beta}).\end{align*}

The Heisenberg form of the BBP measurement operator is therefore

\begin{align*}\frac{1}{R}\Delta \hat{E}^{H} &amp; = \frac{1}{R}\sum_{k = 1}^{N} \omega_{k} \big( {\hat{c}_{k}^{H}}{}^{\dagger} \hat{c}_{k}^{H} - {\hat{d}_{k}^{H}}{}^{\dagger} \hat{d}_{k}^{H} \big) \nonumber\\ &amp; = \sum_{k = 1}^{N} \omega_{k} ( \hat{a}_{k}^{\dagger} \beta_{k} + \beta_{k}^{*} \hat{a}_{k} ) + \frac{1}{R}\sum_{k = 1}^N \omega_{k} ( \hat{a}_{k}^{\dagger} \hat{b}_{k} + \hat{b}_{k}^{\dagger} \hat{a}_{k} ) .\end{align*}

If we choose $\boldsymbol{\beta}$ to be the Hadamard product $\boldsymbol{\beta} = -\mathrm i\boldsymbol{\alpha}*(1/\boldsymbol{\omega})$, then the measurement operator is the unnormalized target quadrature $\hat{q}_{\boldsymbol{\alpha}}$ on the signal modes $\boldsymbol{a}$ plus an operator proportional to 1/R:

\begin{align*} \frac{1}{R}\Delta\hat{E}^{H} = \hat{q}_{{\alpha}} +\frac{1}{R} \qty(\qty(\boldsymbol{\omega}*\hat{\boldsymbol{b}})\cdot\hat{\boldsymbol{a}^{\dagger}} + \qty(\boldsymbol{\omega}*\hat{\boldsymbol{b}}^{\dagger})\cdot\hat{\boldsymbol{a}} ).\end{align*}

For the analysis of convergence of measurements and states to those obtained by measuring the target quadrature directly, we define δ=1/R and write

\begin{align*} \hat{q}_{{\alpha},\delta} = \hat{q}_{{\alpha}} + \delta \qty(\qty(\boldsymbol{\omega}*\hat{\boldsymbol{b}})\cdot\hat{\boldsymbol{a}^{\dagger}} + \qty(\boldsymbol{\omega}*\hat{\boldsymbol{b}}^{\dagger})\cdot\hat{\boldsymbol{a}} )\end{align*}

for the BBP homodyne measurement operator computed for the LO displacement with $\boldsymbol{\beta} = -\mathrm i\boldsymbol{\alpha}*(1/\boldsymbol{\omega})$. Throughout this work, $\hat{q}_{\boldsymbol{\alpha},\delta}$ is expressed as an operator on the incoming mode, which subsumes the displacement and beamsplitter used in the actual measurement. The difference between the BBP homodyne measurement operator and the target quadrature is

\begin{align*} \hat{q}_{{\alpha},\delta} - \hat{q}_{{\alpha}} = \delta \hat{\Delta}_{q},\end{align*}

where we defined the difference operator $\hat{\Delta}_{q} = \qty(\qty(\boldsymbol{\omega}*\hat{\boldsymbol{b}})\cdot\hat{\boldsymbol{a}^{\dagger}} + \qty(\boldsymbol{\omega}*\hat{\boldsymbol{b}}^{\dagger})\cdot\hat{\boldsymbol{a}})$. Because $\hat{q}_{\boldsymbol{\alpha},\delta} - \hat{q}_{\boldsymbol{\alpha}}$ is an unbounded operator for all δ>0, the effect of the difference depends on the incoming state of the signal mode.

The scale of these weights $\omega_{k}$ is arbitrary. Changing the scale also changes the LO amplitude factor R=1/δ. Physical quantities depend on $\delta\omega_{k}$ and are independent of the scale used for the $\omega_{k}$. We therefore normalize the $\omega_{k}$ so that their maximum value is 1. Standard pulsed homodyne corresponds to the case where all $\omega_{k}$ are equal.

## 4. Measurement models

For the remainder of the paper, we fix the target quadrature $\hat{q}_{\boldsymbol{\alpha}}$, where the quadrature is normalized, $|\boldsymbol{\alpha}| = 1$. To simplify the notation we write $\hat{q}_{\delta}$ for the BBP homodyne measurement operator of equation (6) and $\hat{q}$ for the target quadrature, suppressing the suffix $\boldsymbol{\alpha}$. We identify $\hat{q}_{0}$ with $\hat{q}$. Let $\mathrm d\Pi(x)$ and $\mathrm d\Pi_{\delta}(x)$ symbolize the projection-valued measures of the target quadrature $\hat{q}$ and of the BBP measurement operator $\hat{q}_{\delta}$. We identify $\mathrm d\Pi_{0}(x)$ with $\mathrm d\Pi(x)$. See appendix A for a review of the notation and relevant properties for projection-valued measures.

By construction, for δ>0, $\hat{q}_{\delta}$ is the difference of two commuting, displaced and rotated, total energy operators, which implies that it has a discrete spectrum. The displacement is according to the initial displacement of the LO in our representation of the measurement configuration, see figure 1. The corresponding displaced number states are eigenstates of $\hat{q}_{\delta}$. For λ in the spectrum of $\hat{q}_{\delta}$, $\Pi_{\delta}(\lambda)$ can be interpreted as the projector onto the λ-eigenspace of $\hat{q}_{\delta}$. Because the spectrum of $\hat{q}$ is continuous and non-degenerate, $\hat{q}$ has no eigenvectors. The putative projector onto the eigenstate with eigenvalue y, which would be expressed as $\int_{x\in\mathbb{R}} \delta_{y}(x) \mathrm d\Pi(x)$, is 0 because the set $\{x\}$ has measure zero for continuous spectra. See the discussion in appendix A. These considerations indicate that the analysis of convergence of $\hat{q}_{\delta}$ or its measurement to $\hat{q}$ or its measurement, respectively, is not straightforward.

We model measurements as unitary processes that couple the system to be measured to a quantum apparatus, where the apparatus outcomes are defined by a projection-valued measure. After this process, the apparatus may decohere with respect to this measure, and except for such decoherence, the apparatus no longer interacts with other systems. This allows us to learn the outcome of the apparatus and record it elsewhere. To model the usually destructive quadrature measurements, the measured system is discarded. For outcome-dependent actions, the normal experimental order is to record the outcomes, and then take the actions. This is mathematically equivalent to first applying the unitary that conditional on the outcome basis of the apparatus implements the corresponding action, and then decohering the apparatus and recording outcomes if desired. We define the unitary measurement model as the combination of apparatus coupling and, if used, unitarily implemented conditional actions. Conditional actions do not play a role in the remainder of this section, they are defined and analyzed in section 6.

The measurements depend on how the apparatus is coupled to the system. For the unitary measurement model, we use a generalization of the von Neumann measurement model [16]. In the von Neumann measurement model, apparatus couplings are implemented by applying unitaries of the form $\hat{M}_{\delta} = \mathrm e^{-\mathrm i\hat{q}_{\delta}\otimes \hat{s}_{M}}$, where M refers to the apparatus and $\hat{s}_{M}$ is a self-adjoint operator on the apparatus. The initial state of the apparatus is a pure state $\ket{g}_{M}$. Let $\ket{\psi}$ be the initial state of the signal modes $\boldsymbol{a}$, the LO modes $\boldsymbol{b}$, and any additional relevant systems. Then, we obtain the state

\begin{align*} \ket{\phi_{\delta}} = \hat{M}_{\delta}(\ket{\psi}\otimes\ket{g}_{M}).\end{align*}

**Figure 2.**
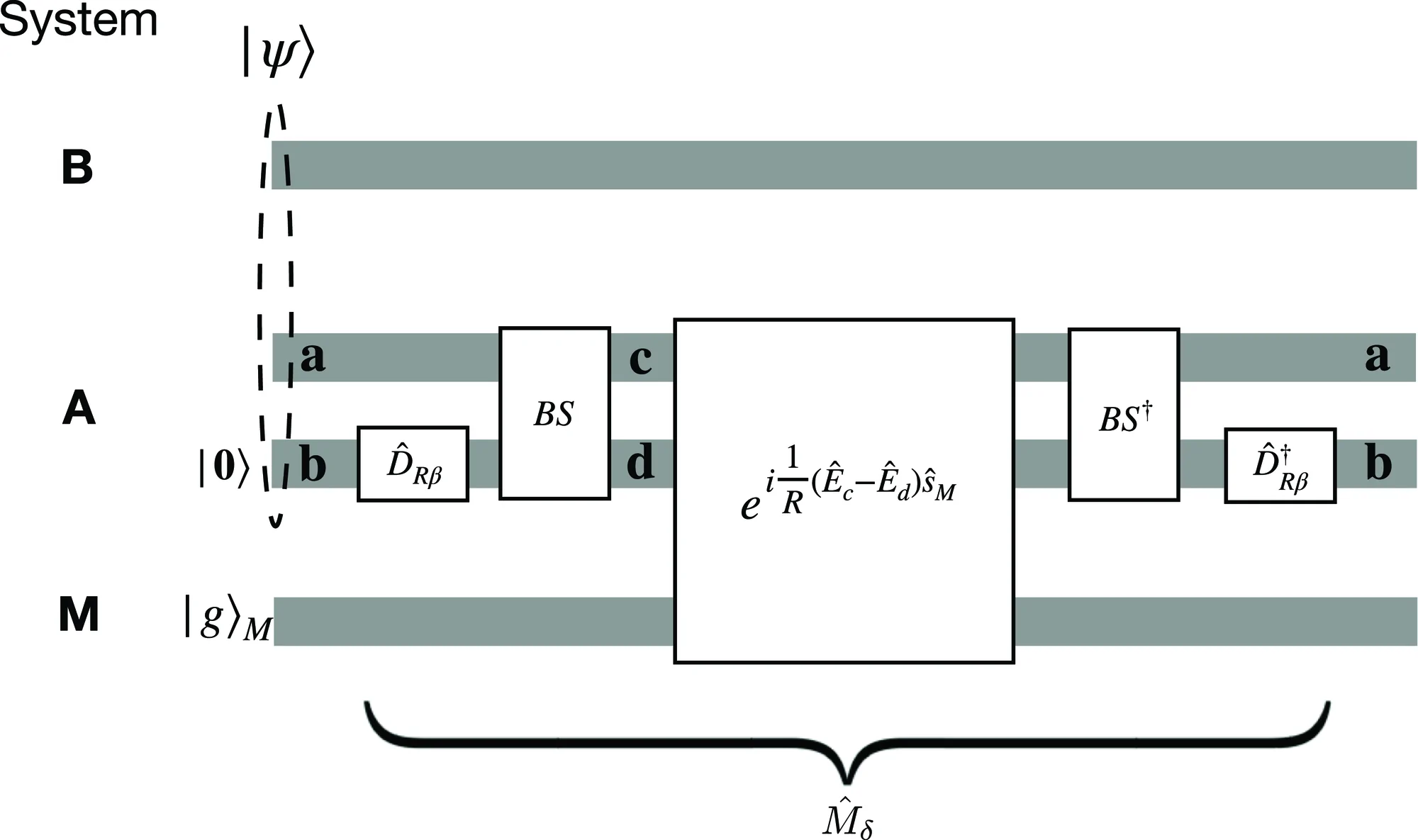
An explicit circuit diagram for the measurement process with effective apparatus coupling $\hat{M}_{\delta} = \mathrm e^{-\mathrm i\hat{q}_{\delta}\otimes \hat{s}_{M}}$. Here we show one way of representing $\hat{M}_{\delta}$ explicitly in terms of the elements depicted in the schematic for BBP homodyne of figure 1. The operator $\hat{E}_{c}-\hat{E}_{d}$ in the diagram denotes the Schrödinger picture operator and is the physical energy difference between the two mode lines at the point where it is used. The coupling to the apparatus in this unitary measurement model is directly in terms of the scaled energy difference operator shown in figure 1, which is converted to $\hat{q}_{\delta}$ as defined in equation (8) by conjugating with the beamsplitter and displacement. The reversal of the beamsplitter and the displacement is used to define $\hat{M}_{\delta}$ in the unitary measurement model. Since they act on subsequently ignored, destroyed or discarded systems they and any other subsequent action on these systems do not matter for the fidelity analysis. In experiments, the actual measurement may be performed as described in figure 1. The system B includes all other relevant systems including any purifying systems.

An explicit circuit diagram for the measurement process is shown in figure 2. The LO modes always start in their vacuum state. The initial state may be assumed to be pure by adding purifying quantum systems if necessary. In general, to quantify convergence we bound the fidelity from below or the Hilbert-space distance from above as a function of δ. For this purpose we review the relevant properties of fidelity.

The fidelity F between pure states $\ket{\phi_{1}}$ and $\ket{\phi_{2}}$ is given by

\begin{align*}F &amp; = |\braket{\phi_{1}}{\phi_{2}}|^{2} = \Re(\braket{\phi_{1}}{\phi_{2}})^{2} + \Im(\braket{\phi_{1}}{\phi_{2}})^{2} \nonumber\\ &amp;{\unicode{x2A7E}} \Re(\braket{\phi_{1}}{\phi_{2}})^{2}.\end{align*}

For useful relationships between fidelities, overlaps and distances, see appendix B. The experimentally relevant fidelities need to account for the apparatus decoherence and outcome recording, which can be treated as a quantum channel and result in mixed states. For mixed states σ and ρ, the fidelity is given by $F(\sigma,\rho) = \qty(\tr(\left|\sqrt{\sigma}\sqrt{\rho}\right|))^{2}$, where for bounded operators $\hat{A}$ and $\hat{B}$, $\tr(\left|\hat{A}\hat{B}\right|) = \tr(\sqrt{\hat{B}^{\dagger}\hat{A}^{\dagger}\hat{A}\hat{B}})$.

We take advantage of monotonicity of fidelity under the application of quantum channels [17] in order to perform our analysis with pure states only. According to monotonicity of fidelity, if $\mathcal{O}$ is a quantum channel, then $F(\mathcal{O}(\sigma),\mathcal{O}(\rho)){\unicode{x2A7E}} F(\sigma,\rho)$. We bound fidelities between the pure states in the unitary measurement model and treat any subsequent decoherence, outcome recording and discarding of quantum systems as quantum channels applied identically to both of the states being compared. In order to motivate and interpret the use of fidelity as a measure of the quality of the measurement process, consider the case where the measurement outcome x is recorded in system O, which may be a computer separate from the quantum apparatus. For illustration we consider the case where the outcomes x are real and, given an initial state ρ, the outcome distribution has probability density $\nu_{\rho}(x)$. The final state of the complete measurement process including eventual readout to a classical system is a quantum–classical state that may be written in the form $\mathcal{O}(\rho) = \int \mathrm{d}x \nu_{\rho}(x)\sigma_{\rho}(x)\otimes {\ketbra{x}{x}}_{O}$, where for $x\in\mathbb{R}$, $\sigma_{\rho}(x)$ is a density operator of the quantum systems remaining after the measurement process has completed. System O contains classical outcomes labeled by x, with outcome states written with quantum notation. For states of this form, the fidelity is given by

\begin{align*} F(\mathcal{O}(\rho_{1}),\mathcal{O}(\rho_{2})) = \qty(\int \mathrm dx \sqrt{\nu_{\rho_{1}}(x)}\sqrt{\nu_{\rho_{2}}(x)}\tr(\left|\sqrt{\sigma_{\rho_{1}}(x)}\sqrt{\sigma_{\rho_{2}}(x)}\right|))^{2}.\end{align*}

This fidelity is a lower bound on the average of the conditional-on-outcomes fidelities between the $\sigma_{\rho_{j}}(x)$, averaged with respect to either $\nu_{\rho_{1}}$ or $\nu_{\rho_{2}}$. To see this, let $\{k,l\} = \{1,2\}$ and use the Cauchy–Schwarz inequality as follows^5^5According to convention, all displayed sequences of inequalities and equalities are to be read as if on one line.:

\begin{align*} F(\mathcal{O}(\rho_{1}),\mathcal{O}(\rho_{2})) &amp;{\unicode{x2A7D}} \left(\int \mathrm dx \nu_{\rho_{k}}(x)\right) \int \mathrm dx\nu_{\rho_{l}}(x)\tr(\left|\sqrt{\sigma_{\rho_{1}}(x)}\sqrt{\sigma_{\rho_{2}}(x)}\right|)^{2} \nonumber\\ &amp; = \int \mathrm dx \nu_{\rho_{l}}(x) F(\sigma_{\rho_{1}}(x),\sigma_{\rho_{2}}(x)),\end{align*}

where we used $\int \mathrm dx \nu_{\rho_{k}}(x) = 1$ since $\nu_{\rho_{k}}(x)$ is a probability density. The average conditional-on-outcome fidelity emphasizes the difference between the states of the quantum systems. The full expression for fidelity also accounts for differences between the observed probability distributions of the outcomes. The latter does not matter if, after taking conditional actions, the outcomes play no further role and are discarded.

To apply the measurement model to BBP homodyne measurements, we elaborate the unitary measurement model. Consider first a BBP measurement that is ideal aside from having δ>0. In this case one would wish for the apparatus outcome basis to consist of $\ket{x}_{M}$ for x real, and for the state after the unitary measurement process to be $\ket{\phi_{\delta}} = \int_{x\in\mathbb{R}} \mathrm d\Pi_{\delta}(x)\ket{\psi}\otimes\ket{x}_{M}$. This makes sense for δ>0 because the spectrum of $\hat{q}_{\delta}$ is discrete. But for δ=0, the state so expressed is not a proper Hilbert-space state. See the discussion in appendix A. If one nevertheless attempts to assess the fidelity between $\ket{\phi_{\delta}}$ and $\ket{\phi_{0}}$ according to the above expression, it appears that the fidelity, to the extent it can be defined, is 0.

The ideal measurements considered in the previous paragraph are not realizable by real-world measurements. For this reason, it is necessary to consider realistic measurements. Realistic measurements do not perfectly resolve the eigenvalues of the operator being measured. In terms of the eventually recorded classical information, this lack of resolution can be described by a stochastic process that, conditional on the true eigenvalue x, produces a measured value z with transition probability density f(z|x). In the unitary measurement model, this effect is achieved with a coupling unitary $\hat{M}_{\delta}$ for which

\begin{align*}\ket{\phi_{\delta}} &amp; = \hat{M}_{\delta}\qty(\ket{\psi}\otimes\ket{g}_M ) \nonumber\\ &amp; = \int_{x\in\mathbb{R}}\mathrm d\Pi_{\delta}(x)\qty(\ket{\psi}\otimes \int_{z\in\mathbb{R}} \mathrm dz f_{1/2}(z|x)\ket{z}_M)\end{align*}

where $f_{1/2}(z|x)$ is a complex-valued function satisfying $|f_{1/2}(z|x)|^{2} = f(z|x)$. Intuitively, after apparatus decoherence, the value recorded in the apparatus behaves as if it had been obtained by the stochastic process above. The coupling unitary can in principle be implemented as a $\hat{q}_{\delta}$-conditional unitary. Translation invariant transition probability densities can be represesented with coupling unitaries $\hat{M}_{\delta}$ of the form given before equation (10). To represent other transition probability densities, we define such unitaries as unitaries that commute with $\hat{q}_{\delta}$ and can be expressed in the form

\begin{align*} \hat{M}_{\delta} = \int_{x\in\mathbb{R}}\mathrm d\Pi_{\delta}(x)\otimes \hat{U}(x)_{M}\end{align*}

with $\hat{U}(x)_{M}$ independent of δ. See appendix A for requirements on $\hat{U}(x)_{M}$ that ensure that $\hat{M}_{\delta}$ is a well-defined unitary operator.

## 5. Quantified convergence of post-measurement states

We first analyze the convergence of the post-measurement state without outcome conditional actions, using the form of the unitary measurement process given in equation (10). The main result is theorem 5.2, which gives a bound on the fidelity between $\ket{\phi_{\delta}}$ and $\ket{\phi_{0}}$ that depends on the coefficients of the target quadrature, the expectation of an energy-related operator on the signal modes, and the second and fourth moment of $\hat{s}_{M}$. The bound on fidelity yields quantified convergence of $f(\hat q_{\delta})\ket{\psi}$ for continuous bounded functions f given that $\ket{\psi}$ has bounded expectation of the energy-related operator. We end the section with a lemma that makes it possible to apply the quantified convergence results to unbounded measurable functions by regularizing these functions. The bounds obtained in this section for BBP homodyne can be improved at the cost of more involved expressions with a more detailed analysis of the inequalities in appendix C. For standard pulsed homodyne the expressions simplify and yield better bounds, as we show in appendix D.

We begin with a fundamental bound that is used both for estimating the fidelity of the measurement process and the fidelity of states obtained after conditional operations. For this bound we consider a general system-apparatus state that is vacuum on the LO modes. We normally express the bounds in terms of distances rather than fidelities, relying on the relationships in appendix B to convert between them.

Theorem 5.1. Let $\ket{\psi}$ be a joint state of the signal, LO modes, the apparatus M and any other relevant systems including those needed for purifying the state. Assume that $\ket{\psi}$ is vacuum on the LO modes. For each mode $a_{k}$, let $\hat{n}_{k}$ be its number operator. Define

\begin{align*} \hat{\Omega}&amp; = \sum_{k}\omega_{k}^{2}\hat{n}_{k}, \nonumber\\ \overline{\omega^{2}} &amp; = \sum_{k}|\alpha_{k}|^{2}\omega_{k}^{2}.\end{align*}

Then

\begin{align*} \left|\mathrm e^{-\mathrm i\hat q_{\delta}s}\ket{\psi} - \mathrm e^{-\mathrm i\hat{q}s}\ket{\psi}\right|^{2} {\unicode{x2A7D}} 4(\delta\,s)^{2}\bra{\psi} \qty(\qty(1+s^{2})\hat{\Omega} + s^{2}\overline{\omega^{2}}) \ket{\psi}.\end{align*}

For every self-adjoint apparatus operator $\hat{s}_{M}$,

\begin{align*} \left|\mathrm e^{-\mathrm i\hat q_{\delta}\hat{s}_{M}}\ket{\psi} - \mathrm e^{-\mathrm i\hat{q}\hat{s}_{M}}\ket{\psi}\right|^{2} {\unicode{x2A7D}} 4\delta^{2}\bra{\psi} \hat{s}_{M}^{2}\qty(\qty(1+\hat{s}_{M}^{2})\hat{\Omega} + \hat{s}_{M}^{2}\overline{\omega^{2}}) \ket{\psi}.\end{align*}

Proof. The inequality of equation (17) is proven in appendix C and is identical to the last inequality of equation (C35). For more detailed and tighter bounds, see the derivation of this inequality. In particular, if the contributions from $s^{2}(\delta\,s)^{2}\hat{\Omega}$ dominate the bound, the bounds may be improvable by applying equation (C37) instead of equation (C35). This comes at the cost of a degree 6 contribution in s. See appendix D for the necessary adjustments for such contributions in standard pulsed homodyne. Up to constants, these tighter bounds cannot be improved as shown in the last part of appendix C by estimates for coherent states. For unitary operators $\hat{U}$ and $\hat{V}$, $|\hat{U}\ket{\psi}+\hat{V}\ket{\psi}|^{2} {\unicode{x2A7D}} 2$, so the bounds in the theorem can be replaced by the minimum of 2 and the bounds shown, if desired.

The inequality of equation (18) follows from equation (17) by expanding the state with respect to the projection-valued measure $\mathrm d\Pi_{M}(s)$ of $\hat{s}_{M}$ according to $\int_{s\in\mathbb{R}}\mathrm d\Pi_{M}(s)\ket{\psi} = \int_{s\in\mathbb{R}}^{\oplus}\mathrm d\mu(s)\ket{\psi(s)}$, where $\mathrm d\mu(s)$ is the measure in the direct-integral representation of $\ket{\psi}$ with respect to $\mathrm d\Pi_{M}(s)$ (appendix A). The conditional states $\ket{\psi(s)}$ satisfy the conditions on $\ket{\psi}$ in the theorem statement and can be substituted for $\ket{\psi}$ in equation (18). Abbreviate $\hat{A} = \mathrm e^{-\mathrm i\hat q_{\delta}\hat{s}_{M}} - \mathrm e^{-\mathrm i\hat{q}\hat{s}_{M}}$, which commutes with $\hat{s}_{M}$.The unnormalized states $\ket{\psi(s)}$ naturally belong to Hilbert spaces of the form $\mathcal{H}_{l}\otimes\mathcal{H}_{R}$, where $\mathcal{H}_{R}$ is the Hilbert space of the systems not including the apparatus and $\mathcal{H}_{l}$ is one of the Hilbert spaces of the direct-integral representation for $\hat{s}_{M}$ on the apparatus Hilbert space. Because $\hat{s}_{M}$ has eigenvalue s on the Hilbert space associated with $s\in\mathbb{R}$ in the direct integral, the operator $\hat{A}$ acts as $\mathrm e^{-\mathrm i\hat{q}_{\delta}s}-\mathrm e^{\mathrm i\hat{q}s}$ on this Hilbert space, and only on the factor $\mathcal{H}_{R}$. Consequently, $\int_{s\in\mathbb{R}}\hat{A}^{\dagger}\hat{A}\,\mathrm d\Pi_{M}(s)\ket{\psi} = \int_{s\in\mathbb{R}}^{\oplus}\mathrm d\mu(s)\hat{A}^{\dagger}\hat{A}\ket{\psi_{s}}$. We have

\begin{align*} \left|\mathrm e^{-\mathrm i\hat q_{\delta}\hat{s}_{M}}\ket{\psi} - \mathrm e^{-\mathrm i\hat{q}\hat{s}_{M}}\ket{\psi}\right|^{2} &amp; = \bra{\psi}\hat{A}^{\dagger}\hat{A}\ket{\psi} \nonumber\\ &amp; = \int_{s\in\mathbb{R}}\bra{\psi}\hat{A}^{\dagger}\hat{A}\mathrm d\Pi_{M}(s)\ket{\psi} \nonumber\\ &amp; = \int_{s\in\mathbb{R}}\mathrm d\mu(s)\bra{\psi_{s}}\hat{A}^{\dagger}\hat{A}\ket{\psi_{s}} \nonumber\\ &amp; = \int_{s\in\mathbb{R}}\mathrm d\mu(s) \left|\mathrm e^{-\mathrm i\hat q_{\delta}\hat{s}_{M}}\ket{\psi_{s}} - \mathrm e^{-\mathrm i\hat{q}\hat{s}_{M}}\ket{\psi_{s}}\right|^{2} \nonumber\\ &amp;{\unicode{x2A7D}} \int_{s\in\mathbb{R}}\mathrm d\mu(s) 4\delta^{2}\bra{\psi_{s}}s^{2} \qty(\qty(1+s^{2})\hat{\Omega} + s^{2}\overline{\omega^{2}}) \ket{\psi_{s}} \nonumber\\ &amp; = \int_{s\in\mathbb{R}} 4\delta^{2}\bra{\psi}s^{2} \qty(\qty(1+s^{2})\hat{\Omega} + s^{2}\overline{\omega^{2}}) \mathrm d\Pi(s)\ket{\psi} \nonumber\\ &amp; = 4\delta^{2}\bra{\psi} \hat{s}_{M}^{2}\qty(\qty(1+\hat{s}_{M}^{2})\hat{\Omega} + \hat{s}_{M}^{2}\overline{\omega^{2}}) \ket{\psi},\end{align*}

which is the right-hand side of the desired inequality. □

The next theorem is a special case of theorem 5.1 and determines the distance between the states after the unitary measurement process, which determines the fidelity between the states. As discussed in section 4, when expressed in terms of fidelity, the bounds are valid for the quantum–classical states obtained after any further processing independent of δ, including decoherence, classical readouts and discarding of systems. For a wavefunction g(s) in $L^{2}(\mathbb{R})$, we write the pure quantum state corresponding to g(s) as $\ket{g(\boldsymbol{\cdot})}$.

Theorem 5.2. Let $\hat{\Omega}$ and $\overline{\omega^{2}}$ be as defined in theorem 5.1. Let $\hat{s}_{M}$ have non-degenerate spectrum so that the apparatus Hilbert space has a representation $L^{2}(\mathbb{R}, \mathrm d\mu(s))$, and let $\ket{g(\boldsymbol{\cdot})}$ be the initial state of the apparatus. Define

\begin{align*} b_{g,2l} = \int_{s\in\mathbb{R}} \mathrm d\mu(s) s^{2l}|g(s)|^{2}.\end{align*}

Let $\ket{\psi}$ be the initial state of the signal, LO modes and systems other than the apparatus, where $\ket{\psi}$ is vacuum on the LO modes. Then, with $\ket{\phi_{\delta}}$ as in equation (10), we have

\begin{align*} \left|\vphantom{f^{2}}\ket{\phi_{\delta}}-\ket{\phi_{0}}\right|^{2} {\unicode{x2A7D}} 4\delta^{2}\qty( b_{g,2}\bra{\psi}\hat{\Omega}\ket{\psi} + b_{g,4}\qty(\bra{\psi}\hat{\Omega}\ket{\psi}+ \overline{\omega^{2}}) ).\end{align*}

Proof. We substitute $\ket{\psi}\otimes \ket{g(\boldsymbol{\cdot})}$ for $\ket{\psi}$ in theorem 5.1. With this substitution, we have

\begin{align*} b_{g,2l} = (\bra{\psi}\otimes\bra{g(\boldsymbol{\cdot})})\hat{s}^{2l} (\ket{\psi}\otimes\ket{g(\boldsymbol{\cdot})}).\end{align*}

The inequality of equation (21) then follows by substitution into the inequality of equation (18). □

The following proposition quantifies the convergence of functions of $\hat{q}_{\delta}$ in a state-dependent way.

Proposition 5.3. Let $\hat{\Omega}$ and $\overline{\omega^{2}}$ be as defined in theorem 5.1. Suppose that f(x) is the Fourier transform of a bounded complex measure $\mathrm d\tilde\mu(\kappa)$. Write $\mathrm d\tilde\mu(\kappa) = \mathrm e^{\mathrm i\theta(\kappa)}\mathrm d\mu(\kappa)$ for a bounded positive measure $\mathrm d\mu(\kappa)$. Define $\tilde{f}_{l} = \int_{\kappa\in\mathbb{R}} \mathrm d\mu(\kappa) |\kappa|^{l}$. Then for all states $\ket{\psi}$ of the signal and LO modes and other relevant systems, where $\ket{\psi}$ is vacuum on the LO modes, we have the following bound:

\begin{align*} \left|\vphantom{f^{2}}f(\hat{q}_{\delta})\ket{\psi}-f(\hat{q}_{0})\ket{\psi}\right|^{2} {\unicode{x2A7D}} 4\delta^{2}(\tilde f_{0}+\tilde f_{2}) \tilde f_{2}\bra{\psi}(\hat{\Omega}+\overline{\omega^{2}})\ket{\psi}.\end{align*}

See appendix A for a brief review of bounded complex measures and our convention for Fourier transforms.

Proof. According to the spectral theorem in projection-valued measure form, for every normalized state $\ket{\phi}$, we have

\begin{align*}\bra{\phi}f(\hat{q}_{\delta})\ket{\psi} &amp; = \int_{x\in\mathbb{R}} \bra{\phi} \mathrm d\Pi_{\delta}(x)\ket{\psi} f(x)\nonumber\\ &amp; = \int_{x\in{\mathbb{R}}} \bra{\phi}\mathrm d\Pi_{\delta}(x)\ket{\psi} \int_{\kappa\in\mathbb{R}} \mathrm d\tilde\mu(\kappa) \mathrm e^{\mathrm ix\kappa}.\end{align*}

The expression $\bra{\phi}\mathrm d\Pi_{\delta}(x)\ket{\psi}$ is a bounded complex measure on $\mathbb{R}$, so we can write $\bra{\phi}\mathrm d\Pi_{\delta}(x)\ket{\psi} = \mathrm e^{\mathrm i\theta^{^{\prime}}(x)}\mathrm d\nu(x)$ with $\mathrm d\nu(x)$ a bounded positive measure. The expression $\mathrm d\nu(x) \mathrm d\tilde\mu(\kappa)$ defines a bounded complex measure on $\mathbb{R}^{2}$. Because the integrands and measures are bounded, we can apply the Fubini-Tonelli theorem to write the last expression in equation (24) as

\begin{align*} \int_{x\in\mathbb{R}} \bra{\phi}\mathrm d\Pi_{\delta}(x)\ket{\psi} \int_{\kappa\in\mathbb{R}} \mathrm d\tilde\mu(\kappa) \mathrm e^{\mathrm ix\kappa} &amp; = \int_{x\in\mathbb{R}} \mathrm d\nu(x) \mathrm e^{\mathrm i\theta^{^{\prime}}(x)} \int_{\kappa\in\mathbb{R}} \mathrm d\mu(\kappa)\mathrm e^{\mathrm ix\kappa}\mathrm e^{\mathrm i\theta(\kappa)} \nonumber\\ &amp; = \int_{\kappa\in\mathbb{R}} \mathrm d\mu(\kappa) \mathrm e^{\mathrm i\theta(\kappa)}\int_{x\in\mathbb{R}} \mathrm d\nu(x)\mathrm e^{\mathrm ix\kappa}\mathrm e^{\mathrm i\theta^{^{\prime}}(x)}\nonumber\\ &amp; = \int_{\kappa\in\mathbb{R}} \mathrm d\tilde\mu(\kappa) \int_{x\in\mathbb{R}} \bra{\phi}\mathrm d\Pi_{\delta}(x)\ket{\psi} \mathrm e^{\mathrm ix\kappa}\nonumber\\ &amp; = \int_{\kappa\in\mathbb{R}} \mathrm d\tilde\mu(\kappa)\bra{\phi} \mathrm e^{\mathrm i\kappa\hat{q}_{\delta}}\ket{\psi}.\end{align*}

We can therefore use the Cauchy–Schwarz inequality and apply equation (17) to bound

\begin{align*}\left|\vphantom{f^{2}}\bra{\phi}f(\hat{q}_{\delta}) - f(\hat{q}_{0})\ket{\psi}\right|^{2} &amp; = \left|\int_{\kappa\in\mathbb{R}} \mathrm d\tilde\mu(\kappa) \bra{\phi}\mathrm e^{\mathrm i\kappa\hat{q}_{\delta}}- \mathrm e^{\mathrm i\kappa\hat{q}_{0}}\ket{\psi}\right|^{2} \nonumber\\ &amp; = \left|\int_{\kappa\in\mathbb{R}} \mathrm d\mu(\kappa)\mathrm e^{\mathrm i\theta(\kappa)} \bra{\phi}\mathrm e^{\mathrm i\kappa\hat{q}_{\delta}}- \mathrm e^{\mathrm i\kappa\hat{q}_{0}}\ket{\psi}\right|^{2} \nonumber\\ &amp;{\unicode{x2A7D}} \qty(\int_{\kappa\in\mathbb{R}} \mathrm d\mu(\kappa) \left|\bra{\phi}\qty(\mathrm e^{\mathrm i\kappa\hat{q}_{\delta}}- \mathrm e^{\mathrm i\kappa\hat{q}_{0}})\ket{\psi}\right|)^{2} \nonumber\\ &amp;{\unicode{x2A7D}} \qty(\int_{\kappa\in\mathbb{R}} \mathrm d\mu(\kappa) \left|\qty(\mathrm e^{\mathrm i\kappa\hat{q}_{\delta}}- \mathrm e^{\mathrm i\kappa\hat{q}_{0}})\ket{\psi}\right|)^{2} \nonumber\\ &amp; = \qty(\int_{\kappa\in\mathbb{R}} \mathrm d\mu(\kappa)\sqrt{1+\kappa^{2}}\frac{1}{\sqrt{1+\kappa^{2}}} \left|\qty(\mathrm e^{\mathrm i\kappa\hat{q}_{\delta}}- \mathrm e^{\mathrm i\kappa\hat{q}_{0}})\ket{\psi}\right|)^{2} \nonumber\\ &amp;{\unicode{x2A7D}} \int_{\kappa\in\mathbb{R}}\mathrm d\mu(\kappa)(1+\kappa^{2}) \int_{\kappa\in\mathbb{R}}\mathrm d\mu(\kappa)\frac{1}{1+\kappa^{2}} \left|\qty(\mathrm e^{\mathrm i\kappa\hat{q}_{\delta}}- \mathrm e^{\mathrm i\kappa\hat{q}_{0}})\ket{\psi}\right|^{2} \nonumber\\ &amp;{\unicode{x2A7D}} 4\delta^{2} (\tilde f_{0}+\tilde f_{2}) \int_{\kappa\in\mathbb{R}}\mathrm d\mu(\kappa)\frac{1}{1+\kappa^{2}} \kappa^{2} \qty(\bra{\psi}\qty(1+\kappa^{2})\hat{\Omega} + \kappa^{2}\overline{\omega^{2}}) \ket{\psi} \nonumber\\ &amp;{\unicode{x2A7D}} 4\delta^{2}(\tilde f_{0}+\tilde f_{2}) \int_{\kappa\in\mathbb{R}}\mathrm d\mu(\kappa) \kappa^{2}\bra{\psi}(\hat{\Omega}+\overline{\omega^{2}})\ket{\psi} \nonumber\\ &amp;{\unicode{x2A7D}} 4\delta^{2}(\tilde f_{0}+\tilde f_{2}) \tilde f_{2}\bra{\psi}(\hat{\Omega}+\overline{\omega^{2}})\ket{\psi}.\end{align*}

Here we used $\kappa^{2}/(1+\kappa^{2}) {\unicode{x2A7D}} 1$ to obtain the second to last line. Since this inequality holds for all normalized states $\ket{\phi}$, the inequality of equation (23) follows. □

For a generalization of proposition 5.3, see appendix E. One can use regularization to apply proposition 5.3 to functions that are not the Fourier transform of a bounded complex measure or for which $\tilde f_{0}$ or $\tilde f_{2}$ are too large or infinite. The regularization error can be determined by means of the next proposition. In this proposition, $f_{0}(x)$ is the function of interest, and f(x) is its regularization.

Proposition 5.4. Let $\hat{\Omega}$ and $\overline{\omega^{2}}$ be as defined in theorem 5.1. Let $f_{0}(x), f(x)$ be real-valued functions, and h(x) a non-negative function. Suppose that f(x) is the Fourier transform of a bounded complex measure as in proposition 5.3 and satisfies $|f_{0}(x) - f(x)|{\unicode{x2A7D}} h(x)$. Then for the states $\ket{\psi}$ of proposition 5.3

\begin{align*} |f(\hat{q}_{\delta})\ket{\psi} - f_{0}(\hat{q})\ket{\psi}|^{2} &amp;{\unicode{x2A7D}} \qty(2\delta\sqrt{(\tilde f_{0}+\tilde f_{2}) \tilde f_{2}}\sqrt{\bra{\psi}(\hat{\Omega}+\overline{\omega^{2}})\ket{\psi}} +\sqrt{\bra{\psi} h(\hat{q})^{2}\ket{\psi}})^{2}.\end{align*}

\begin{align*} &amp;{\unicode{x2A7D}} 8\delta^{2}(\tilde f_{0}+\tilde f_{2}) \tilde f_{2}\bra{\psi}(\hat{\Omega}+\overline{\omega^{2}})\ket{\psi} +2\bra{\psi} h(\hat{q})^{2}\ket{\psi}.\end{align*}

Proof. The inequality $|f_{0}(x) - f(x)|{\unicode{x2A7D}} h(x)$ implies that $(f_{0}(\hat{q})-f(\hat{q}))^{2}{\unicode{x2A7D}} h(\hat{q})^{2}$. We compute

\begin{align*} | \qty(f(\hat{q}_{\delta}) - f_{0}(\hat{q}))\ket{\psi}|^{2} &amp;{\unicode{x2A7D}} \qty(| \qty(f(\hat{q}_{\delta})- f(\hat{q}))\ket{\psi}| + | \qty(f(\hat{q}) - f_{0}(\hat{q}))\ket{\psi}|)^{2} \nonumber\\ &amp; = \qty(| \qty(f(\hat{q}_{\delta})- f(\hat{q}))\ket{\psi}| + \sqrt{\bra{\psi}\qty(f(\hat{q}) - f_{0}(\hat{q}))^{2}\ket{\psi}})^{2} \nonumber\\ &amp;{\unicode{x2A7D}} \qty(| \qty(f(\hat{q}_{\delta})- f(\hat{q}))\ket{\psi}| + \sqrt{\bra{\psi}\qty(h(\hat{q}))^{2}\ket{\psi}})^{2} \end{align*}

The first inequality now follows by applying proposition 5.3. For the second inequality apply $(a+b)^{2}{\unicode{x2A7D}} 2(a^{2}+b^{2})$. □

Propositions 5.3 and 5.4 can be used to bound the difference between the expectations of $f(\hat{q}_{\delta})$ and $f(\hat{q})$. By applying equation (B5) we get $|\bra{\psi} f(\hat{q_{\delta}})\ket{\psi}-\bra{\psi}f(\hat{q})\ket{\psi}|^{2} {\unicode{x2A7D}} |(f(\hat{q}_{\delta}) - f(\hat{q}_{0}))\ket{\psi}|^{2}$.

## 6. Quantified convergence for outcome-conditional actions

We next consider unitary operations $\hat{U}_{B}(x)$ applied conditionally to another quantum system B based on measurement outcomes x from a BBP homodyne measurement of $\hat{q}$. To simplify the notation and avoid confusion with tensor product orderings, we omit the tensor product symbol ⊗ and use the system labels $\boldsymbol{a}$, $\boldsymbol{b}$, B and M when needed. We use A for the joint system consisting of modes $\boldsymbol{a}$ and $\boldsymbol{b}$. The ideal conditional operation on the joint system of the signal modes, the LO modes and system B may be expressed as

\begin{align*} \hat{\mathcal{C}}_{0} = \int_{x\in\mathbb{R}} \mathrm d\Pi(x) \hat{U}_{B}(x).\end{align*}

In this section we compare the ideal conditional operation to the operation that applies the unitaries conditional on a BBP measurement outcome modeled as described in section 4. We do this in two steps. In the first step we compare $\hat{\mathcal{C}}_{0}$ to the conditional operation $\hat{\mathcal{C}}_{\delta}$ that applies the unitaries conditional on $\hat{q}_{\delta}$ instead of $\hat{q}$

\begin{align*} \hat{\mathcal{C}}_{\delta} = \int_{x\in\mathbb{R}} \mathrm d\Pi_{\delta}(x) \hat{U}_{B}(x).\end{align*}

In the second step we compare $\hat{\mathcal{C}}_{\delta}$ to the operation that first implements the unitary measurement process $\hat{M}_{\delta}$ for $\hat{q}_{\delta}$, then conditions the unitaries on the apparatus M instead of the signal modes. The conditional unitary after the unitary measurement process is

\begin{align*} \hat{\mathcal{C}}_{M} = \int_{z\in\mathbb{R}}\mathrm d\Pi_{M}(z)\hat{U}_{B}(z),\end{align*}

where $\mathrm d\Pi_{M}(z)$ is the projection valued measure associated with the apparatus outcomes. We assume that the unitary measurement process $\hat{M}_{\delta}$ is of the form given in equation (15) with the action given in equation (14), so that $\hat{M}_{\delta}$ commutes with $\hat{\mathcal{C}}_{\delta}$.

In applications such as quantum teleportation, the measured modes and the measurement outcomes are discarded after the conditional operations have been performed. In any case, decoherence in the outcome basis of the apparatus does not affect the performance of the operation. If decoherence in the control basis of the measured system also does not matter, then we can improve the comparison by applying additional unitaries diagonal in the outcome and control bases to the apparatus and the measured modes after the conditional unitaries. Here we need such additional unitaries only for the case where the conditional unitaries involve non-commuting displacements. The additional unitaries are of the form

\begin{align*} \hat{W}_{AM} = \int_{z\in\mathbb{R},x\in\mathbb{R}} \mathrm d\Pi_{M}(z) \mathrm d\Pi_{\delta}(x) \mathrm e^{\mathrm iw(z,x)},\end{align*}

for a real-valued function w(z,x)

Let $\ket{\psi}$ be the joint initial state of $\boldsymbol{a}$, B and the LO modes $\boldsymbol{b}$, where $\ket{\psi}$ is vacuum on the LO modes. We define the following states:

\begin{align*} \ket{\psi_{c,\delta}} &amp; = \hat{\mathcal{C}}_{\delta}\ket{\psi}\ket{g}_{M}, \nonumber\\ \ket{\psi_{m,\delta}} &amp; = \hat{M}_{\delta}\hat{\mathcal{C}}_{\delta}\ket{\psi}\ket{g}_{M}, \nonumber\\ \ket{\tilde\psi_{m,\delta}} &amp; = \hat{W}_{AM}\hat{\mathcal{C}}_{M}\hat{M}_{\delta}\ket{\psi}\ket{g}_{M}.\end{align*}

Here, $\ket{\psi_{c,0}}$ is the state obtained after applying the ideal conditional unitary $\hat{\mathcal{C}}_{0}$ without any measurement processes. The state after implementing the conditional unitary conditional on BPP measurement outcomes with the additional unitary is $\ket{\tilde\psi_{m,\delta}}$. The goal is to compare the two, taking into account that decoherence of the measured system and apparatus do not affect the performance. We first bound the distance from $\ket{\psi_{c,\delta}}$ to $\ket{\psi_{c,0}}$ and then the distance from $\ket{\psi_{m,\delta}}$ to $\ket{\tilde\psi_{m,\delta}}$. The distance between $\ket{\psi_{c,\delta}}$ and $\ket{\psi_{c,0}}$ is the same as the distance between $\hat{M}_{\delta}\ket{\psi_{c,\delta}}$ and $\hat{M}_{\delta}\ket{\psi_{c,0}}$. We can use theorem 5.2 to bound the distance between $\hat{M}_{0}\ket{\psi_{c,0}}$ and $\hat{M}_{\delta}\ket{\psi_{c,0}}$. The state $\hat{M}_{0}\ket{\psi_{c,0}}$ is what would have been obtained after the ideal conditional unitary with a subsequent unitary measurement process for $\hat{q}$. The bounds obtained give distance bounds for every step in the chain

\begin{align*} \hat{M}_{0}\ket{\psi_{c,0}} \rightarrow \hat{M}_{\delta}\ket{\psi_{c,0}} \rightarrow \hat{M}_{\delta}\ket{\psi_{c,\delta}} = \ket{\psi_{m,\delta}} \rightarrow \ket{\tilde{\psi}_{m,\delta}}.\end{align*}

**Figure 3.**
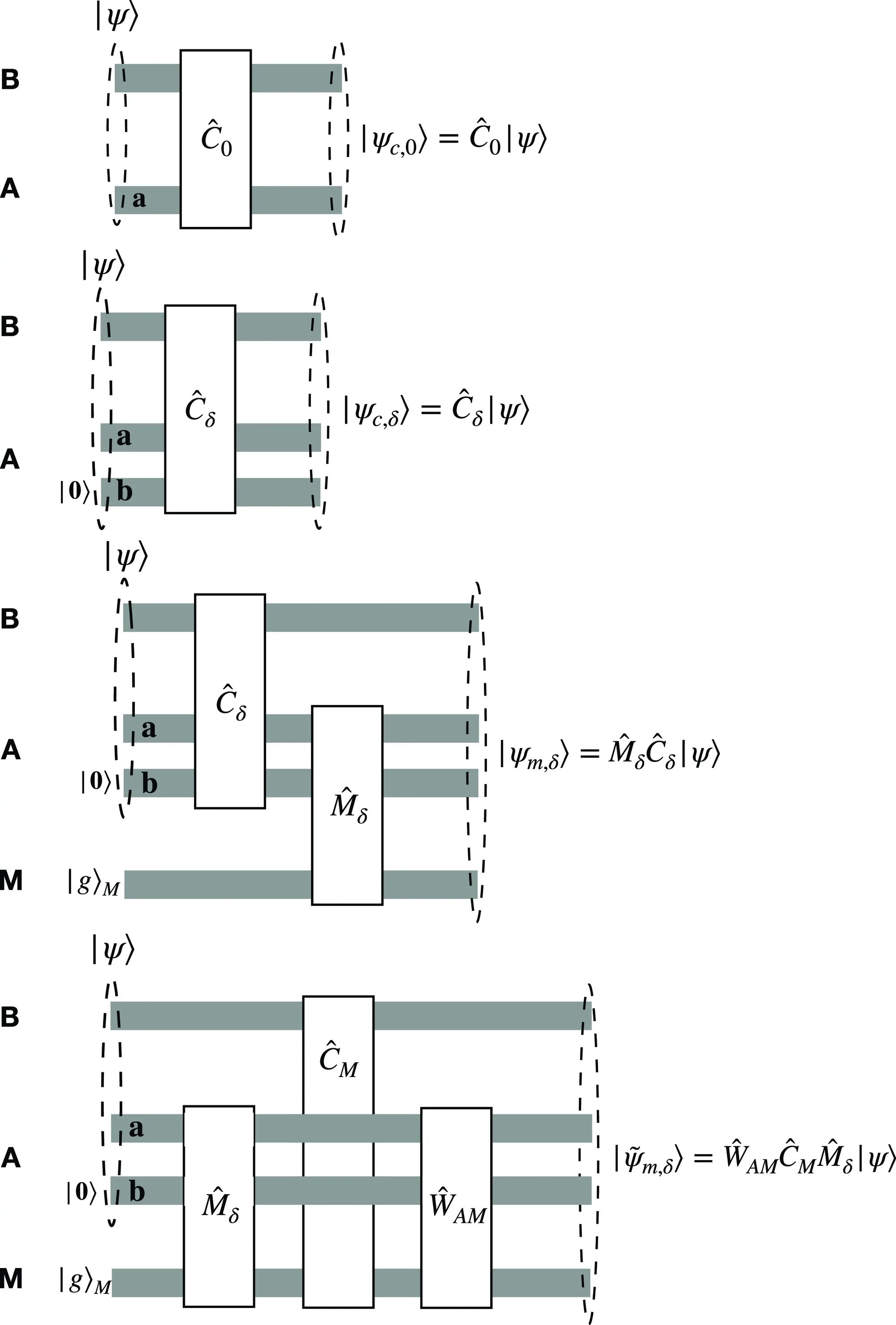
Circuit diagrams for the main states in the distance bounding chain of equation (35).

By adding the bounds along the chain, we obtain a bound on the distance from $\hat{M}_{0}\ket{\psi_{c,0}}$ to $\ket{\tilde{\psi}_{m,\delta}}$, which yields a lower bound on the fidelity between the two states. By monotonicity of fidelity, for implementations of measurement-conditional unitaries with outcome and control decoherence, the overall fidelity is at least as large. Circuit diagrams for the states in each step of the chain are shown in figure 3.

To bound the distance between $\ket{\psi_{c,\delta}}$ to $\ket{\psi_{c,0}}$, we assume that the $\hat{U}_{B}(x)$ mutually commute for different values of x. We first prove a ‘master’ proposition that generalizes proposition 5.3. We then apply this proposition to the case where the conditional unitaries are displacements.

Proposition 6.1 Let $\mathrm d\Pi_{B}(y)$ be a projection-valued measure of system B with $y\in\mathcal{Y}$. Let f(x,y) be a function on $\mathbb{R} \times \mathcal{Y}$ and define

\begin{align*} \hat{f}_{\delta} = \int_{x\in\mathbb{R},y\in\mathcal{Y}}f(x,y)\mathrm d\Pi_{\delta}(x)\mathrm d\Pi_{B}(y) = \int_{y\in\mathcal{Y}}f(\hat{q}_{\delta},y)\mathrm d\Pi_{B}(y).\end{align*}

Suppose that for each y, the function x↦f(x,y) is the Fourier transform of a bounded complex measure $\mathrm d\tilde\nu_{y}(\omega) = \mathrm e^{\mathrm i\phi(\omega)}\mathrm d\nu_{y}(\omega)$. Let $\tilde f_{l}(y)$ be functions on $\mathcal{Y}$ satisfying $\tilde f_{l}(y) {\unicode{x2A7E}} \int |\omega|^{l}\mathrm d\nu_{y}(\omega)$ for all y. Let $\hat{\Omega}$ and $\overline{\omega^{2}}$ be as defined in theorem 5.1. Define the positive operator

\begin{align*} \hat{Q} = \int_{y\in\mathcal{Y}} \mathrm d\Pi_{B}(y)\qty(\tilde f_{0}(y)+\tilde f_{2}(y))\tilde f_{2}(y) \qty(\hat\Omega + \overline{\omega^{2}}).\end{align*}

Then for all states $\ket{\psi}$ of the signal and LO modes, system B and other relevant systems, where $\ket{\psi}$ is vacuum on the LO modes, we have

\begin{align*} \left| \hat{f}_{\delta}\ket{\psi}-\hat{f}_{0}\ket{\psi}\right|^{2} {\unicode{x2A7D}} 4\delta^{2}\bra{\psi}\hat{Q}\ket{\psi}.\end{align*}

Proof. We have

\begin{align*} \hat{f}_{\delta}\ket{\psi}-\hat{f}_{0}\ket{\psi} = \int \mathrm d\Pi_{B}(y)\qty(f(\hat{q}_{\delta},y) - f(\hat{q},y))\ket{\psi}.\end{align*}

Let $\mathrm d\mu(y)$ be the probability measure defined by $\mathrm d\mu(y) = \bra{\psi}\mathrm d\Pi_{B}(y)\ket{\psi}$. Let $\ket{\psi(y)}$ be a square-integrable family of states with respect to $\mathrm d\mu(y)$ that represents $\ket{\psi}$ in the direct-integral representation for $\mathrm d\Pi_{B}(y)$. The Hilbert space $\mathcal{H}(y)$ of $\ket{\psi(y)}$ factors naturally into the Hilbert space of systems other than B and a y-dependent Hilbert space associated with the direct integral representation of $\mathrm d\Pi_{B}(y)$ on B. With this factorization, in the direct integral representation, $\hat{f}_{\delta}$ acts as $f(\hat{q}_{\delta},y)$ on $\ket{\psi(y)}$. We apply proposition 5.3 for each $y\in\mathcal{Y}$ to get

\begin{align*}\left|\hat{f}_{\delta}\ket{\psi}-\hat{f}_{0}\ket{\psi}\right|^{2} &amp; = \int_{y\in\mathcal{Y}} \bra{\psi}\mathrm d\Pi_{B}(y)(f(\hat{q}_{\delta},y)-f(\hat{q},y))^{\dagger} (f(\hat{q}_{\delta},y)-f(\hat{q},y))\ket{\psi} \nonumber\\ &amp; = \int_{y\in\mathcal{Y}} \mathrm d\mu(y) \bra{\psi(y)}(f(\hat{q}_{\delta},y)-f(\hat{q},y))^{\dagger} (f(\hat{q}_{\delta},y)-f(\hat{q},y))\ket{\psi(y)} \nonumber\\ &amp; = \int_{y\in\mathcal{Y}} \mathrm d\mu(y)\left|\vphantom{A^{2}}f(\hat{q}_{\delta},y)\ket{\psi(y)}-f(\hat{q},y)\ket{\psi(y)}\right|^{2} \nonumber\\ &amp;{\unicode{x2A7D}} 4\delta^{2}\int_{y\in\mathcal{Y}} \mathrm d\mu(y)\bra{\psi(y)}\qty(\tilde f_{0}(y) + \tilde f_{2}(y))\tilde f_{2}(y)\qty(\hat\Omega + \overline{\omega^{2}})\ket{\psi(y)} \nonumber\\ &amp; = 4\delta^{2} \int_{y\in\mathcal{Y}}\bra{\psi}\mathrm d\Pi_B(y) \qty(\tilde f_{0}(y) + \tilde f_{2}(y))\tilde f_{2}(y)\qty(\hat\Omega + \overline{\omega^{2}})\ket{\psi} \nonumber\\ &amp; = 4\delta^{2}\bra{\psi}\hat{Q}\ket{\psi}.\end{align*}

 □

The following proposition provides one way to apply proposition 6.1 to the case of conditional displacements. The proposition is formulated for an arbitrary self-adjoint operator on system B. We use the notation $f^{(l)}(x)$ for the l’th derivative of f(x).

Proposition 6.2. Let $\hat{p}_{B}$ be a self-adjoint operator of B, and consider conditional unitaries given by $\hat{U}_{B}(x) = \mathrm e^{\mathrm iv(x)\hat{p}_{B}}$ for a real-valued function v(x). Let $\xi\in\mathbb{R}$ and define w(x)=v(x)−ξx. Suppose that w(x) is twice differentiable and the Fourier transform of the integrable function $\tilde w(\kappa)$. In addition, assume that $w_{l} = \int \mathrm dx |w^{(l)}(x)|$ and $\tilde w_{l} = \int \mathrm d\kappa |\kappa|^{l}|\tilde w(\kappa)|$ are finite for l=0,1,2. Define

\begin{align*} \hat{P}_{B} &amp; = \qty(\frac{1}{\sqrt{\pi}}\qty(\sqrt{w_{0}\tilde w_{0}} +|\hat{p}_{B}|\sqrt{w_{1}\tilde w_{1}})+1), \nonumber\\ \hat{Q}_{B} &amp; = |\hat{p}_{B}|\qty(\tilde w_{2} + |\hat{p}_{B}| \qty(\tilde w_{1}^{2} + 2\xi^{2})).\end{align*}

Then with $\ket{\psi_{c,\delta}}$ as defined in equation (34),

\begin{align*} \left| \ket{\psi_{c,\delta}}-\ket{\psi_{c,0}}\right|^{2} {\unicode{x2A7D}} 4\delta^{2} \bra{\psi} \qty(1+\hat{Q}_{B})\hat{Q}_{B} \hat{P}_{B}^{2}\qty(\hat{\Omega}+\overline{\omega^{2}}) \ket{\psi}.\end{align*}

Proof. The constraints on w(x) and $\tilde w(\kappa)$ are in terms of $L^{1}$ norms of derivatives of w(x) and their Fourier transforms. The $L^{1}$ norms imply bounds on the sup-norm according to

\begin{align*} \|w^{(l)}(x)\|_{\infty} {\unicode{x2A7D}} \tilde w_{l}.\end{align*}

These bounds imply the following bounds on the $L^{2}$-norms of $w^{(l)}(x)$ and $\kappa^{l}\tilde w(\kappa)$:

\begin{align*} 2\pi\|\kappa^{l}\tilde w(\kappa)\|_{2}^{2} &amp; = \|w^{(l)}(x)\|_{2}^{2} \nonumber\\ &amp; = \int_{x\in\mathbb{R}}\mathrm dx |w^{(l)}(x)|^{2} \nonumber\\ &amp;{\unicode{x2A7D}} \|w^{(l)}(x)\|_{\infty}\int_{x}\mathrm dx |w^{(l)}(x)| \nonumber\\ &amp;{\unicode{x2A7D}} \tilde w_{l} w_{l}.\end{align*}

The factor of 2π compensates for our use of non-unitary Fourier transforms.

Let $\mathrm d\Pi_{B}(y)$ be the projection-valued measure of $\hat{p}_{B}$, so that $\mathrm d\Pi_{B}(y)\hat{U}_{B}(x) = \mathrm e^{\mathrm iv(x)y}\mathrm d\Pi_{B}(y)$. If we set $f(x,y) = \mathrm e^{\mathrm iv(x)y}$ in proposition 6.1, then $\hat{f}_{\delta} = \hat{C}_{\delta}$. Let $k_{y}(x) = f(x,y)$. To apply the proposition, for each y we need $k_{y}(x)$ to be the Fourier transform of a bounded complex measure $\mathrm d\tilde\nu_{y}(\kappa) = \mathrm e^{\mathrm i\theta(\kappa)}\mathrm d\nu_{y}(\kappa)$, and it is necessary to determine bounds on $\tilde f_{l}(y) = \int_{\kappa\in\mathbb{R}}\mathrm d\nu_{y}(\kappa)|\kappa^{l}|$ for l=0,2.

We have that $k_{y}(x)$ is the Fourier transform of $\mathrm d\tilde\nu_{y}(\kappa)$ iff $g_{y}(x) = k_{y}(x)\mathrm e^{-\mathrm i\xi xy} = \mathrm e^{\mathrm iw(x)y}$ is the Fourier transform of the displaced measure $\mathrm d\tilde\nu_{y}(\kappa +\xi y)$. The needed bounds can be determined from those for the displaced measure. We have

\begin{align*}\int_{\kappa\in\mathbb{R}} \mathrm d\nu_{y}(\kappa) &amp; = \int_{\kappa\in\mathbb{R}}\mathrm d\nu_{y}(\kappa + \xi y), \nonumber\\ \int_{\kappa\in\mathbb{R}} \mathrm d\nu_{y}(\kappa)|\kappa|^{2} &amp; = \int_{\kappa\in\mathbb{R}} \mathrm d\nu_{y}(\kappa+\xi y) |\kappa+\xi y|^{2} \nonumber\\ &amp;{\unicode{x2A7D}} \int_{\kappa\in\mathbb{R}} \mathrm d\nu_{y}(\kappa+\xi y) 2\qty(|\kappa|^{2}+|\xi y|^{2}) \nonumber\\ &amp; = 2\int_{\kappa\in\mathbb{R}} \mathrm d\nu_{y}(\kappa+\xi y) |\kappa|^{2} + 2|\xi y|^{2}\int_{\kappa\in\mathbb{R}} \mathrm d\nu_{y}(\kappa).\end{align*}

With these inequalities, we can obtain bounds for $k_{y}(x)$ from bounds for $g_{y}(x)$.

Define $h_{y}(x) = g_{y}(x)-1$. Then $g_{y}(x)$ is the Fourier transform of a bounded complex measure iff $h_{y}(x)$ is such a Fourier transform, and the two measures differ by the point measure $\delta(\kappa)$ at 0. We first show that $h_{y}(x)$ is the Fourier transform of a function $\tilde h_{y}(\kappa)$ in $L^{1}(\mathbb{R})$ and obtain a bound on $\|\tilde h_{y}(\kappa)\|_{1}$. We claim that $\tilde h_{y}(\kappa)$ is a well-defined function in $L^{2}(\mathbb{R})$. This follows from

\begin{align*} |h_{y}(x)|^{2} &amp; = 2(1-\Re\qty(\mathrm e^{\mathrm iw(x)y})) \nonumber\\ &amp;{\unicode{x2A7D}} y^{2}w(x)^{2},\end{align*}

so that $\| h_{y}(x)\|_{2} {\unicode{x2A7D}} |y|\sqrt{w_{0}\tilde w_{0}}$. Therefore we can write $h_{y}(x) = \int_{\kappa\in\mathbb{R}} \mathrm d\kappa \mathrm e^{\mathrm i\kappa x}\tilde h_{y}(\kappa)$ for a function $\tilde h_{y}(\kappa)\in L^{2}(\mathbb{R})$. To obtain a bound on this function’s $L^{1}$-norm, we use second differentiability of $h_{y}(x)$, where the derivatives are in $L^{2}(\mathbb{R})$. The derivatives are given by

\begin{align*} h^{(1)}_{y}(x) &amp; =\mathrm iyw^{(1)}(x)\mathrm e^{\mathrm iw(x)y}, \nonumber\\[8pt] h^{(2)}_{y}(x) &amp; = \qty(\mathrm iyw^{(2)}(x)- y^{2}w^{(1)}(x)^{2})\mathrm e^{\mathrm iw(x)y}.\end{align*}

Consequently, the $L^{2}$ norms of the derivatives are $\|h^{(1)}_{y}(x)\|_{2} {\unicode{x2A7D}} |y| \sqrt{w_{1}\tilde w_{1}}$ and $\|h^{(2)}_{y}(x)\|_{2}{\unicode{x2A7D}} |y| \sqrt{w_{2}\tilde w_{2}}+ y^{2}\tilde w_{1}\sqrt{w_{1}\tilde w_{1}}$. For the latter we used $\|w^{(1)}(x)^{2}\|_{2}{\unicode{x2A7D}}\|w^{(1)}(x)\|_{\infty}\|w^{(1)}(x)\|_{2}$. The derivatives $h^{(1)}_{y}(x)$ and $h^{(2)}_{y}(x)$ are the Fourier transforms of the $L^{2}(\mathbb{R})$ functions $\mathrm i\kappa \tilde h_{y}(\kappa)$ and $-\kappa^{2}\tilde h_{y}(\kappa)$. We apply the Cauchy–Schwarz inequality to bound the $L^{1}$ norm of the Fourier transform of $h_{y}(x)$:

\begin{align*} \int_{\kappa\in\mathbb{R}} \mathrm d\kappa |\tilde h_{y}(\kappa)| &amp; = \int_{\kappa\in\mathbb{R}} \mathrm d\kappa \frac{1}{1+|\kappa|} (1+|\kappa|)|\tilde h_{y}(\kappa)| \nonumber\\[8pt] &amp;{\unicode{x2A7D}} \left\|\frac{1}{1+|\kappa|}\right\|_{2} \left\|(1+|\kappa|)|\tilde h_{y}(\kappa)|\right\|_{2} \nonumber\\[8pt] &amp;{\unicode{x2A7D}}\frac{1}{\sqrt{2\pi}}\left\|\frac{1}{1+|\kappa|}\right\|_{2} \qty(\left\|h_{y}(x)\|_{2} + \|h^{(1)}_{y}(x)\right\|_{2}) \nonumber\\[8pt] &amp;{\unicode{x2A7D}} \frac{1}{\sqrt{\pi}}(\sqrt{w_{0}\tilde w_{0}}+|y|\sqrt{w_{1}\tilde w_{1}}),\end{align*}

where we used the identity $\left\|\frac{1}{1+|\kappa|}\right\|_{2} = \int_{-\infty}^{\infty}\mathrm d\kappa/(1+|\kappa|)^{2} = 2\int_{0}^{\infty}\mathrm d\kappa/(1+\kappa)^{2} = -2 /(1+\kappa)|_{0}^{\infty} = 2$.

We also need a bound on the $L^{1}$-norm of the inverse Fourier transform $-\kappa^{2}\tilde h_{y}(\kappa)$ of $h^{(2)}_{y}(x)$. Since $\mathrm e^{\mathrm iw(x)y} = h_{y}(x)+1$, we have

\begin{align*} h^{(2)}_{y}(x) = \qty(\mathrm iyw^{(2)}(x)- y^{2}w^{(1)}(x)^{2}) (h_{y}(x)+1)\end{align*}

We apply the facts that $L^{1}(\mathbb{R})$ with convolution is a Banach algebra, and the Fourier transform of a convolution is the product of the Fourier transforms. Consequently, the $L^{1}$ norm of the inverse Fourier transform of $w^{(1)}(x)^{2}$ is bounded by $\tilde w_{1}^{2}$, from which it follows that the $L^{1}$-norm of the inverse Fourier transform of the factor $\mathrm iyw^{(2)}(x)- y^{2}w^{(1)}(x)^{2}$ on the right-hand side of equation (49) is bounded by $|y|\tilde w_{2}+|y|^{2} \tilde w_{1}^{2}$. Distributing the product over $h_{y}(x)+1$ and applying the Banach property again to the first summand gives

\begin{align*} \int_{\kappa\in\mathbb{R}}\mathrm d\kappa |\kappa|^{2}|\tilde h_{y}(\kappa)| {\unicode{x2A7D}} |y|(\tilde w_{2}+ |y|\tilde w_{1}^{2})\qty( \frac{1}{\sqrt{\pi}}\qty(\sqrt{w_{0}\tilde w_{0}}+|y|\sqrt{w_{1}\tilde w_{1}})+1).\end{align*}

Since $d\tilde \nu_{y}(\kappa+\xi y) = \mathrm d\kappa \tilde h_{y}(\kappa)+\delta(\kappa)$, the inequalities of equation (45) can be continued for

\begin{align*}\int_{\kappa\in\mathbb{R}} \mathrm d\nu_{y}(\kappa) &amp;{\unicode{x2A7D}} \int_{\kappa\in\mathbb{R}}\mathrm d\kappa |\tilde h_{y}(\kappa)| + 1 \nonumber\\ &amp;{\unicode{x2A7D}} \frac{1}{\sqrt{\pi}}\qty(\sqrt{w_{0}\tilde w_{0}}+|y|\sqrt{w_{1}\tilde w_{1}})+1 ,\nonumber\\ \int_{\kappa\in\mathbb{R}} \mathrm d\nu_{y}(\kappa)|\kappa|^{2} &amp;{\unicode{x2A7D}} \int_{\kappa\in\mathbb{R}}\mathrm d\kappa |\kappa|^{2}\tilde h_{y}(\kappa) + 2|\xi y|^{2}\qty(\frac{1}{\sqrt{\pi}}\qty(\sqrt{w_{0}\tilde w_{0}}+|y|\sqrt{w_{1}\tilde w_{1}})+1) \nonumber\\ &amp;{\unicode{x2A7D}} |y|\qty(\frac{1}{\sqrt{\pi}}\qty(\sqrt{w_{0}\tilde w_{0}}+|y|\sqrt{w_{1}\tilde w_{1}})+1) \qty(\tilde w_{2}+ |y|\qty(\tilde w_{1}^{2} + 2\xi^{2})) .\end{align*}

The proposition follows by substitution into the conclusion of proposition 6.1. □

Applying proposition 6.2 requires that v(x) is sufficiently well behaved. This may require regularizing v(x). For this it helps to first compare the intended v(x) to a regularized one by applying the following lemma.

Lemma 6.3 Let $\hat{p}_{B}$ be a self-adjoint operator of B with projection valued measure $\mathrm d\Pi_{B}(x)$, and $\mathrm d\Pi(x)$ be a projection-valued measure on the modes $\boldsymbol{a}$ with $x\in\mathbb{R}$. Let $\hat{x} = \int_{x\in\mathbb{R}}\mathrm d\Pi(x)x$. For real-valued functions u(x) and v(x), consider the two families of unitaries $\hat{U}_{B}(x) = \mathrm e^{\mathrm iu(x)\hat{p}_{B}}$ and $\hat{V}_{B}(x) = \mathrm e^{\mathrm iv(x)\hat{p}_{B}}$ defining conditional unitaries $\mathcal{C}_{\boldsymbol{a}B} = \int_{x\in\mathbb{R}} \mathrm d\Pi(x) \hat{U}_{B}(x)$ and $\mathcal{D}_{\boldsymbol{a}B} = \int_{x\in\mathbb{R}} \mathrm d\Pi(x) \hat{V}_{B}(x)$. Then

\begin{align*} \left|\mathcal{C}_{\boldsymbol{a}B}\ket{\psi}-\mathcal{D}_{\boldsymbol{a}B}\ket{\psi}\right|^{2} {\unicode{x2A7D}} \bra{\psi}\hat{p}_{B}^{2} (u(\hat{x})-v(\hat{x}))^{2}\ket{\psi}.\end{align*}

Proof. It suffices to show that

\begin{align*} \Re\qty(\bra{\psi}\mathcal{D}_{\boldsymbol{a}B}^{\dagger}\mathcal{C}_{\boldsymbol{a}B}\ket{\psi}) {\unicode{x2A7E}} \unicode{x1D7D9}- \frac{1}{2}\bra{\psi}\hat{p}_{B}^{2} (u(\hat{x})-v(\hat{x}))^{2}\ket{\psi}.\end{align*}

We have

\begin{align*} \Re\qty(\mathcal{D}_{\boldsymbol{a}B}^{\dagger}\mathcal{C}_{\boldsymbol{a}B}) &amp; = \int_{x\in\mathbb{R},y\in\mathbb{R}} \mathrm d\Pi(x) \mathrm d\Pi_{B}(y) \Re\qty(\mathrm e^{\mathrm i(u(x)-v(x))y}) \nonumber\\ &amp;{\unicode{x2A7E}} \int_{x\in\mathbb{R},y\in\mathbb{R}} \mathrm d\Pi(x) \mathrm d\Pi_{B}(y) \qty(1- \frac{1}{2}(u(x)-v(x))^{2}y^{2}) \nonumber\\ &amp; = 1-\frac{1}{2}\hat{p}_{B}^{2}(u(\hat{x})-v(\hat{x}))^{2},\end{align*}

where we applied equation (B4) for the second line. The inequality of the lemma follows. □

Next, we bound the distance between $\ket{\psi_{m,\delta}}$ and $\ket{\tilde\psi_{m,\delta}}$. We first give a proposition that provides a general strategy for obtaining lower bounds on $\Re\qty(\braket{\tilde\psi_{m,\delta}}{\psi_{m,\delta}})$. For this proposition, the self-adjoint operator $\hat{q}_{\delta}$ need not be a quadrature of the modes $\boldsymbol{a}$, and the initial state need not be vacuum on the LO modes. More generally, let $\mathrm d\Pi_{A}(x)$ be a projection-valued measure of system A with $x\in\mathcal{B}$. Let $\hat{U}(x)_{B}$ a family of unitary operators defining the conditional unitary $\hat{C}_{AB} = \int_{x\in\mathcal{B}}\mathrm d\Pi_{A}(x)\hat{U}(x)_{B}$. Let the apparatus M have a projection-valued measure $\mathrm d\Pi_{M}(z)$ for $z\in\mathcal{B}$, and let $\hat{M}_{AM}$ be a measurement unitary of the form $\hat{M}_{AM} = \int_{x\in\mathcal{B}}\mathrm d\Pi_{A}(x)\hat{V}(x)_{M}$ for a family of unitary operators $\hat{V}(x)_{M}$ on the apparatus. The initial state of the apparatus M is a fixed pure state $\ket{g}_{M}$. Write $\ket{g_{x}}_{M} = \hat{V}(x)_{M}\ket{g}$. The states $\ket{g_{x}}_{M}$ have a direct-integral representation $\int_{z\in\mathcal{B}}\mathrm d\Pi_{M}(z)\ket{g_{x}} = \int_{z\in\mathcal{B}}^{\oplus}\mathrm d\mu(z)\ket{g_{x}(z)}_{M}$ with respect to a σ-finite measure $\mathrm d\mu(z)$ on $\mathcal{B}$. Then

\begin{align*} \hat{M}_{AM}\ket{\psi}_{AB}\ket{g}_{M} &amp; = \int_{x\in\mathcal{B}}\mathrm d\Pi_{A}(x)\ket{\psi}_{AB}\ket{g_{x}}_{M} \nonumber\\ &amp; = \int_{x\in\mathcal{B},z\in\mathcal{B}}\mathrm d\Pi_{A}(x)\mathrm d\Pi_{M}(z)\ket{\psi}_{AB}\ket{g_{x}}_{M} \nonumber\\ &amp; = \int_{x\in\mathcal{B}}\int_{z\in\mathcal{B}}^{\oplus}\mathrm d\Pi_{A}(x)\mathrm d\mu(z)\ket{\psi}_{AB}\ket{g_{x}(z)}_{M} .\end{align*}

For each x, define the probability distribution $\mathrm d\mu(z)h(z|x) = \mathrm d\mu(z)\braket{g_{x}(z)}$. We define the apparatus conditional unitary $\hat{C}_{MB}$ as $\hat{C}_{MB} = \int_{z\in\mathcal{B}}\mathrm d\Pi_{M}(z) \hat{U}(z)_{B}$. Let $\hat{W}_{AM} = \int_{zx}\mathrm d\Pi_{M}(z)\mathrm d\Pi_{A}(x) \mathrm e^{\mathrm i\omega(z,x)}$ for a real-valued function ω(z,x). Define

\begin{align*} \ket{\psi_{m}} &amp; = \hat{M}_{AM}\hat{C}_{AB}\ket{\psi}_{AB}\ket{g}_{M} \nonumber\\ &amp; = \hat{C}_{AB}\hat{M}_{AM}\ket{\psi}_{AB}\ket{g}_{M}, \nonumber\\ \ket{\tilde\psi_{m}} &amp; = \hat{W}_{AM}\hat{C}_{MB}\hat{M}_{AM}\ket{\psi}_{AB}\ket{g}_{M}.\end{align*}

Proposition 6.4. With the definitions introduced in the previous paragraph, consider a positive semi-definite self-adjoint operator $\hat{Q}_{B}$ and a non-negative function f(z,x) on $\mathcal{B}^{2}$ such that for all z and x

\begin{align*} f(z,x)\hat{Q}_{B} {\unicode{x2A7E}} \Re\qty(1{-}\mathrm e^{-{\mathrm i}w(z,x)}\hat{U}^{\dagger}(z)_{B}\hat{U}(x)_{B}).\end{align*}

Define $ \hat{F}_{A} = \int \mathrm d\Pi_{A}(x) f(z,x)h(z|x)\mathrm d\mu_{M}(z)$. Let $\rho_{A}$ and $\rho_{B}$ be the reduced density matrices on A and B of the state $\ketbra{\psi}{\psi}$. Then

\begin{align*} \left|\ket{\smash{\tilde\psi_{m}}}-\ket{\psi_{m}}\right|^{2} &amp;{\unicode{x2A7D}} 2\bra{\psi}_{AB}\hat{F}_{A}\hat{Q}_{B}\ket{\psi}_{AB}\end{align*}

\begin{align*} &amp;{\unicode{x2A7D}} 2\tr(\rho_{A} \hat{F}_{A}^{2})^{1/2} \tr(\rho_{B} \hat{Q}_{B}^{2})^{1/2}.\end{align*}

Proof. The operator $\hat{Q}_{B}$ need not be bounded. Let $\hat{R}$ be the operator on the right-hand side of the inequality in equation (57). Since $\hat{R}$ is bounded, $\hat{Q}_{B}-\hat{R}$ is selfadjoint with the same domain as $\hat{Q}_{B}$. The inequality is equivalent to the statement that $\hat{Q}_{B}-\hat{R}$ is positive semi-definite.

The expression on the right-hand side of equation (59) is ∞ when $\ket{\psi}$ is not in the domains of $\hat{Q}_{B}$ or $\hat{F}_{A}$, in which case there is nothing to prove. Assume that $\ket{\psi}$ is in the domains of these operators. We have

\begin{align*} \ket{\psi_{m}} &amp; = \hat{\mathcal{C}}_{AB}\int_{x} \mathrm d\Pi_{A}({x}) \ket{\psi}_{AB} \ket{g_{{x}}}_{M} \nonumber\\ &amp; = \int_{x\in\mathcal{B}} \mathrm d\Pi_{A}({x})\hat{U}_{B}({x})\ket{\psi}_{AB}\ket{g_{{x}}}_{M}, \nonumber\\ \ket{\tilde\psi_{m}} &amp; = \hat{W}_{AM} \hat{\mathcal{C}}_{MB}\int_{x\in\mathcal{B}} \mathrm d\Pi_{A}({x}) \ket{\psi}_{AB}\ket{g_{{x}}}_{M} \nonumber\\ &amp; = \int_{z\in\mathcal{B},x\in\mathcal{B}} \mathrm d\Pi_{M}({z})\mathrm d\Pi_{A}({x})\mathrm e^{\mathrm iw({z},{x})} \hat{U}_{B}({z})\ket{\psi}_{AB}\ket{g_{{x}}}_{M} .\end{align*}

Therefore

\begin{align*} \Re\qty(\braket{\tilde\psi_{m}}{\psi_{m}}) &amp; = \int_{x\in\mathcal{B},z\in\mathcal{B}} \bra{\psi}_{AB}\mathrm d\Pi_{A}({x})\mathrm e^{-\mathrm iw({z},{x})} \hat{U}_{B}({z})^{\dagger} \hat{U}_{B}({x})\ket{\psi}_{AB} \bra{g_{{x}}}_{M}\mathrm d\Pi_{M}({z})\ket{g_{{x}}} \nonumber\\ &amp; = \int_{z\in\mathcal{B}, x\in\mathcal{B}}\mathrm d\mu_{M}({z}) \bra{\psi}\mathrm d\Pi_{A}({x})\mathrm e^{-\mathrm iw({z},{x})}\hat{U}_{B}({z})^{\dagger} \hat{U}_{B}({x})\ket{\psi} h(z|x).\end{align*}

From this we get

\begin{align*} 1 - \Re\qty(\bra{\tilde\psi_{m}} \ket{\psi_{m}}) &amp; = \Re\Bigg(\int_{z\in\mathcal{B},x\in\mathcal{B}}\mathrm d\mu(z)\bra{\psi}_{AB} \mathrm d\Pi_{A}({x})\ket{\psi}_{AB} h({z}|{x}) \nonumber\\ &amp;\phantom{{} = \Re\Bigg(} {} - \int_{z\in\mathcal{B},x\in\mathcal{B}} \mathrm d\mu({z})\bra{\psi}_{AB}\mathrm d\Pi_{A}({x})\mathrm e^{-\mathrm iw(z,x)} \hat{U}_{B}({z})^{\dagger} \hat{U}_{B}({x})\ket{\psi}_{AB} h({z}|{x})\Bigg) \nonumber\\ &amp; = \Re\qty(\int_{z\in\mathcal{B},x\in\mathcal{B}} \mathrm d\mu({z})\bra{\psi}_{AB} \mathrm d\Pi_{A}({x}) \qty(1-{\mathrm e}^{-{\mathrm i}w(z,x)} \hat{U}_{B}({z})^{\dagger} \hat{U}_{B}({x}))\ket{\psi}_{AB} h({z}|{x})) \nonumber\\ &amp; = \int_{z\in\mathcal{B},x\in\mathcal{B}}\mathrm d\mu({z}) \bra{\psi}_{AB} \mathrm d\Pi_{A}({x}) \Re\qty(1-{\mathrm e}^{-{\mathrm i}w(z,x)} \hat{U}_{B}({z})^{\dagger} \hat{U}_{B}({x}))\ket{\psi}_{AB} h({z}|{x}) \nonumber\\ &amp;{\unicode{x2A7D}} \int_{z\in\mathcal{B},x\in\mathcal{B}} \mathrm d\mu({z})\bra{\psi}_{AB} \mathrm d\Pi_{A}({x})\hat{Q}_{B}\ket{\psi}_{AB} f({z},{x})h({z}|{x}) \nonumber\\ &amp; = \bra{\psi}_{AB}\hat{Q}_{B}\int_{z\in\mathcal{B},x\in\mathcal{B}} \mathrm d\Pi_{A}({x}) f({z},{x})h({z}|{x})\mathrm d\mu({z})\ket{\psi}_{AB} \nonumber\\ &amp; = \bra{\psi}_{AB}\hat{F}_{A}\hat{Q}_{B}\ket{\psi}_{AB} \nonumber\\ &amp; {\unicode{x2A7D}} \qty(\bra{\psi}_{AB} \hat{F}_{A}^{2} \ket{\psi}_{AB})^{1/2} \qty(\bra{\psi}_{AB} \hat{Q}_{B}^{2} \ket{\psi}_{AB})^{1/2} \nonumber\\ &amp; = \tr(\rho_{A} \hat{F}_{A}^{2})^{1/2} \tr(\rho_{B} \hat{Q}_{B}^{2})^{1/2}.\end{align*}

The last inequality is the Cauchy–Schwarz inequality. The inequalities in the proposition follow by expanding $\left|\ket{\smash{\tilde\psi_{m}}}-\ket{\psi_{m}}\right|^{2}$. □

For the case where the conditional unitaries are displacements, we can obtain explicit expressions for the operators in proposition 6.4. For this purpose, we let $\mathcal{B} = \mathbb{R}^{k}$ and denote members of $\mathcal{B}$ by $\boldsymbol{x}$ and $\boldsymbol{z}$, representing vectors of real values.

Proposition 6.5. In proposition 6.4, let $\hat{U}_{B}(\boldsymbol{x}) = D_{\boldsymbol{\gamma}({\boldsymbol{x}})}$ be displacements acting on modes of B. Then we can choose $w(\boldsymbol{z},\boldsymbol{x})$ such that the conclusions of proposition 6.4 hold with

\begin{align*} f(\boldsymbol{z},\boldsymbol{x}) = \frac{1}{2} |\boldsymbol{\gamma}(\boldsymbol{z})-\boldsymbol{\gamma}(\boldsymbol{x})|^{2},\end{align*}

\begin{align*} \hat{Q}_{B} = 4(\hat{n}_{\mathrm{tot}}+1/2),\end{align*}

where $\hat{n}_{\mathrm{tot}}$ is the total photon number operator on the modes involved in the displacements. Suppose that in addition, $\boldsymbol{\gamma}(\boldsymbol{x})$ satisfies that for all $\boldsymbol{x},\boldsymbol{z}$ we have $|\boldsymbol{\gamma}(\boldsymbol{z})-\boldsymbol{\gamma}(\boldsymbol{x})| {\unicode{x2A7D}} K_{1} |\boldsymbol{z}-\boldsymbol{x}|$, and for all $\boldsymbol{x}$, $\int d\boldsymbol{z}|\boldsymbol{x}-\boldsymbol{z}|^{2}h(\boldsymbol{z}|\boldsymbol{x}) {\unicode{x2A7D}} K_{2}$. Then

\begin{align*} \left|\ket{\smash{\tilde\psi_{m}}}-\ket{\psi_{m}}\right|^{2} {\unicode{x2A7D}} 2K_{1}^{2}K_{2} \qty(\bra{\psi}_{AB}(\hat{n}_{\mathrm{tot}}+1/2)^{2}\ket{\psi}_{AB})^{1/2}.\end{align*}

Proof. We have

\begin{align*} \hat{U}_{B}(\boldsymbol{z})^{\dagger}\hat{U}_{B}(\boldsymbol{x}) = \mathrm e^{-\mathrm i \Im (\boldsymbol{\gamma}(\boldsymbol{x})^{\dagger} \boldsymbol{\gamma}({\boldsymbol{z}}))} D_{\boldsymbol{\gamma}(\boldsymbol{x}) - \boldsymbol{\gamma}(\boldsymbol{z})}.\end{align*}

In view of this identity, we let

\begin{align*} w(\boldsymbol{z},\boldsymbol{x}) = -\Im(\boldsymbol{\gamma}(\boldsymbol{x})^{\dagger}\boldsymbol{\gamma}(\boldsymbol{z})).\end{align*}

We determine a bound on displacements. Consider a general displacement operator $D_{\boldsymbol{\beta}}$. This is unitarily equivalent to the displacement $D_{r} = \mathrm e^{\mathrm ir\hat{p}}$ with $\hat{p}$ a normalized quadrature and $r = |\boldsymbol{\beta}|$. For an arbitrary state $\ket{\phi}$, let $\mathrm d\mu(z) = \bra{\phi} \mathrm d\Pi_{p}(z) \ket{\phi}$, where $\mathrm d\Pi_{p}(z)$ is the spectral measure of $\hat{p}$. Then

\begin{align*} \Re(\bra{\phi} D_{r}\ket{\phi}) &amp; = \int \mathrm d\mu(z) \Re\qty(\mathrm e^{\mathrm irz}) \nonumber \\ &amp; {\unicode{x2A7E}} 1 - \int \mathrm d\mu(z) \frac{r^{2} z^{2}}{2} \nonumber \\ &amp; = 1 - \frac{r^{2}}{2}\bra{\phi} \hat{p}^{2}\ket{\phi}.\end{align*}

By arbitrariness of $\ket{\phi}$, it follows that the operator inequality

\begin{align*} \frac{r^{2}}{2}\hat{p}^{2} {\unicode{x2A7E}} \Re(1- D_{r})\end{align*}

holds. Since $\hat{p}^{2} {\unicode{x2A7D}} 4(\hat{n}+1/2){\unicode{x2A7D}} 4(\hat{n}_{\mathrm{tot}}+1/2)$, we can replace $\hat{p}^{2}$ with $4(\hat{n}_{\mathrm{tot}}+1/2)$ in this inequality.

Next, we apply the bound of the previous paragraph to verify equation (57) of proposition 6.4 for $\hat{Q}_{B} = 4(\hat{n}_{\mathrm{tot}}+1/2)$ and $f(\boldsymbol{z},\boldsymbol{x}) = \frac{1}{2}|\boldsymbol{\gamma}(\boldsymbol{z})- \boldsymbol{\gamma}(\boldsymbol{x})|^{2} {\unicode{x2A7D}} \frac{K_{1}^{2}}{2}|\boldsymbol{z}-\boldsymbol{x}|^{2}$.

\begin{align*} \Re\qty(1{-}\mathrm e^{-\mathrm iw(\boldsymbol{z},\boldsymbol{x})}\hat{U}_{B}^{\dagger}(\boldsymbol{z}) \hat{U}_{B}(\boldsymbol{x})) &amp; = \Re\qty(1{-}\mathrm e^{{-}\mathrm iw(\boldsymbol{z},\boldsymbol{x})} D_{-\boldsymbol{\gamma}(\boldsymbol{z})} D_{\boldsymbol{\gamma}(\boldsymbol{x})}) \nonumber \\ &amp; = \Re\qty(1{-} D_{\boldsymbol{\gamma}(\boldsymbol{x})-\boldsymbol{\gamma}(\boldsymbol{z})}) \nonumber \\ &amp; {\unicode{x2A7D}} \frac{1}{2}|\boldsymbol{\gamma}(\boldsymbol{z})-\boldsymbol{\gamma}(\boldsymbol{x})|^{2} 4(\hat{n}_{\mathrm{tot}}+1/2)\nonumber\\ &amp;{\unicode{x2A7D}} 2K_{1}^{2}|\boldsymbol{z}-\boldsymbol{x}|^{2}(\hat{n}_{\mathrm{tot}}+1/2).\end{align*}

The second-last line is the bound needed for the first part of the proposition. The last line is needed for the second part. To finish establishing the second part, we bound $\hat{F}_{\boldsymbol{a}}$ by bounding the term multiplying $\mathrm d\Pi_{\boldsymbol{a}}(\boldsymbol{x})$ in the definition of $\hat{F}_{\boldsymbol{a}}$ in proposition 6.4:

\begin{align*} \int \mathrm d\mu(z) f(\boldsymbol{z},\boldsymbol{x})h(\boldsymbol{z}|\boldsymbol{x}) &amp;{\unicode{x2A7D}} \int \mathrm d\mu(z)\frac{K_{1}^{2}}{2}|\boldsymbol{x}-\boldsymbol{z}|^{2}h(\boldsymbol{z}|\boldsymbol{x}) \nonumber\\ &amp;{\unicode{x2A7D}} \frac{K_{1}^{2}K_{2}}{2}.\end{align*}

consequently, $\hat{F}_{\boldsymbol{a}}^{2}{\unicode{x2A7D}} K_{1}^{4}K_{2}^{2}/4$. Substituting in the inequality of equation (58) gives the bound in the proposition. □

## 7. Applications to standard pulsed homodyne

Our bounds can be applied to standard pulsed homodyne, which corresponds to having $\omega_{k} = \omega$ for k=1,…,N. With our conventions, $\omega = \omega_{1} = 1$. The LO displacement is then $\frac{\boldsymbol{\beta}}{\delta} = -\frac{\mathrm i\boldsymbol{\alpha}}{\delta}$. Because the target quadrature is normalized, the expected number of photons in the LO after the displacement is $1/\delta^{2}$. For the quantities introduced in theorem 5.1, we get $\hat{\Omega} = \hat{n}_{\mathrm{tot}}$ and $\overline{\omega^{2}} = 1$.

A standard implementation of pulsed homodyne uses a matched pair of photodiodes as the detectors. A detailed description of such an implementation with low added noise is given in [18]. The subtracted photodiode measurement has an effective added noise of $\sigma\approx 730$ electrons before rescaling. Another example of such an implementation is given in [19] with an added noise of $\sigma\approx8250$ electrons. Because the added noise involves a large number of electrons, this can to good approximation be modeled by Gaussian added noise in the measurement. In the von Neumann measurement scheme as used in theorem 5.2, this effect is obtained with the apparatus operator $\hat{s}_{M}$ the momentum operator of a one-dimensional system with Hilbert space $L^{2}(\mathbb{R},\mathrm d\mu(s))$, and with initial real positive Gaussian wave function $\tilde{g}(x)$ in position space satisfying

\begin{align*} \tilde g(x)^{2} = \frac{1}{\sqrt{2\pi}\delta\,\sigma}\mathrm e^{-x^{2}/ (2(\delta\,\sigma)^{2})}.\end{align*}

The operator $\hat{s}_{M}$ generates translations of position space so that $\mathrm e^{-\mathrm i t\hat{s}_{M}}$ translates wavefunctions by a distance of t. The final measurement is in the position basis of the apparatus so that the added Gaussian measurement noise has variance $(\delta\,\sigma)^{2}$ for a resolution of $r = \delta\,\sigma$. This accounts for the rescaling of the original homodyne difference operator by δ and the added noise of standard deviation σ after the subtraction of detector outcomes.

For applying theorem 5.2, we need the wavefunction g(s) in momentum space $L^{2}(\mathbb{R},\mathrm d\mu(s))$. This wave function is given by the unitary Fourier transform of $\tilde{g}(x)$, which is

\begin{align*} g(s) = \frac{\sqrt{2\delta\,\sigma}}{(2\pi)^{1/4}} \mathrm e^{- (\delta\,\sigma)^{2}s^{2}}.\end{align*}

The square $|g(s)|^{2}$ is a standard Gaussian with variance $1/(2\delta\,\sigma)^{2} = 1/(2r)^{2}$.

We refer to the measurement model with g(s) satisfying equation (73) as Gaussian added noise at resolution r. The LO pulses used for the measurements in [18] have $10^{6}$ to $3\times 10^{8}$ photons, which corresponds to $\delta\in [10^{-3},\sqrt{3}\times 10^{-4}]$. In [19], the number of photons in the LO pulses is $0.5\times 10^{9}$ to $2\times 10^{9}$.

### 7.1. Fidelity of measurement outcomes and remaining state

For illustration, we first apply the general bounds for BBP homodyne from section 5. We then improve the bounds with the results for standard pulsed homodyne from appendix D. The bounds obtained show that while the general bounds are practically relevant and can be used regardless of the distribution of the $\omega_{k}$ without complications from higher-degree terms, the specific bounds yield significant improvements.

Theorem 5.2 implies a bound on the fidelity between the states obtained after coupling to the apparatus according to the ideal target quadrature compared to coupling according to the pulsed homodyne observable. The bound depends on the apparatus initial state through the quantities $b_{g,2l}$ defined in the theorem. In view of the relationship between distance and fidelity in appendix B, the bound is

\begin{align*} F_{\delta} {\unicode{x2A7E}} 1-4\delta^{2}\bra{\psi}\qty(b_{g,2}\hat{n}_{\mathrm{tot}} + b_{g,4}\qty(\hat{n}_{\mathrm{tot}}+1))\ket{\psi},\end{align*}

where $\ket{\psi}$ is the purified initial state. This is a lower bound on the classical-quantum fidelity of the joint state of the unmeasured quantum modes and the classical measurement outcomes, which is the relevant fidelity if the classical outcome distribution matters. For the homodyne measurement with Gaussian added noise at resolution r, the quantities $b_{g,2}$ and $b_{g,4}$ can be computed to be $b_{{g},2} = 1/(2r)^{2}$ and $b_{{g},4} = 3/(2r)^{4}$. This gives the bound

\begin{align*} F_{\delta} {\unicode{x2A7E}} 1-4\frac{\delta^{2}}{(2r)^{4}} \bra{\psi}\qty(\qty((2r)^{2}+{3})\hat{n}_{\mathrm{tot}} + {3})\ket{\psi}.\end{align*}

High fidelity requires that ${\delta^{2}\langle\hat{n}_{\mathrm{tot}}\rangle}/{r^{4}}$ is small, which corresponds to requiring that the average number of photons in the LO be larger than the average number of photons in the signal by a factor determined by the resolution. This refines and quantifies the conventional recommendation that the average number of photons in the LO be much larger than the average number of photons in the signal modes [9].

If, as in pulsed homodyne measurements with a matched pair of photodiodes, the added noise is approximately constant before rescaling, the resolution improves with increasing LO amplitude according to $r = \delta\,\sigma$. Higher resolution implies that the measurement of $\hat{q}$ becomes sensitive to finer details of the quadrature probability distribution. At fixed high resolution, it is necessary to have correspondingly high LO amplitude for the homodyne measurement to preserve these fine details. But with an LO amplitude dependent resolution of $r = \delta\,\sigma$, this resolution improves too rapidly with LO amplitude for the LO amplitude to compensate. Expressed in terms of δ and σ, the fidelity tradeoff between detector-added noise σ and LO amplitude is expressed with our bounds by

\begin{align*} F_{\delta} {\unicode{x2A7E}} 1-\frac{4}{(2\sigma)^{4}} \bra{\psi}\qty((2\sigma)^{2}\hat{n}_{\mathrm{tot}}+{3\delta^{-2}}(\hat{n}_{\mathrm{tot}} + 1))\ket{\psi}.\end{align*}

In practice, applications require a particular resolution $r_{0}$. At a given LO amplitude 1/δ, if $\delta\,\sigma < r_{0}$, one can artificially add noise to the homodyne measurement outcomes to increase σ to $\sigma^{^{\prime}} = r_{0}/\delta$ and satisfy $\delta\,\sigma = r_{0}$. The lower bound on fidelity in terms of resolution then applies, and increasing the LO amplitude improves the fidelity according to equation (75) with r replaced by $r_{0}$. Consider the values for photodiode added noise and LO average photon numbers from [18] and assume an upper bound for expected photon number of $\langle \hat{n}_{\mathrm{tot}}\rangle = 5.0$. For $\delta = 10^{-4}$ corresponding to an LO expected photon number of $10^{8}$, we get a respectable resolution and fidelity of

\begin{align*} r&amp; = 0.073, \nonumber\\ F_{10^{-4}} &amp; {\unicode{x2A7E}} 0.998.\end{align*}

Next we update the bounds obtained according to the results of appendix D. With theorem D.2, equation (74) becomes

\begin{align*} F_{\delta} {\unicode{x2A7E}} 1-\delta^{2}\bra{\psi}\qty(2 b_{g,2}\hat{n}_{\mathrm{tot}} + \frac{1}{9}\delta^{2}b_{g,6}\hat{q}^{2} + \frac{1}{2} b_{g,4})\ket{\psi}.\end{align*}

We have $b_{g,6} = 15/(2r)^{6}$ and, conservatively, $\bra{\psi}\hat{q}^{2}\ket{\psi} {\unicode{x2A7D}} 4\hat{n}_{\mathrm{tot}}+2$. Therefore

\begin{align*}F_{\delta} &amp;{\unicode{x2A7E}} 1-\delta^{2}\bra{\psi}\qty(\qty(\frac{2}{(2r)^{2}} +\frac{20 \delta^{2}}{3 (2r)^{6}})\hat{n}_{\mathrm{tot}} + \frac{10\delta^{2}}{3(2r)^{6}}+\frac{3}{2 (2r)^{4}})\ket{\psi} \nonumber\\ &amp; = 1-\frac{\delta^{2}}{(2r)^{4}}\bra{\psi}\qty(\qty(2(2r)^{2} +\frac{20 \delta^{2}}{3 (2r)^{2}})\hat{n}_{\mathrm{tot}} + \frac{10\delta^{2}}{3(2r)^{2}}+\frac{3}{2})\ket{\psi}.\end{align*}

With the same parameters as before we get $F_{10^{-4}} {\unicode{x2A7E}} 0.999\,96$, which is significantly better than what we obtained with the simplified general bounds for BBP homodyne. The expressions derived in appendix C can in principle be used to obtain improved general bounds for the relevant regimes.

### 7.2. Estimating values of the characteristic function

Next we consider measuring the characteristic function of the Wigner distribution of a mode at a given point. The characteristic function of the Wigner function of the mode of quadrature $\hat{q}$ is $\chi(\gamma) = \langle D_{\gamma\boldsymbol{\alpha}}\rangle$ for complex γ. For γ=0, $\chi(\gamma) = 1$. If it were possible to implement an ideal quadrature measurement, we would estimate $\chi(\gamma)$ by repeatedly measuring $\hat{q}_{(\gamma/|\gamma|)\boldsymbol{\alpha}}$ and averaging $\mathrm e^{-\mathrm i|\gamma|\lambda}$ over the observed eigenvalues λ. Instead, we perform this average with the pulsed homodyne measurement outcomes. To determine the difference between $\chi(\gamma)$ and the computed average with pulsed homodyne measurements in the limit of a large number of measurements, we apply theorem 5.1 equation (17). In this equation, replace s and $\boldsymbol{\alpha}$ there by |γ| and $(\gamma/|\gamma|)\boldsymbol{\alpha}$ here. Because the pulsed homodyne observable $\hat{q}_{\delta}$ for δ>0 has discrete spectrum, it can in principle be implemented directly without added noise. Unbiased added noise does not affect the error in the mean. In particular, for determining the difference between the expectations of the target observable and the measured observable, we do not have to account for the added noise from, for example, subtracted photodiode measurements. Here, we do not consider the reduction in signal-to-noise from added noise. The difference between $\chi(\gamma)$ and the corresponding pulsed homodyne average is

\begin{align*} |\langle \mathrm e^{-\mathrm i\hat{q}_{\delta}|\gamma|}\rangle- \chi(\gamma)| &amp; = |\langle \mathrm e^{-\mathrm i\hat{q}_{\delta}|\gamma|}\rangle - \langle \mathrm e^{-\mathrm i\hat{q}|\gamma|}\rangle|^{2} \nonumber\\ &amp;{\unicode{x2A7D}} |\qty(\mathrm e^{-\mathrm i\hat{q}_{\delta}|\gamma|} - \mathrm e^{-\mathrm i\hat{q}|\gamma|})\ket{\psi}|^{2} \nonumber\\ &amp;{\unicode{x2A7D}} 4\delta^{2} |\gamma|^{2} \qty((1+|\gamma|^{2})\langle \hat{n}_{\mathrm{tot}}\rangle + |\gamma|^{2}) ,\end{align*}

where we applied equation (B5). Thus, to ensure a good estimate of the characteristic function at γ it suffices to choose δ so that $\delta^{2}|\gamma|^{4}(\langle \hat{n}_{\mathrm{tot}}\rangle {+1})$ and $\delta^{2}|\gamma|^{2}\langle \hat{n}_{\mathrm{tot}}\rangle$ are small. As a function of γ, the LO photon number required by this inequality scales as $|\gamma|^{4}$. For $\delta = 10^{-4}$ and $\langle\hat{n}_{\mathrm{tot}}\rangle {\unicode{x2A7D}} 5.0$, we can measure the characteristic function with error less than 0.038 for $|\gamma|{\unicode{x2A7D}} 20.0$, not accounting for added noise in the measurement.

If we use the bound from equation (D1) for standard pulsed homodyne, we get

\begin{align*} |\langle \mathrm e^{-\mathrm i\hat{q}_{\delta}|\gamma|}\rangle- \chi(\gamma)| {\unicode{x2A7D}} \delta^{2} |\gamma|^{2} \qty(\qty(2+\frac{4}{9}\delta^{2}|\gamma|^{4})\langle \hat{n}_{\mathrm{tot}}\rangle + \frac{2}{9}\delta^{2}|\gamma|^{4}+ \frac{1}{2}|\gamma|^{2}).\end{align*}

For the same bound of 20.0 on |γ|, we get a significantly smaller error bound of 0.0008. This is likely much lower than the signal-to-noise in the measurement. For $|\gamma|{\unicode{x2A7D}} 40.0$, the error bound is 0.0130.

### 7.3. Estimating moments

We can also perform the above exercise for measuring the k’th moment of the quadrature. The homodyne measurement has the correct expectation for k=1. For k=2, the difference between the expectations can be determined directly and is given by $\delta^{2}\langle \hat{n}_{\mathrm{tot}}\rangle$, see for example [1]. Here we consider even k with $k{\unicode{x2A7E}} 4$, so that for real x, $|x|^{k} = x^{k}$. Since the function $f_{0}(x) = x^{k}$ is not bounded, it is necessary to regularize it by applying proposition 5.4. There are many options for f(x). Consider a k-times differentiable function g(x) such that $\frac{d}{\mathrm dx^{k}}g(x)\big|_{x = 0} = k!$ and g(x) is the Fourier transform of $\tilde g(\kappa)$, where $\tilde g(\kappa)$ is the sum of an integrable function and a multiple of the delta function at 0. Equivalently, $\mathrm d\kappa\,\tilde g(\kappa)$ is the sum of a point measure at 0 and a bounded complex measure that is absolutely continuous with respect to Lebesgue measure. Then $f_{\lambda}(x) = g(\lambda x)/\lambda^{k}$ converges to $x^{k}$ uniformly on bounded intervals as λ goes to 0. Let $\Delta(x) = x^{k}-g(x)$. Then

\begin{align*} f_{0}(x)-f_{\lambda}(x)&amp; = \frac{1}{\lambda^{k}}\Delta(\lambda x), \nonumber\\ \mathrm d\kappa\tilde f_{\lambda}(\kappa) &amp; = \mathrm d\kappa\frac{1}{\lambda^{k+1}}\tilde g(\kappa/\lambda), \nonumber\\ \int_{\kappa}\mathrm d\kappa |\kappa|^{l}|\tilde f_{\lambda}(\kappa)| &amp; = \frac{1}{\lambda^{k+1}}\int_{\kappa}\mathrm d\kappa |\kappa|^{l}|\tilde g(\kappa/\lambda)| \nonumber\\ &amp; = \frac{1}{\lambda^{k-l}}\int_{\kappa}\mathrm d\kappa |\kappa|^{l}|\tilde g(\kappa)|.\end{align*}

To determine the constants needed to apply proposition 5.4 it suffices to determine bounds $\tilde g_{l}{\unicode{x2A7E}} \int_{\kappa}\mathrm d\kappa |\kappa|^{l}|\tilde g(\kappa)|$ for l=0,2. With these bounds, applying the proposition gives

\begin{align*} \left|\langle f(\hat{q}_{\delta})\rangle - \langle\hat{q}^{k}\rangle\right|^{2} {\unicode{x2A7D}} \frac{8}{\lambda^{2(k-1)}} \delta^{2}(\tilde g_{0}+\lambda^{2}\tilde g_{2}) \tilde g_{2}(\langle\hat{n}_{\mathrm{tot}}\rangle +1) + \frac{2}{\lambda^{2k}}\langle \Delta(\lambda \hat{q})^{2}\rangle .\end{align*}

It remains to choose g(x) and λ. Consider $g(x) = x^{k}/(1+x^{k})$. Then, because k is even, $\Delta(x) = x^{2k}/(1+x^{k}) {\unicode{x2A7D}} x^{2k}$. Therefore, the second summand in the bound of equation (83) is bounded by

\begin{align*} \frac{2}{\lambda^{2k}}\langle \Delta(\lambda \hat{q})^{2}\rangle {\unicode{x2A7D}} 2\lambda^{2k}\langle \hat{q}^{4k}\rangle.\end{align*}

We have $g(x) = 1-1/(1+x^{k})$ so that $\tilde g(\kappa)$ is a delta function at 0 minus the inverse Fourier transform $\tilde h(\kappa)$ of $h(x) = 1/(1+x^{k})$. We have

\begin{align*} \int_{\kappa} \mathrm d\kappa |\kappa|^{l}|\tilde g(\kappa)| = \delta_{0,l}+\int_{\kappa}\mathrm d\kappa|\kappa|^{l}|\tilde h(\kappa)|,\end{align*}

where $\delta_{0,l}$ is the Kronecker delta. The inverse Fourier transform of h(x) can be obtained from its partial fractions expansion. The function h(x) has poles at the non-degenerate roots of $p(x) = 1+x^{k}$, which are at $x_{j} = \mathrm e^{\mathrm i\pi2j/k}$ for $j = -(k-1)/2,-(k-3)/2,\ldots,-1/2,1/2,\ldots,(k-1)/2$, since k is even. We have $x_{j} = x_{-j}^{*}$. Thus $h(x) = \sum_{j = 0}^{k-1}\frac{c_{j}}{x-x_{j}}$, where the coefficients $c_{j}$ are given by $c_{j} = \frac{1}{p^{^{\prime}}(x_{j})} = \frac{1}{k x_{j}^{k-1}} = \frac{-x_{j}}{k}$. For $1/2{\unicode{x2A7D}} j{\unicode{x2A7D}} (k-1)/2$, the absolute value of the inverse Fourier transform of $x_{j}/(x-x_{j})$ is $\theta(-\kappa)\mathrm e^{\kappa \Im(x_{j})}$ where θ(y) is 0 for $y{\unicode{x2A7D}} 0$ and 1 otherwise. Similarly, for $-(k-1)/2{\unicode{x2A7D}} j{\unicode{x2A7D}} -1/2$, this absolute value is $\theta(\kappa)\mathrm e^{-\kappa \Im(x_{j})}$. Summing these absolute values over j and dividing by k gives

\begin{align*} |\tilde h(\kappa)| {\unicode{x2A7D}} \frac{1}{k}\sum_{j = 1/2}^{(k-1)/2} \mathrm e^{-|\kappa| \Im(x_{j})}.\end{align*}

For y>0, we have $\int_{\kappa\in\mathbb{R}}\mathrm e^{-|\kappa| y} = 2/y$ and $\int_{\kappa\in\mathbb{R}}\kappa^{2}\mathrm e^{-|\kappa|y} = 4/y^{3}$. For $1/2{\unicode{x2A7D}} j{\unicode{x2A7D}} (k-1)/2$, $\Im(x_{j}) = \sin(2\pi j/k){\unicode{x2A7E}} \sin(\pi/k)$. Consequently

\begin{align*} \int_{\mathbb{R}}|\tilde h(\kappa)| &amp;{\unicode{x2A7D}} \frac{2}{\sin(\pi/k)}, \nonumber\\ \int_{\mathbb{R}}\kappa^{2}|\tilde h(\kappa)| &amp;{\unicode{x2A7D}} \frac{4}{\sin(\pi/k)^{3}}.\end{align*}

Substituting back into equation (83)

\begin{align*}\left|\langle f(\hat{q}_{\delta})\rangle - \langle\hat{q}^{k}\rangle\right|^{2} &amp;{\unicode{x2A7D}} \frac{32}{\sin(2\pi/k)^{3}\lambda^{2(k-1)}} \delta^{2}\qty(1+\frac{2}{\sin(\pi/k)}+\lambda^{2}\frac{4}{\sin(\pi/k)^{3}})(\langle\hat{n}_{\mathrm{tot}}\rangle {+1}) \nonumber\\ &amp;\phantom{{}{\unicode{x2A7D}}{}\;\;\;} + 2\lambda^{2k}\langle \hat{q}^{4k}\rangle .\end{align*}

When applying this inequality, we do not know the value of $\langle \hat{q}^{4k}\rangle$, but we can replace this value by a conservatively estimated upper bound $q_{4k}{\unicode{x2A7E}} \langle\hat{q}^{4k}\rangle$. We assume that $q_{4k}{\unicode{x2A7E}} 1/2$ and minimize the right-hand side of the inequality over positive λ. For specific k and estimates $q_{4k}$, the minimization problem can be solved by solving for the critical points of the rational function of λ on the right-hand side numerically. Here we determine a generic upper bound for this minimum by approximation. For the estimated minimum to be less than 1 requires that $2\lambda^{2k}q_{4k} < 1$, which implies λ<1. Therefore $2\lambda^{2k}q_{4k}{\unicode{x2A7D}} 2\lambda^{2(k-1)}q_{4k}$. Since $k{\unicode{x2A7E}} 4$, $\sin(\pi/k){\unicode{x2A7D}} 1/\sqrt{2}$, so

\begin{align*} 1+\frac{2}{\sin(\pi/k)}+\lambda^{2}\frac{4}{\sin(\pi/k)^{3}} &amp;{\unicode{x2A7D}} \frac{1/(2\sqrt{2}) + 1 + 4\lambda^{2}}{\sin(\pi/k)^{3}} \nonumber\\ &amp;{\unicode{x2A7D}} \frac{6}{\sin(\pi/k)^{3}},\end{align*}

where the denominator of the last fraction was chosen to reduce expression complexity at the cost of a more conservative bound. We can set

\begin{align*} \lambda^{2(k-1)} = \frac{4\sqrt{6}\;\delta\sqrt{\langle\hat{n}_{\mathrm{tot}}\rangle {+1}}} {\sin(\pi/k)^{3}\sqrt{q_{4k}}}\end{align*}

to obtain

\begin{align*} \left|\langle f(\hat{q}_{\delta})\rangle - \langle\hat{q}^{k}\rangle\right|^{2} {\unicode{x2A7D}} 16\sqrt{6}\;\delta \frac{\sqrt{\langle\hat{n}_{\mathrm{tot}}\rangle {+1}}\sqrt{q_{4k}}} {\sin(\pi/k)^{3}}.\end{align*}

The square of the moment estimation error according to equation (91) scales as δ, while the explicit operator expansion for the moment of the pulsed homodyne observable indicates a scaling of order $\delta^{2}$ for $|\langle \hat{q}_{\delta}^{k}\rangle - \langle\hat{q}^{k}\rangle|$, for example, see [1]. For sufficiently small δ, the estimate is therefore poor. The explicit operator expansion involves large sums of operators of high degree in the mode operators whose expectations can be very large. As a result it is difficult to apply the explicit expansion directly, and for moderately small δ, the higher order terms can contribute significantly. The bounds above, while very conservative, have the advantage of being simple to apply, requiring only known bounds on $\langle \hat n\rangle$ and $\langle \hat{q}^{4k}\rangle$. For example, with $\delta = 10^{-4}$ and $\langle \hat{n}_{\mathrm{tot}}\rangle = 5$, for k=4 and k=6 the error according to the bound of equation (91) becomes $0.027\sqrt{q_{16}}$ and $0.077\sqrt{q_{24}}$, respectively.

We remark that because the fundamental inequality leading to the bound of equation (91) is based on proposition 5.4, it is not just a bound on the difference between expectations, but a bound on the difference between the states obtained by applying $f(\hat{q}_{\delta})$ and $f_{0}(\hat{q})$. This is one reason for the conservative power of δ. It may also be possible to choose better approximations f(x) of $f_{0}(x)$ to improve the scaling of the bound.

We do not perform the exercise of computing the error for moment measurements based on the bounds for standard pulsed homodyne in appendix D. This would require bounding the fourth moment of $\tilde g(\kappa)$ and involve a more difficult optimization over λ, but would likely significantly improve the error bounds. However, we do not expect the bound to have better scaling with δ and leave the determination of optimal bounds for future work.

### 7.4. CV teleportation

For an example involving conditional operations, we consider continuous variable (CV) teleportation as described in [13, 14]. We describe how to implement the results for conditional operations of section 6 to obtain bounds on the three contributions to infidelity of measurement-conditional operations given in the chain of equation (35). We do not instantiate the bounds numerically here. These bounds apply for generic measurement noise models. For measurement noise that can be treated as displacement noise before the conditional operations, it is possible to apply the simpler measurement fidelity results of section 5 as described and numerically implemented in the discussion of CV error correction below.

We first consider the middle step in the chain of equation (35). In CV teleportation, there are two quadrature measurements on independent modes. For each quadrature measurement, a conditional displacement of the form $\hat{U}_{B}(x) = \mathrm e^{\mathrm i\xi x \hat{p}_{B}}$ is applied, where $\hat{p}_{B}$ is one of two conjugate canonical quadratures of a mode of B, which are normalized as described after equation (2). The variable x is in the spectrum of $\hat{q}_{\delta}$, which is normalized according to our conventions and therefore a factor of $\sqrt{2}$ larger than the corresponding variable for canonical quadratures. These conditional displacements have v(x)=ξx with ξ>0 and w(x)=0 when applying proposition 6.2. The ideal scale factor ξ is ξ=1 because of the normalization of $\hat{q}$. In the references, this factor is $\sqrt{2}$ for canonical quadratures. The factor ξ may be modified by a gain to compensate for experimental effects. Because w(x)=0, we have $w_{l} = \tilde w_{l} = 0$. Therefore the operators defined in proposition 6.2 are $\hat{P}_{B} = 1$ and $\hat{Q}_{B} = 2\xi^{2}\hat{p}_{B}^{2}$. The difference between the state $\ket{\psi_{c,0}}$ obtained by applying the ideal quadrature-conditional displacement to initial state $\ket{\psi}$ and the state $\ket{\psi_{c,\delta}}$ obtained by applying the displacement conditional on the operator $\hat{q}_{\delta}$ for homodyne measurement with LO amplitude 1/δ is therefore given by

\begin{align*} \left| \ket{\psi_{c,\delta}}-\ket{\psi_{c,0}}\right|^{2} {\unicode{x2A7D}} 8\delta^{2}\xi^{2} \bra{\psi}(1+2\hat{p}_{B}^{2}\xi^{2})\hat{p}_{B}^{2} (\hat{n}_{\mathrm{tot}}+1) \ket{\psi} .\end{align*}

This bound applies to one of the two required conditional displacements. To obtain a bound on both of them, let $\hat{x}_{B}$ and $\hat{p}_{B}$ be the two conjugate canonical quadratures satisfying $[\hat{x}_{B},\hat{p}_{B}] = i$ of the mode of B. The first displacement applies $\hat{U}_{B}(x)$ conditioned on $\hat{q}_{a,\delta}$, and the second applies $\hat{V}_{B}(x) = \mathrm e^{\mathrm i\xi\hat{x}_{B}}$ conditioned on $\hat{q}_{b,\delta}$, where $\hat{q}_{a,\delta}$ and $\hat{q}_{b,\delta}$ are the observables for homodyne measurement with LO amplitude 1/δ associated with quadratures $\hat{q}_{a} = \hat{q}_{a,0}$ and $\hat{q}_{b} = \hat{q}_{b,0}$ of orthogonal modes. Let $\ket{\psi_{D,\delta}}$ denote the state after applying the two displacements conditioned on $\hat{q}_{a,\delta}$ and $\hat{q}_{b,\delta}$. We wish to bound

\begin{align*} \left| \ket{\psi_{D,\delta}}-\ket{\psi_{D,0}}\right| &amp; = \left| \mathrm e^{\mathrm i\xi \hat{q}_{b,\delta}\hat{x}_{B}}\mathrm e^{\mathrm i\xi\hat{q}_{a,\delta}\hat{p}_{B}}\ket{\psi} - \mathrm e^{\mathrm i\xi \hat{q}_{b}\hat{x}_{B}}\mathrm e^{\mathrm i\xi\hat{q}_{a}\hat{p}_{B}}\ket{\psi}\right| \nonumber\\ &amp;{\unicode{x2A7D}} \left|\mathrm e^{\mathrm i\xi\hat{q}_{b,\delta}\hat{x}_{B}}\qty(\mathrm e^{\mathrm i\xi\hat{q}_{a}\hat{p}_{B}}\ket{\psi}) - \mathrm e^{\mathrm i\xi\hat{q}_{b}\hat{x}_{B}}\qty(\mathrm e^{\mathrm i\xi\hat{q}_{a}\hat{p}_{B}}\ket{\psi})\right| \nonumber\\ &amp;\phantom{{\unicode{x2A7D}}\,|\,{}} + \left| \mathrm e^{\mathrm i\xi\hat{q}_{b,\delta}\hat{x}_{B}}\qty(\mathrm e^{\mathrm i\xi\hat{q}_{a,\delta}\hat{p}_{B}}\ket{\psi} -\mathrm e^{\mathrm i\xi\hat{q}_{a}\hat{p}_{B}}\ket{\psi})\right|.\end{align*}

For the second summand, the previously obtained bound applies as follows:

\begin{align*} \left| \mathrm e^{\mathrm i\xi\hat{q}_{b,\delta}\hat{x}_{B}}\qty(\mathrm e^{\mathrm i\xi\hat{q}_{a,\delta}\hat{p}_{B}}\ket{\psi} -\mathrm e^{\mathrm i\xi\hat{q}_{a}\hat{p}_{B}}\ket{\psi})\right|^{2} &amp; = \left|\mathrm e^{\mathrm i\xi\hat{q}_{a,\delta}\hat{p}_{B}}\ket{\psi} -\mathrm e^{\mathrm i\xi\hat{q}_{a}\hat{p}_{B}}\ket{\psi}\right|^{2} \nonumber\\ &amp;{\unicode{x2A7D}} 8\delta^{2}\xi^{2} \bra{\psi}(1+2\hat{p}_{B}^{2}\xi^{2})\hat{p}_{B}^{2} (\hat{n}_{a,\textrm{tot}}+1) \ket{\psi},\end{align*}

where $\hat{n}_{a,\textrm{tot}}$ is the total photon number of the mode of $\hat{q}_{a}$. For the first summand, the operator $\mathrm e^{\mathrm i\xi\hat{q}_{a}\hat{p}_{B}}$ does not affect the LO modes, so preserves vacuum on the LO modes. The bound of proposition 6.2 therefore applies, giving

\begin{align*} \left|\mathrm e^{\mathrm i\xi\hat{q}_{b,\delta}\hat{x}_{B}}\qty(\mathrm e^{\mathrm i\xi\hat{q}_{a}\hat{p}_{B}}\ket{\psi}) - \mathrm e^{\mathrm i\xi\hat{q}_{b}\hat{x}_{B}}\qty(\mathrm e^{\mathrm i\xi\hat{q}_{a}\hat{p}_{B}}\ket{\psi})\right|^{2} \kern-2in &amp;\nonumber\\ &amp;{\unicode{x2A7D}} 8\delta^{2}\xi^{2} \bra{\psi}\mathrm e^{-\mathrm i\xi\hat{q}_{a}\hat{p}_{B}} (1+2\hat{x}_{B}^{2}\xi^{2})\hat{x}_{B}^{2} (\hat{n}_{b,\textrm{tot}}+1) \mathrm e^{\mathrm i\xi\hat{q}_{a}\hat{p}_{B}}\ket{\psi}.\end{align*}

Since $\mathrm e^{-\mathrm i\xi\hat{q}_{a}\hat{p}_{B}}\hat{x}_{B}\mathrm e^{\mathrm i\xi\hat{q}_{a}\hat{p}_{B}} = \hat{x}_{B}- \xi\hat{q}_{a}$, we now have

\begin{align*} \left| \ket{\psi_{D,\delta}}-\ket{\psi_{D,0}}\right|^{2} &amp;{\unicode{x2A7D}} 16\delta^{2}\xi^{2}\bra{\psi}\Big( (1+2(\hat{x}_{B}- \xi\hat{q}_{a})^{2}\xi^{2}) \times(\hat{x}_{B}- \xi\hat{q}_{a})^{2} (\hat{n}_{b,\textrm{tot}}+1) \nonumber\\ &amp;\quad + (1+2\hat{p}_{B}^{2}\xi^{2})\hat{p}_{B}^{2} (\hat{n}_{a,\textrm{tot}}+1) )\Big)\ket{\psi}\end{align*}

with the extra factor of two accounting for the cross terms from squaring that are dropped. To apply the bound it suffices to use conservative upper bounds for the expectations of the products of quadrature and number operators that are obtained when multiplying out the terms in the bound. The bound can be expressed in terms of correlated moments of number operators by bounding $\hat{x}_{B}^{2}$ and $\hat{p}_{B}^{2}$ above by $2\hat{n}_{B}+1$, applying equation (B6) with the normalization for conjugate quadratures.

Next we consider the last step of equation (35). To obtain its contribution to infidelity, we can apply proposition 6.5. We can model homodyne measurements with photodiodes such as in [18] with $h(z|x) = \mathrm e^{-(z-x)^{2}/(2\delta^{2}\sigma^{2})}/(\sqrt{2\pi}\delta\,\sigma)$ in proposition 6.5. Accordingly, in this proposition, $K_{1} = \xi$ and $K_{2} = \delta^{2}\sigma^{2}$.

\begin{align*} \left| \ket{\tilde\psi_{m,\delta}}-\ket{\psi_{m,\delta}}\right|^{2} {\unicode{x2A7D}} 2\xi^{2}\delta^{2}\sigma^{2}\langle (\hat{n}_{\mathrm{tot}}+1/2 )^{2}\rangle^{1/2}.\end{align*}

Because of the two measurements, the distance from this expression is added twice to the bound on the distance in the previous paragraph, once for $\hat{n}_{\mathrm{tot}} = \hat{n}_{a,\textrm{tot}}$ and once for $\hat{n}_{\mathrm{tot}} = \hat{n}_{b,\textrm{tot}}$.

For the contribution from the first step of the chain of equation (35) we can apply theorem 5.2 to the two measurements with the conditional displacements applied perfectly and unitarily, conditional on the target quadratures before coupling to the apparatus. For applying theorem 5.2, the state being measured corresponds to the state after ‘teleporting’ unitarily and does not actually occur in a teleportation experiment. For Gaussian added noise, we can apply equation (74) twice, where $\langle \hat{n}_{\mathrm{tot}}\rangle$ must be estimated for the state obtained after the unitary conditional displacements and each of the two measurement modes.

We do not instantiate the above bounds numerically here. Such instatiation involves a unenlightening and non-trivial if routine substitution of formulas following the above instructions. Furthermore, the most relevant applications involve balanced pairs of photodiodes adding Gaussian noise. Because this added noise is equivalent to displacement noise before the measurement. If such displacement noise has already been accounted for in the error budget, it is possible to bypass the above bounds and directly apply the results of section 5 or appendix D for post-measurement states. This technique is used below to analyze CV error correction with a closely related measurement configuration, and the bounds obtained there can be applied to teleportation with few modifications.

### 7.5. CV error correction

We consider GKP error correction for qubits encoded in a quantum mode. There are two standard ways to implement GKP error correction, Steane error correction and teleported error correction. Versions of these protocols are shown in figure 10 of [20]. The protocol for Steane error correction involves the same measurements and shifts as for teleportation, so the analysis for teleportation given above can be applied given bounds on expectations of correlated photon number moments computed for the appropriate input states. Teleported error correction does not require explicitly correcting shifts. However, the logical Pauli frame is adjusted according to the measurement outcomes, which affects future operations. For the purpose of applying the fidelity analysis for conditional operations, it is necessary to apply outcome-conditional logical operations to reset the frame and remove the dependence of future operations on the measurement outcomes. Because these logical operations are not simple linear shifts, a direct application of our bounds requires regularization with lemma 6.3. We do not perform this exercise here. Instead we take advantage of the equivalence of added noise in the measurement outcomes to displacement noise on the incoming GKP qubit. In particular, for GKP error correction based on quadrature measurements, added Gaussian noise arising from the use of balanced photodiodes as detectors according to equation (73) corresponds to added Gaussian displacements noise. Such noise can be included in the analysis of the quality of the error-correction process with ideal quadrature-conditional operations. Therefore, the additional infidelity from using homodyne measurements with finite LO amplitude can be accounted for by the measurement fidelity analysis of section 5. By monotonicity of fidelity, subsequent operations conditional on the measurement outcomes do not increase this infidelity. In the remainder of this section we formalize this approach to bounding the added infidelity in GKP error correction due to finite LO amplitude when implementing the homodyne measurements.

Following the notation introduced at the beginning of section 6, let $\ket{\psi}$ be the initial state of modes $\boldsymbol{a}$, B and the LO modes $\boldsymbol{b}$, where $\ket{\psi}$ is vacuum on the LO modes. In the case of GKP error correction, modes of system B carry encoded information, while modes $\boldsymbol{a}$ contain syndrome information to be used for error correction or frame tracking, where the currently relevant syndrome information is carried by the quadrature $\hat{q}$. In principle, the ideal error correction procedure or future frame-dependent unitaries implement unitaries conditional on $\hat{q}$. Let $\hat{p}$ be a quadrature of the mode of $\hat{q}$ such that the displacement $\hat{D}_{x} = \mathrm e^{\mathrm i x\hat{p}}$ satisfies $\hat{D}_{-x}\hat{q}\hat{D}_{x} = \hat{q}-x$. For $\hat{q} = \hat{q}_{\alpha}$ as defined in section 3, we have $\hat{p} = \hat{q}_{i\alpha/(2|\alpha|^{2})}$. In the next two paragraphs, we show that additive measurement noise with probability distribution $\mu(x)\mathrm dx$ is equivalent to random displacements $\hat{D}_{x}$ acting on the mode of $\hat{q}$, where x has probability distribution $\mu(x)\mathrm dx$. We can assume without loss of generality that $\boldsymbol{a}$ consists of only one mode, mode a, with quadratures $\hat{q}$ and $\hat{p}$.

In general, a dilated, unitary model for the displacement noise is obtained by adding the quantum system M consisting of a one-dimensional system with position operator $\hat{x}_{M}$ and initial wavefunction $\ket{\sqrt{\mu(\boldsymbol{\cdot})}}$ in the position basis for $\hat{x}_{M}$. The system M is coupled to the mode of $\hat{q}$ by $\mathrm e^{\mathrm i\hat{x}_{M}\hat{p}}$. The random displacement noise is obtained after tracing out M. Future conditional operations applied after displacement noise are of the form $\hat{U}(\hat{q})$ and applied after the coupling to M. Since $\hat{q}\mathrm e^{\mathrm i\hat{x}_{M}\hat{p}} = \mathrm e^{\mathrm i\hat{x}_{M}\hat{p}}(\hat{q}-\hat{x}_{M})$, the conditional operations $\hat{U}(\hat{q})$ after the coupling are equivalent to the corresponding operations $\hat{U}(\hat{q}-\hat{x}_{M})$ conditioned on $\hat{q}-\hat{x}_{M}$ before the coupling. Given that the mode a and the system M play no further role besides these conditional operations, the dilated model is equivalent to initializing M in state $\ket{\sqrt{\mu(\boldsymbol{\cdot})}}$, and applying the needed conditional operations conditional on $\hat{q}-\hat{x}_{M}$, without any other operations applied to mode a or apparatus M.

We compare random displacement noise before $\hat{q}$-conditional operations to an instance of the unitary measurement model of section 4 with operations conditioned on the apparatus, whose quantum system is also M. For the unitary measurement model, we prepare M with the same initial wavefunction $\ket{\sqrt{\mu(\boldsymbol{\cdot})}}$. But M is coupled to the mode of $\hat{q}$ by $\mathrm e^{\mathrm i\hat{p}_{M}\hat{q}}$, where $\hat{p}_{M}$ is the momentum operator for M scaled so that the displacement $\hat{D}_{y} = \mathrm e^{\mathrm iy\hat{p}_{M}}$ satisfies $\hat{D}_{-y}\hat{x}_{M}\hat{D}_{y} = \hat{x}_{M}-y$. Future operations are of the form $\hat{U}(-\hat{x}_{M})$, conditioned on $-\hat{x}_{M}$ rather than $\hat{q}$. Here, to simplify the desired correspondence, we chose the apparatus coupling so that the measured quantity is encoded in the eigenvalues of $-\hat{x}_{M}$ rather than $\hat{x}_{M}$. Since $(-\hat{x}_{M})\mathrm e^{\mathrm i\hat{p}_{M}\hat{q}} = \mathrm e^{\mathrm i\hat{p}_{M}\hat{q}}(-\hat{x}_{M}+\hat{q})$, these conditional operations are equivalent to operations $\hat{U}(\hat{q}-\hat{x}_{M})$ conditioned on $\hat{q}-\hat{x}_{M}$ before the apparatus coupling. The situation is therefore equivalent to the dilated random displacement model, except for the irrelevant different processing of mode a and apparatus M after the conditional operations acting before the coupling.

The above shows that added displacement noise in measurement according to the unitary measurement model with subsequent apparatus-conditional unitaries is equivalent to added displacement noise before the corresponding quadrature-conditional unitaries. This added noise can be accounted for in the analysis of the performance of GKP error correction. Added infidelity due to the difference between $\hat{q}_{\delta}$ and $\hat{q}$ at finite LO amplitude can then be attributed to the infidelity between the global state after apparatus coupling with the ideal coupling $\mathrm e^{\mathrm i\hat{p}_{M}\hat{q}}$ and the state obtained with the coupling $\mathrm e^{\mathrm i\hat{p}_{M}\hat{q}_{\delta}}$, where the apparatus initial state reflects the detector added noise. The strategy is then to first consider GKP error correction when the incoming state has been affected by physical displacement noise with a given variance. We add to the physical noise the effective displacement noise contributed by noisy measurements of the ideal quadratures and bound the infidelity of the logical information for ideal error correction, where in the ideal error correction, operations are conditional unitaries conditioned on the ideal quadratures. Second, we bound the infidelity between the state obtained with the ideal apparatus coupling, and the state obtained with the apparatus coupling with the homodyne observable at LO amplitude 1/δ, where the apparatus initial state reflects the detector added noise. For this bound, we can use equation (74). For Gaussian added noise described by $\mu(x) = \frac{1}{r\sqrt{2\pi}}\mathrm e^{-x^{2}/(2r^{2})}$, we can apply equation (75). For a worst-case bound on the overall infidelity, one can then combine the two infidelity bounds. Instead, we determine the LO amplitude and resolution required to meet separate infidelity budgets for the two contributions to infidelity. The resolution-amplitude tradeoffs for constant-added-noise photodiodes discussed after equation (75) apply here, but now become a tradeoff between added displacement noise that must be handled by the GKP codes, and infidelity in the post-measurement state due to finite LO amplitude.

**Figure 4.**
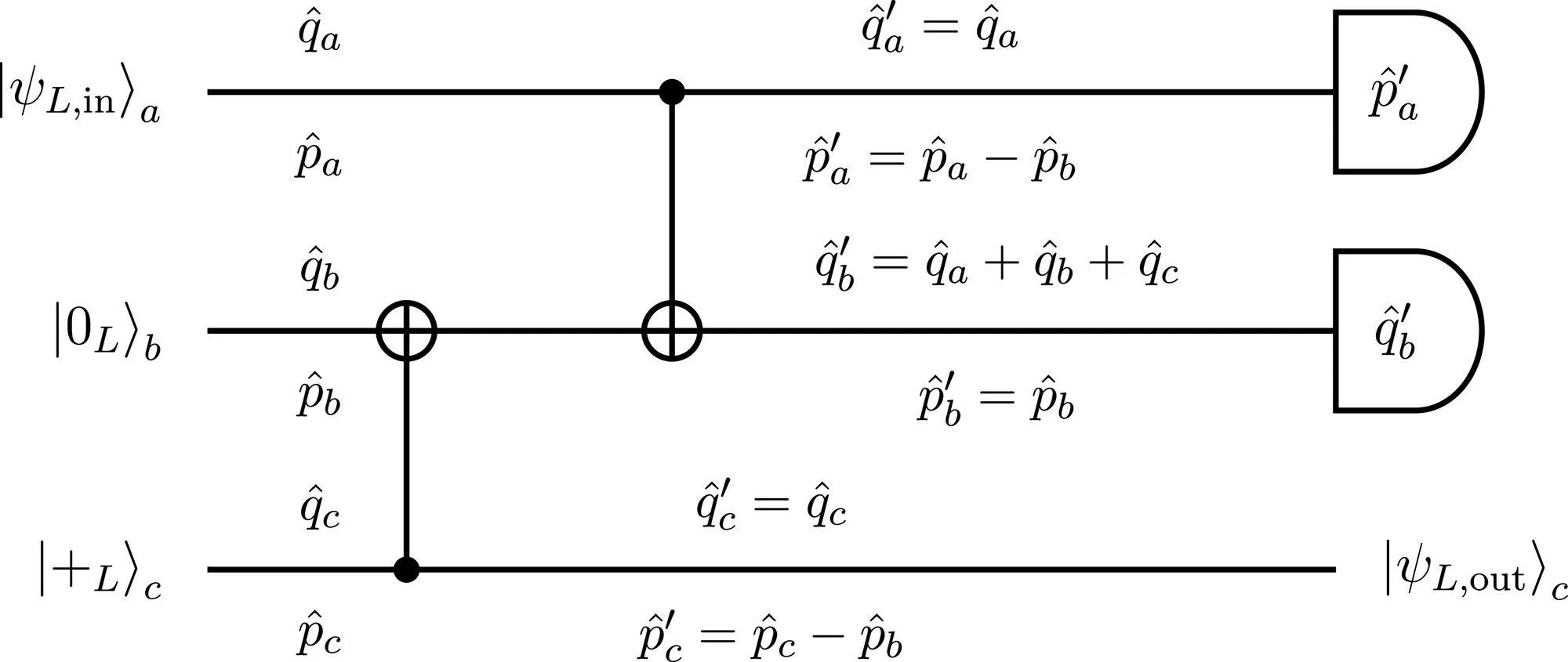
GKP teleported error correction. The top line carries mode a which contains the incoming GKP qubit, the bottom line carries mode c which contains the outgoing GKP qubit. The mode operators for the incoming modes and for the modes after coupling are shown with their relationships. The couplings are denoted by CNOT symbolic gates. These are active linear optical. For example, the one that couples mode c to mode b commutes with $\hat{q}_{c}$ and $\hat{p}_{b}$, and the quadratures $\hat{q}_{b}^{^{\prime\prime}}$ and $\hat{p}_{c}^{^{\prime\prime}} = \hat{p}_{c}^{^{\prime}}$ are given by $\hat{q}_{b}+\hat{q}_{c}$ and $\hat{p}_{c}-\hat{p}_{b}$ in terms of the incoming quadratures. The diagram is based on figure 10 of [20].

To estimate the measurement fidelities and account for the effect of the displacement noise on GKP encoded information we consider teleported error correction with the couplings shown in figure 4, see also figure 10 of [20]. Teleported error correction requires coupling the mode containing a GKP encoded qubit to two ancillas prepared independently in GKP encoded states, followed by measuring quadratures. The measured quadratures and their associated displacement noise can be expressed in terms of the quadratures of the three modes a,b,c consisting of the incoming GKP qubit in mode a and two ancilla GKP qubits in modes b and c. Let $\hat{q}_{u},\hat{p}_{u}$ be the incoming GKP conjugate quadratures of mode u with $u\in\{a,b,c\}$, where we use the normalization conventions from section 3 for these quadratures. The teleported error correction circuit is shown in figure 4. The quadratures $\hat{q}_{a}^{^{\prime}}$ and $\hat{p}_{b}^{^{\prime}}$ are intended to be measured and subsequently used for error correction or Pauli frame tracking. We describe such actions as conditional correction unitaries. We analyze the effect of displacement noise generated by the relevant quadratures, propagating the effect to the incoming and outgoing GKP qubits, where we add the effect on the outgoing GKP qubit to the error on incoming GKP qubit in the next error-correction cycle. From this analysis, we can determine the performance of the error-correction circuit in terms of noise acting only on the incoming GKP qubit.

To propagate the displacement noise to the incoming GKP qubit, we consider each displacement-generating quadrature. For this purpose we express the quadratures acting just before measurements in terms of incoming quadratures as shown in figure 4. The identities represent equivalent actions in terms of the overall effect on the circuit.

• Displacements generated by $\hat{q}_{b}$: We can write
  \begin{align*} \hat{q}_{b} = \hat{q}_{b}^{^{\prime}} - \hat{q}_{a}-\hat{q}_{c} = \hat{q}_{b}^{^{\prime}}-\hat{q}_{a}-\hat{q}_{c}^{^{\prime}}.\end{align*}
  Displacements generated by $\hat{q}_{b}^{^{\prime}}$ have no effect on the circuit since $\hat{q}_{b}^{^{\prime}}$ is measured. Thus, displacements generated by $\hat{q}_{b}$ are equivalent to simultaneous displacements of the incoming GKP qubit according to $\hat{q}_{a}$ and the outgoing GKP qubit by $\hat{q}_{c}^{^{\prime}}$.
• Displacements generated by $\hat{p}_{b}$: We can write
  \begin{align*} \hat{p}_{b} = -\hat{p}_{a}^{^{\prime}} + \hat{p}_{a}.\end{align*}
  Displacements generated by $\hat{p}_{a}^{^{\prime}}$ have no effect since $\hat{p}_{a}^{^{\prime}}$ is measured. Thus, displacements generated by $\hat{p}_{b}$ are equivalent to displacements of the incoming GKP qubit according to $\hat{p}_{a}$.
• Displacements generated by $\hat{q}_{c}$: Since $\hat{q}_{c} = \hat{q}_{c}^{^{\prime}}$, these displacements act directly on the outgoing GKP qubit.
• Displacements generated by $\hat{p}_{c}$: We can write
  \begin{align*} \hat{p}_{c} = \hat{p}_{c}^{^{\prime}}-\hat{p}_{a}^{^{\prime}}+\hat{p}_{a}.\end{align*}
  The contribution from $\hat{p}_{a}^{^{\prime}}$ is invisible, that from $\hat{p}_{a}$ acts on the incoming GKP qubit, and that from $\hat{p}_{c}^{^{\prime}}$ on the outgoing GKP qubit.
• Displacements generated by $\hat{q}_{a}^{^{\prime}}$: This is measurement-added noise in the measurement of $\hat{p}_{a}^{^{\prime}}$. Since $\hat{q}_{a}^{^{\prime}} = \hat{q}_{a}$, this is a displacement of the incoming GKP qubit according to $\hat{q}_{a}$.
• Displacements generated by $\hat{p}_{b}^{^{\prime}}$: This is measurement-added noise in the measurement of $\hat{q}_{b}^{^{\prime}}$. We can write
  \begin{align*} \hat{p}_{b}^{^{\prime}} = \hat{p}_{b} = -\hat{p}_{a}^{^{\prime}} + \hat{p}_{a},\end{align*}
  which generates displacements equivalent to displacements of the incoming GKP qubit according to $\hat{p}_{a}$.

We consider square GKP qubits. With respect to conjugate canonical quadratures $\hat{x}$ and $\hat{p}$ normalized as described after equation (2) and satisfying $[\hat{x},\hat{p}] = i$, the ideal but improper logical states $\ket{s}$ for s=0,1 are joint improper eigenstates of the stabilizers $\mathrm e^{\mathrm i2\sqrt{\pi}\hat{x}}$ and $\mathrm e^{\mathrm i2\sqrt{\pi}\hat{p}}$ that in the position space for $\hat{x}$ can be written as $\sum_{j = -\infty}^{\infty}\ket{(2j+s)\sqrt{\pi}}$. Our bounds are expressed in terms of quadrature operators $\hat{q}$ that are normalized according to $\hat{q} = \sqrt{2}\hat{x}$ or $\hat{q} = \sqrt{2}\hat{p}$, depending on which quadrature is being measured. The measurement added noise is expressed in terms of measurements of quadratures with the latter normalization. Thus noise with variance $r^{2}$ in measuring $\hat{q}$ corresponds to noise with variance $r^{2}/2$ in measuring $\hat{x}$ or $\hat{p}$. Below we refer to numerical calculations of GKP error correction fidelities with respect to displacement noise. For these calculations, displacement noise is with respect to canonical quadratures. So Gaussian noise with variance $\sigma_{g}^{2}$ for a canonical quadrature is equivalent to noise with variance $2\sigma_{g}^{2}$ for the quadrature normalized according to our conventions. In general, with the quadrature definitions of section 3, the variances are related by $\langle (\hat{q}_{\alpha}-\langle\hat{q}_{\alpha}\rangle)^{2}\rangle = 2|\alpha|^{2}\langle(\hat{q}_{1/\sqrt{2}}-\langle\hat{q}_{1/\sqrt{2}}\rangle)^{2}\rangle$, where $\hat{q}_{1/\sqrt{2}}$ is canonical. For the remainder of this analysis, variances with the Greek letter σ refer to displacement noise variances for canonical quadratures. We convert measurement added noise with variance $r^{2}$ for measuring $\hat{q}$ normalized according to our conventions to canonical displacement noise with variance $r^{2}/2$ before the measurement.

We assume that the incoming GKP qubit and ancilla qubits both experience physical Gaussian isotropic displacement noise with marginal variance $\sigma_{0}^{2}$ for each canonical quadrature. In consideration of the equivalences given above, we can redistribute it and attribute it to noise on the incoming GKP qubit with no noise acting on the ancillas. For this, we add the variances of the noisy displacement for each quadrature from redistributing noise in the previous error-correction cycle acting on the then outgoing and now incoming GKP qubit to the physical noise and the redistributed noise variances from the current error-correction cycle. The resulting noisy displacements add independently from each source and after accounting for redistributed displacement noise from both the previous and the present error-correction cycle the noise remains isotropic. Adding the variances from the redistributed physical noise, we get a total variance of $3\sigma_{0}^{2}$ in each canonical quadrature acting only on the incoming GKP qubit. For displacement noise generated by canonical $\hat{q}$ in figure 4, this variance is obtained by adding the initial noise on the GKP qubit coming in from the previous error correction cycle, the noise on this qubit propagated forward from the previous cycle’s $\hat{q}_{b}$ displacement noise, and the noise propagated from the current cycle’s $\hat{q}_{b}$ displacement noise. The total displacement noise generated by $\hat{p}$ can be determined similarly. Let r be the resolution of homodyne measurement, accounting for measurement-added noise and the LO amplitude. The measurement-added noise contributes a variance $r^{2}/2$ in the canonical quadratures. The total noise variance affecting the fidelity of the error-correction cycle with ideal quadrature-conditional correction unitaries is therefore $\sigma_{\mathrm{noise}}^{2} = 3\sigma_{0}^{2}+r^{2}/2$.

It is necessary to estimate the expected photon numbers in the measured modes. For the contribution to infidelity from the implemented measurement, it is necessary to calculate the expected photon numbers with respect to the physical incoming states, without redistribution of the physical noise or applying the equivalence between added measurement noise and displacement noise to the current error-correction cycle. For the incoming GKP qubit, it is necessary to take into account the effects from the previous cycle and possible actions. We assume that these effects and actions are well represented by the physical noise and a displacement that might be used to implement a logical Pauli gate with displacements to one or both of the quadratures of the GKP qubit.

We write $\hat{n}^{^{\prime}}_{a}$ and $\hat{n}^{^{\prime}}_{b}$ for the number operators of the modes measured for $\hat{p}_{a}^{^{\prime}}$ and $\hat{q}_{b}^{^{\prime}}$. For applying our bounds, these quadratures are normalized according to our conventions. From the equivalences in figure 4 and applying the identity in equation (B6),

\begin{align*}\langle \hat{n}^{^{\prime}}_{a}\rangle &amp; = \frac{1}{4}\left\langle\hat{q}_{a}^{2}+ (\hat{p}_{a}-\hat{p}_{b})^{2}-2\right\rangle \nonumber \\ &amp; = \frac{1}{4}\left\langle\hat{q}_{a}^{2}+ \hat{p}_{a}^{2}+\hat{p}_{b}^{2} - 2\hat{p}_{a}\hat{p}_{b}-2\right\rangle ,\end{align*}

and similarly

\begin{align*}\langle \hat{n}^{^{\prime}}_{b}\rangle &amp; = \frac{1}{4}\left\langle (\hat{q}_{a}+\hat{q}_{b}+\hat{q}_{c})^{2} + \hat{p}_{b}^{2} -2\right\rangle \nonumber \\ &amp; = \frac{1}{4}\qty( \langle\hat{q}_{a}^{2}\rangle+\langle\hat{q}_{b}^{2}\rangle +\langle\hat{p}_{b}^{2}\rangle+\langle\hat{q}_{c}^{2}\rangle + 2\qty( \langle\hat{q}_{a}\hat{q}_{b}\rangle +\langle\hat{q}_{a}\hat{q}_{c}\rangle +\langle\hat{q}_{b}\hat{q}_{c}\rangle) -2) .\end{align*}

The three input modes are independent.

**Figure F1.**
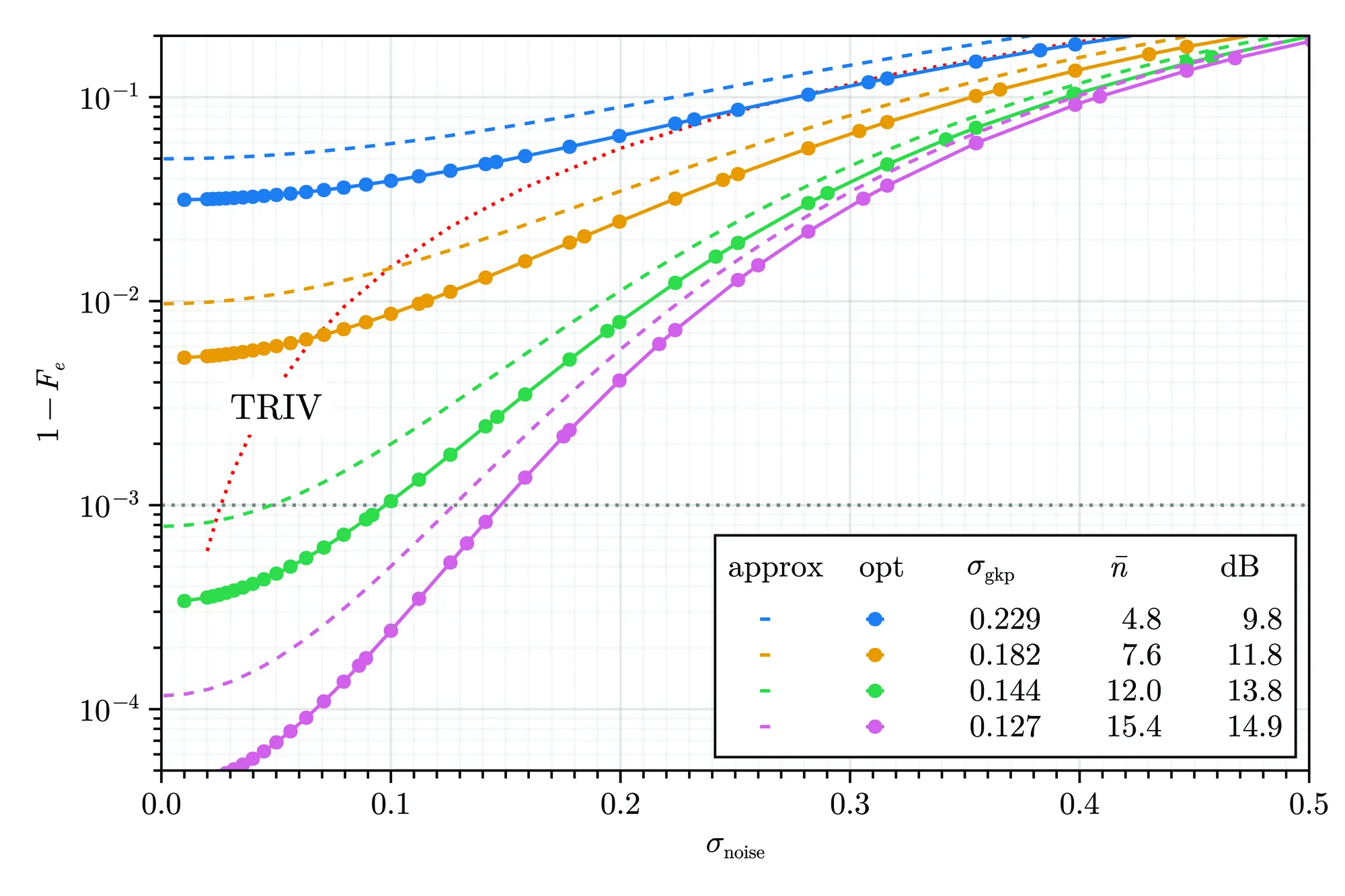
Entanglement infidelity as a function of displacement noise strength for square GKP qubits. The displacement noise is Gaussian and isotropic with variance $\sigma_{\mathrm{noise}}^2$. Each series corresponds to a GKP code with variance $\sigma_{\mathrm{gkp}}^2$, average photon number $\bar{n}$ of the code space projector, and the corresponding squeezing in decibels (dB). According to [30], for general GKP fault tolerance, a squeezing safely above $9.75\;\textrm{dB}$ is sufficient. See figure S15 of the supplementary information of the reference, which shows that the logical error rate at the threshold is greater than $10\;{\%}$ and error rates for reasonable coding distances do not drop below $.1\;{\%}$ for squeezings less than $10\;\textrm{dB}$. [31] claims $15\;\textrm{dB}$ optical squeezing. We also include the TRIV curve for the trivial qubit code spanned by the oscillator ground $\ket{0}$ and first excited $\ket{1}$ states. The solid lines correspond to the numerically optimized recovery while the dashed lines correspond to an analytic approximation for the teleported error correction circuit in figure 4. For the analytic approximation, $\sigma_{\mathrm{gkp}}^{2}$ is treated as an effective noise on ideal GKP qubits. Because each of the two ancillas has the same effective noise, by the error propagation analysis of section 7.5, this corresponds to an effective noise variance of $3\sigma_{\mathrm{gkp}}^{2}$ on the incoming GKP qubit with ideal ancillas. For the curves shown, noise with variance $\sigma_{\mathrm{noise}}^{2}$ is added to the incoming GKP qubit only. For the numerically optimized recovery, there are no ancillas. The incoming GKP qubit is the same as for the analytic approximation with squeezing corresponding to $\sigma_{\mathrm{gkp}}$. For comparing to the analytic approximation, we optimized the recovery for an added displacement noise with variance $\sigma_{\mathrm{opt}}^{2} = 2\sigma_{\mathrm{gkp}}^{2}+\sigma_{\mathrm{noise}}^{2}$.

For reference, we use the performance data for finite-energy square GKP qubits shown in appendix F figure F1, where we use the curves that show an analytic approximation for the entanglement fidelity of teleported error-correction as a function of the total redistributed displacement noise variance acting on the incoming GKP qubit. The error-free states for these square GKP qubits have close to zero-mean quadratures. Let $\bar{n}$ be the expected number of photons for a particular such qubit. Since, with our conventions, $4(\bar n+1/2) = \langle\hat{q}^{2}\rangle+\langle\hat{p}^{2}\rangle$, and the square GKP qubits are approximately symmetric, we use the estimate $v = \langle\hat{q}^{2}\rangle \approx \langle\hat{p}^{2}\rangle \approx 2\bar n+1$. We choose the square GKP qubit with $\bar{n}=4.8$ for illustration. The squeezing associated with this GKP qubit is 9.8. We fix an infidelity bound of $\epsilon_{\mathrm{ec}} = 0.06$ for the entanglement infidelity according to curves in figure F1. We expect our bounds to be quite conservative and allow $\epsilon_{m} = 0.02$ for the sum of the two infidelities from the two quadrature measurements. We further expect the three infidelities to add incoherently for an overall infidelity bounded by 0.080. To determine $\sigma_{\mathrm{noise}}$ we consult the entanglement fidelity curves for the GKP qubit with $\bar{n}=4.8$ and let $\sigma_{\mathrm{noise}}$ be a lower bound on the noise standard deviation at which the curve crosses the chosen entanglement fidelity bound of 0.06. Thus we set $\sigma_{\mathrm{noise}} = 0.1$. Assuming a bound of 0.05 on the physical displacement noise $\sigma_{0}$, we solve for r in the expression for $\sigma_{\mathrm{noise}}$ to get r=0.071.

Based on the assumed values of $\bar{n}$, we obtain v=10.6 for the expectations of the square quadratures in equations (102) and (103) before accounting for displacements from logical operations and noise. This value is obtained from the expected photon number by applying equation (B6), assuming the expectations of the quadrature are 0 and the variances of the two quadratures are identical. The expectations of the square quadratures of the incoming GKP qubit and the ancillas each increase by the added noise variance of $2\sigma_{0}^{2}$, accounting for the normalization used here. The incoming GKP qubit may have been displaced by a logical operation Pauli gate, which can be implemented by displacements of magnitude$\sqrt{\pi}$ of the canonical variables. This can add 2π to the expectation of each square quadratures of the incoming GKP qubit in equations (103) and (102), since the mean of the quadratures is close to zero before the operation. By independence of the modes, we obtain the bounds

\begin{align*} \langle\hat{n}^{^{\prime}}_{a}\rangle &amp;{\unicode{x2A7D}} \frac{1}{4}\qty(3v - 2 +3\sigma_{0}^{2}+ 4\pi) = 10.6, \nonumber\\ \langle\hat{n}^{^{\prime}}_{b}\rangle &amp;{\unicode{x2A7D}} \frac{1}{4}\qty(4v-2 +4\sigma_{0}^{2}+2\pi) ) = 11.7.\end{align*}

The bound on the measurement infidelity for one quadrature measurement is expressed in terms of the expected photon number, the resolution, and the LO photon number $\delta^{2}$ in equation (79). We require that the sum of the bounds on the quadrature measurement infidelities is less than the allowance $\epsilon_{m}$. This gives the inequality

\begin{align*} \epsilon_{m} {\unicode{x2A7E}} \frac{\delta^{2}}{(2r)^{4}}\bra{\psi}\qty(\qty(4(2r)^{2} +\frac{40 \delta^{2}}{3 (2r)^{2}})(\langle\hat{n}^{^{\prime}}_{q}\rangle+\langle\hat{n}^{^{\prime}}_{p}\rangle) + \frac{20\delta^{2}}{3(2r)^{2}}+3)\ket{\psi}.\end{align*}

Let $\delta_{m}$ be the value of δ that achieves equality in the inequality above. Provided that $\delta{\unicode{x2A7D}}\delta_{m}$, the sum of the measurement infidelities is less than our target $\epsilon_{m}$. To solve for $\delta_{m}$, we set the resolution and expected photon numbers according to the previously obtained values. The identity to be solved is

\begin{align*} 2.000\times 10^{-2} = 1.197\times 10^{4}\delta_{m}^{2} + 3.808\times 10^{7}\delta_{m}^{4}.\end{align*}

The degree four term in $\delta_{m}$ contributes little. We neglect it for $ \delta_{m}^{2} = 1.7\times 10^{-6} $. If we do not neglect the degree four term, substituting this value of $\delta_{m}^{2}$ results in a value of $2.010\,632\times 10^{-2}$ for the right-hand sides of equation (106). The value of $\delta_{m}^{2}$ found corresponds to a minimum required LO photon number of $n_{\mathrm{LO}} = 6.0\times 10^{5}$. In terms of equivalent photodiode added noise, the resolution and LO amplitude corresponds to an average added noise of 54.7 electrons. The bounds remain satisfied if we increase the LO photon number by decreasing $\delta^{2}$ while keeping the resolution r constant. Since $r = \delta\sigma$, increasing the LO photon number by a factor of k corresponds to an increase in equivalent photodiode added noise by a factor of $\sqrt{k}$. Thus, if the actual added noise is larger than the one computed above, it suffices to increase the LO photon number accordingly. If the actual added noise is smaller than the computed one, we can, conservatively, add noise to the measurement outcomes to compensate and ensure the bounds. This mainly guarantees that there are no problems with conditional actions that depend on fine details of the measurement outcomes, for example such actions that depend discontinuously on the outcomes.

We conclude our analysis with table 1 showing the parameters and bounds for a few choices of GKP qubits and fidelity bounds.

**Table 1.** table of required homodyne parameters for square GKP qubits and fidelity bounds. The GKP qubit parameters $\bar{n}$ with corresponding squeezing in $\;\textrm{db}$ and entanglement fidelity performances are from figure F1. For each GKP qubit, we choose a combination of noise variance $\sigma_{\mathrm{noise}}$ and entanglement infidelity $\epsilon_{\mathrm{ec}}$ for which the analytic approximation according to the figure gives an entanglement infidelity close to and less than $\epsilon_{\mathrm{ec}}$. We then choose an allowance for the physical noise $\sigma_{0}$ on the GKP qubit and the prepared ancillas. The noise variance $\sigma_{0}^{2}$ must be less than $\sigma_{\mathrm{noise}}^{2}/3$. From this we determine the required measurement resolution r. We then choose an allowance for the sum $\epsilon_{m}$ of the measurement infidelities from the homodyne measurements. Assuming no coherent contribution when chaining the infidelities, the overall infidelity is $\epsilon_{\mathrm{ec}}+\epsilon_{m}$. From these parameters, we compute a minimum required LO photon number $n_{\mathrm{LO}}$, and the equivalent photodiode added noise $\sigma_{e}$ in electrons at this LO photon number. If the photodiode added noise is less than 730 or 8250 [18, 19], we give the required LO photon number for which the photodiode added noise is consistent with r. Using an LO with this average photon number guarantees the calculated bounds on infidelity.

$\bar{n}$	$\;\textrm{db}$	$\sigma_{\mathrm{noise}}$	$\sigma_{0}$	$\epsilon_{\mathrm{ec}}$	$\epsilon_{m}$	r	$n_{\mathrm{LO}}$	$\sigma_{e}$	$n_{\mathrm{LO}}$ at $\sigma_{e} = 730$	$n_{\mathrm{LO}}$ at $\sigma_{e} = 8250$
4.8	9.8	0.100	0.050	0.060	0.020	0.071	$6.0\times 10^{5}$	54.7	$1.1\times 10^{8}$	$1.4\times 10^{10}$
7.6	11.8	0.100	0.050	0.015	0.005	0.071	$2.8\times 10^{6}$	118.0	$1.1\times 10^{8}$	$1.4\times 10^{10}$
12.0	13.8	0.100	0.050	0.0020	0.0005	0.071	$3.4\times 10^{7}$	412.4	$1.1\times 10^{8}$	$1.4\times 10^{10}$
12.0	13.8	0.180	0.090	0.0080	0.0020	0.127	$1.8\times 10^{6}$	172.0	$3.3\times 10^{7}$	$4.2\times 10^{9}$
12.0	13.8	0.180	0.045	0.0080	0.0020	0.229	$4.9\times 10^{5}$	159.8	$1.0\times 10^{7}$	$1.3\times 10^{9}$
12.0	13.8	0.150	0.075	0.0050	0.0050	0.106	$1.1\times 10^{6}$	113.4	$4.7\times 10^{7}$	$6.0\times 10^{9}$
15.4	14.9	0.140	0.050	0.0008	0.0002	0.156	$1.4\times 10^{7}$	579.6	$2.2\times 10^{7}$	$2.8\times 10^{9}$

## 8. Discussion

The results in this paper are predicated on having detectors that have a linear response to the number of photons in each mode in a mode basis, and the examples in section 7 assume detector noise is additive Gaussian. Other forms of additive noise are in principle accounted for by our measurement models. Non-additive noise or detector non-linearities change the output-subtraction model and additional research is required to determine their effects.

Another common detector imperfection includes mode-dependent efficiencies. If the two detectors are perfectly matched with identical efficiencies in each mode, this effect is equivalent to mode-dependent losses of the signal. The measurement can therefore be treated as a measurement of a modified signal. If the efficiencies are known, it is possible to use such measurements to make inferences about the original signal, see [21] for an example with standard homodyne. Similarly, LO phase noise is equivalent to phase noise on the signal and the measurement can again be treated as a measurement of a modified signal.

Our analysis focuses on the information that can be obtained from the simple configuration involving a beamsplitter and two detectors whose output is subtracted. In principle more information is available by treating the two detector outputs separately [10, 12]. Such a treatment is beneficial if the LO is weak such as in weak-field homodyne [22, 23], and may be necessary if the detectors are imperfectly matched or have significant non-linearities. Future work is required to investigate the benefits of separate treatments of the detector outputs in these regimes.

## Data Availability

All data that support the findings of this study are included within the article (and any supplementary files). Data and code for figures available at https://doi.org/10.1088/1751-8121/aea45c/data1.

## Appendix A Functional analysis

Hilbert spaces in this paper are assumed to be separable. We make frequent use of projection-valued measures and their representations. For definitions of projection-valued measures and their use in the spectral theorem and the Borel functional calculus for self-adjoint operators, see [24], Chpt. VIII. In this paper, the domains of defined projection-valued measures are assumed to be standard Borel spaces. Functions that are introduced or defined are required to be Borel measurable. We do not explicitly specify the required Borel measurability in our results. All measures introduced are σ-finite by assumption. For the relevant measure theoretic concepts see real analysis textbooks such as [25]. When we write expressions such as $\mathcal{H} = \mathcal{H}_{A}\otimes \mathcal{H}_{B}$ or $\mathcal{H} = \bigoplus_{k\in J}\mathcal{H}_{k}$, the equality symbols imply an implicit isometry that we do not need to refer to explicitly in calculations.

We symbolize projection-valued measures by the expression dQ(x) for x in a standard Borel space $\mathcal{B}$. The projection determined by the projection-valued measure dQ(x) for the Borel subset X of $\mathcal{B}$ is $\int_{x\in X}\mathrm d Q(x)$. For every state $\ket{\psi}$, $\bra{\psi}\mathrm d Q(x)\ket{\psi}$ is a bounded measure on $\mathcal{B}$. The measure is defined by $\int_{x\in X}\bra{\psi}\mathrm d Q(x)\ket{\psi} = \bra{\psi}\int_{x\in X}\mathrm dQ(x) \ket{\psi}$. According to the spectral theorem in projection-valued measure form, for every self-adjoint operator $\hat{A}$, its projection-valued measure dA(x) on the reals satisfies that if h(x) is a Borel function on the reals, then $h(\hat{A}) = \int_{x\in rls}h(x)\mathrm dA(x)$ is a self-adjoint operator whose domain consists of the states $\ket{\psi}$ for which the measure $|h(x)|^{2}\bra{\psi}d A(x)\ket{\psi}$ is bounded. For h(x)=x, $h(\hat{A}) = \hat{A}$.

Every projection-valued measure dQ(x) on a standard Borel space $\mathcal{Y}$ has a direct-integral representation. The direct-integral representation simplifies computations and helps to define conditional unitary operations. The theory of direct integrals is described in many functional analysis books. For example, see [26] chapter 14 and [27] Chpt. IV. Let $\mathcal{H}_{k}$ be the Hilbert space of dimension k for $k = 1,\ldots,\infty$, where $\mathcal{H}_{\infty}$ is the infinite-dimensional separable Hilbert space. According to the direct-integral representation, there is a σ-finite measure $\mathrm d\mu(x)$ on $\mathcal{Y}$ and a measurable partition $\mathcal{Y} = \bigcup_{k = 1}^{\infty}\mathcal{Y}_{k}$ such that the Hilbert space $\mathcal{H}$ of dQ(x) can be decomposed as $\mathcal{H} = \bigoplus_{k = 1}^{\infty}L^{2}(\mathcal{Y}_{k},\mathrm d\mu(x)|_{\mathcal{Y}_{k}},\mathcal{H}_{k})$, and the projector onto $L^{2}(\mathcal{Y}_{k},\mathrm d\mu(x)|_{\mathcal{Y}_{k}},\mathcal{H}_{k})$ in this decomposition is $\int_{x\in\mathcal{Y}_{k}}\mathrm dQ(x)$. We write $\mathcal{H}(x) = \mathcal{H}_{k}$ for $x\in\mathcal{Y}_{k}$. The Hilbert space $L^{2}(\mathcal{Y}_{k},\mathrm d\mu(x)|_{\mathcal{Y}_{k}},\mathcal{H}_{k})$ is the Hilbert space of functions from $\mathcal{Y}_{k}$ to $\mathcal{H}_{k}$ that are $\mathcal{Y}_{k}$-measurable and square integrable with respect to $\mathrm d\mu(x)$ restricted to $\mathcal{Y}_{k}$. Such functions are unique up to null-sets of $\mathrm d\mu(x)$. This Hilbert space is naturally isomorphic to $L^{2}(\mathcal{Y}_{k},\mathrm d\mu(x)|_{\mathcal{Y}_{k}})\otimes \mathcal{H}_{k}$, where $L^{2}(\mathcal{Y}_{k},\mathrm d\mu(x)|_{\mathcal{Y}_{k}})$ is the Hilbert space of $\mathcal{Y}_{k}$-measurable functions from $\mathcal{Y}_{k}$ to the complex numbers. $L^{2}(\mathcal{Y}_{k},\mathrm d\mu(x)|_{\mathcal{Y}_{k}})$ has the natural projection-valued measure $\mathrm d\Pi_{k}(x)$ defined by $\int_{X}\mathrm d\Pi_{k}(x)\ket{g(\cdot)} = \ket{\chi_{X}(\cdot)g(\cdot)}$, where $\chi_{X}(x)$ is the characteristic function of the set X. The projection valued measure dQ(x) restricted to $\mathcal{Y}_{k}$ can then be written as $\mathrm d\Pi_{k}(x)\otimes\unicode{x1D7D9}$ in the factorization $L^{2}(\mathcal{Y}_{k},\mathrm d\mu(x)|_{\mathcal{Y}_{k}})\otimes \mathcal{H}_{k}$. We formally write $\mathcal{H} = \int_{x\in\mathcal{Y}}^{\oplus}\mathcal{H}(x)$, where $\mathcal{H}(x) = \mathcal{H}_{k}$ for $x\in\mathcal{Y}_{k}$. Here the ⊕ in the superscript specifies that this is a direct integral, not a conventional integral. Accordingly, states $\ket{\psi}$ of $\mathcal{H}$ can be written formally in the form $\ket{\psi} = \int_{x\in\mathcal{Y}}^{\oplus} \mathrm d\mu(x) \ket{\psi(x)}$, where for $x\in\mathcal{Y}_{k}$, $\ket{\psi(x)}\in\mathcal{H}_{k}$. Restricted to $\mathcal{Y}_{k}$, $\ket{\psi(x)}$ is a measurable function from $\mathcal{Y}_{k}$ to $\mathcal{H}_{k}$ that is square-integrable on $\mathcal{Y}_{k}$ with respect to $\mathrm d\mu(x)|_{\mathcal{Y}_{k}}$. This means that for all $\ket{\phi}$ in $\mathcal{H}_{k}$, $\braket{\phi}{\psi(x)}$ is measurable on $\mathcal{Y}_{k}$ and $\sum_{k = 1}^{\infty}\int_{x\in\mathcal{Y}_{k}} \mathrm d\mu(x)\braket{\psi(x)} < \infty$. In the complete direct integral expression $\ket{\psi} = \int_{x\in\mathcal{Y}}^{\oplus} \mathrm d\mu(x) \ket{\psi(x)}$, the superscript ⊕ on the integral indicates that this is to be interpreted as a vector-valued measure. Informally, the expression $\mathrm d\mu(x)$ in the integral is a kind of square-root measure. It is determined up to equivalence of measures with respect to absolute continuity, and change of measure is accompanied by the square root of the Radon-Nikodym derivative. Decomposable operators are bounded operators $\hat{A}$ on $\mathcal{H}$ that can be written in the form $\hat{A} = \int_{x\in\mathcal{Y}} \mathrm dQ(x) \hat{A}(x)$, where for $x\in\mathcal{Y}_{k}$, $\hat{A}(x)$ is an operator on $\mathcal{H}_{k}$, and the function $\hat{A}(x)$ satisfies certain measurability conditions for $\hat{A}$ to be well-defined. Intuitively, $\hat{A}$ is an operator that acts conditionally on $x\in\mathcal{Y}$. Decomposable operators are characterized as operators that are in the commutant of the von-Neumann algebra of operators of the form $\int_{x\in\mathcal{Y}}\mathrm dQ(x) h(x)$ for bounded Borel functions h(x).

If $\mathcal{H}$ is a tensor factor of a larger Hilbert space $\mathcal{H}_{T} = \mathcal{H}_{A}\otimes\mathcal{H}$, then the direct integral tensors with $\mathcal{H}_{A}$ as

\begin{align*} \mathcal{H}_{T}&amp; = \bigoplus_{k = 1}^{\infty}\mathcal{H}_{A}\otimes L^{2}(\mathcal{Y}_{k},\mathrm d\mu(k)|_{\mathcal{Y}_{k}},\mathcal{H}_{k}) \nonumber\\ &amp; = \bigoplus_{k = 1}^{\infty}L^{2}(\mathcal{Y}_{k},\mathrm d\mu(k)|_{\mathcal{Y}_{k}},\mathcal{H}_{k}\otimes\mathcal{H}_{A}).\end{align*}

We can treat dQ(x) as a projection valued measure of $\mathcal{H}_{T}$ in the usual way and write product states $\ket{\phi}_{A}\otimes\ket{\psi}$ of $\mathcal{H}_{T}$ in the form $\int_{x\in\mathcal{Y}}^{\oplus}\mathrm d\mu(x)\ket{\phi}_{A}\otimes \ket{\psi(x)}$.

We make use of bounded complex measures on $\mathbb{R}$. The definitions and properties of signed and complex measures can be found in standard books on real analysis such as [25], chapter 11 Section 5 and chapter 11 Section 6, Prob. 36. For our purposes, a bounded complex measure $\mathrm d\tilde\mu(\kappa)$ on the reals is defined by $\mathrm d\tilde\mu(\kappa) = \mathrm e^{\mathrm i\theta(\kappa)}\mathrm d\mu(\kappa)$, where $\theta(\kappa)$ is a measurable real function and $\mathrm d\mu(\kappa)$is a bounded positive measure. When introducing bounded complex measures, we denote the complex measure with a tilde, such as $\mathrm d\tilde\mu(\kappa)$ and denote the associated positive measure without the tilde, such as $\mathrm d\mu(\kappa)$. The measure $\mathrm d\mu(\kappa)$ can be thought of as the absolute value of the measure $\mathrm d\tilde\mu(\kappa)$.

For a function $\tilde f(\kappa)$ on the reals in a function space for which Fourier transforms can be defined, we use the convention that its Fourier transform is $f(x) = \int_{\kappa\in\mathbb{R}}\mathrm e^{\mathrm i\kappa x}\tilde f(\kappa)\mathrm d\kappa$. The inverse Fourier transform of f(x) is $\tilde f(\kappa) = \frac{1}{2\pi}\int_{x\in\mathbb{R}}\mathrm e^{-\mathrm i\kappa x}f(x)\mathrm dx$. With this definition, the convolution $(\tilde f *\tilde g)(\kappa)$ of $\tilde f(\kappa)$ and $\tilde g(\kappa)$ has Fourier transform f(x)g(x). The $L_{2}$-norm of f(x) is $\|f(x)\|_{2} = \sqrt{2\pi} \| \tilde f(\kappa)\|_{2}$.

The Fourier transform $f(x) = \int_{\kappa\in\mathbb{R}} \mathrm e^{\mathrm i\kappa x} \mathrm d\tilde\mu(\kappa)$ of a bounded complex measure is a continuous, bounded function. Functions such as $f(x) = \mathrm e^{\mathrm ix\kappa}$ and functions with integrable Fourier transforms are the Fourier transforms of bounded complex measures.

We claimed that for observables with continuous spectrum, exact measurements do not exist. For the measurement of $\hat{q}_{0}$, the final state of the system and apparatus after a putative exact measurement would be

\begin{align*} \ket{\phi_{0}} = \int_{x} \mathrm d\Pi_{0}(x)\ket{\psi}\otimes\ket{x}_{M},\end{align*}

where $\ket{\psi}$ is the state of the measured system and $\ket{x}_M$ is the state of the apparatus recording the measurement result, x. Consider the case where the system and apparatus Hilbert spaces are $L^{2}(\mathbb{R})$. Then the final state ought to be in $L^{2}(\mathbb{R}^{2})$. However, the ideal measurement ought to result in a state that is supported on the diagonal $\{(x,x): x\in\mathbb{R}\}$ of $\mathbb{R}^{2}$. But since the diagonal has measure zero, no such state exists. Similar problems occur when attempting to define the classical-quantum postmeasurement state. These problems are associated with the fact that for a given value of x, $\Pi_{0}(x)$ cannot be interpreted as a bounded or unbounded operator. The expression $\Pi_{0}(x)\ket{\psi}$ ought to be a state whose wavefunction is supported on $\{x\}$ but no such state exists in the Hilbert space.

## Appendix B Often used relationships

We use the following basic inequalities:

\begin{align*} \require{physics} \Re\qty(\mathrm e^{\mathrm i\theta}) &amp; = \cos(\theta) {\unicode{x2A7E}} 1- {\theta^{2}}/{2},\end{align*}

\begin{align*} \sqrt{|x|+|y|} &amp;{\unicode{x2A7D}} \sqrt{|x|} + \sqrt{|y|},\end{align*}

\begin{align*} \require{physics} (x+y)^{2} &amp;{\unicode{x2A7D}} 2(x^{2}+y^{2}),\end{align*}

where in the last inequality, x and y are real.

For bounded operators $\hat{B}$ we define $\Re\qty(\hat{B}) = \hat{B}+\hat{B}^{\dagger}$. For a self-adjoint operator $\hat{A}$, theinequality equation (B1) implies the operator inequality

\begin{align*} \require{physics} \Re\qty(\mathrm e^{\mathrm i\hat{A}}) {\unicode{x2A7E}} 1-\hat{A}^{2}/2,\end{align*}

where the right-hand side has expectation −∞ for states not in the domain of $\hat{A}$.

For self-adjoint operators $\hat{A}$ and $\hat{B}$ with $\hat{B}$ bounded, the operator $\hat{A}+\hat{B}$ is self-adjoint with the same domain as $\hat{A}$. This is a consequence of the Kato-Rellich theorem, see [28] Thm. X.12. For a state $\ket{\psi}$ in the domain of $\hat{A}$, we have

\begin{align*}|\langle \hat{A}\rangle - \langle \hat{B}\rangle|^{2} &amp; = |\bra{\psi} \hat{A}-\hat{B}\ket{\psi}|^{2} \nonumber\\ &amp; = \bra{\psi} (\hat{A}-\hat{B})^{\dagger} \ketbra{\psi} (\hat{A}-\hat{B})\ket{\psi} \nonumber\\ &amp;{\unicode{x2A7D}} \bra{\psi} (\hat{A}-\hat{B})^{\dagger}(\hat{A}-\hat{B})\ket{\psi} \nonumber\\ &amp; = |(\hat{A}-\hat{B})\ket{\psi}|^{2}.\end{align*}

Let $\hat{p}$ be a normalized quadrature of a mode. Let $\hat{q}$ be its normalized conjugate quadrature in the mode so that $[\hat{q},\hat{p}] = 2i$ with our normalization conventions. Then

\begin{align*} \hat{p}^{2} {\unicode{x2A7D}} \hat{p}^{2}+\hat{q}^{2} = 4(\hat{n}+1/2).\end{align*}

We take advantage of inequalities relating fidelity to overlaps and distances. If $\Re(\braket{\phi_{1}}{\phi_{2}}) {\unicode{x2A7E}} 1-\epsilon$, then

\begin{align*}F(\ketbra{\phi_{1}},\ketbra{\phi_{2}}) &amp; = |\braket{\phi_{1}}{\phi_{2}}|^{2} \nonumber\\ &amp; = \Re(\braket{\phi_{1}}{\phi_{2}})^{2} + \Im(\braket{\phi_{1}}{\phi_{2}})^{2} \nonumber\\ &amp; {\unicode{x2A7E}} \Re(\braket{\phi_{1}}{\phi_{2}})^{2} \nonumber\\ &amp; = (1-\epsilon)^{2} \nonumber\\ &amp;{\unicode{x2A7E}} 1-2\epsilon.\end{align*}

If $|\ket{\phi_{1}}-\ket{\phi_{2}}|^{2}{\unicode{x2A7D}} \epsilon$, then from $|\ket{\phi_{1}}-\ket{\phi_{2}}|^{2} = 2-2\Re(\braket{\phi_{1}}{\phi_{2}})$ we obtain $\Re(\braket{\phi_{1}}{\phi_{2}}) {\unicode{x2A7E}} 1-\epsilon/2$ and from equation (B7), $F(\ketbra{\phi_{1}},\ketbra{\phi_{2}}) {\unicode{x2A7E}} 1-\epsilon$. Conversely, suppose that $F(\ketbra{\phi_{1}},\ketbra{\phi_{2}}) {\unicode{x2A7E}} 1-\epsilon$. There exists a phase factor $\mathrm e^{\mathrm i\phi}$ such that $\bra{\phi_{1}}\mathrm e^{\mathrm i\phi}\ket{\phi_{2}}$ is real and positive and $|\ket{\phi_{1}}-\mathrm e^{\mathrm i\phi}\ket{\phi_{2}}|^{2} = 2-2|\braket{\phi_{1}}{\phi_{2}}| = 2\qty(1-\sqrt{F(\ketbra{\phi_{1}},\ketbra{\phi_{2}})}){\unicode{x2A7D}} 2\qty(1-\sqrt{1-\epsilon}){\unicode{x2A7D}} 2\epsilon$.

## Appendix C Bounds on the distance between states coupled with $\hat{q}_{\delta}$ and $\hat{q}$

For convenience, we omit the tensor product symbol ⊗ and use the convention that operators and states labeled with M belong to the apparatus system. We wish to determine a bound on

\begin{align*} |\mathrm e^{-\mathrm i\hat{q}_{\delta}\hat{s}_{M}}\ket{\psi} - \mathrm e^{-\mathrm i\theta}\mathrm e^{-\mathrm i\hat{q}\hat{s}_{M}}\ket{\psi}|^{2} = 2-2\Re(\mathrm e^{-\mathrm i\theta}\bra{\psi}\mathrm e^{\mathrm i\hat{q}_{\delta}\hat{s}_{M}} \mathrm e^{-\mathrm i\hat{q}\hat{s}_{M}}\ket{\psi})\end{align*}

for states $\ket{\psi}$ that are vacuum on the LO modes, where we can minimize the bound over real θ since we do not distinguish states with different global phases. Because the action on M is diagonal with respect to the projection valued measure $\mathrm d\Pi(s)_{M}$ of $\hat{s}_{M}$, we can write

\begin{align*} \require{physics} \mathrm e^{\mathrm i\hat{q}_{\delta}\hat{s}_{M}}\mathrm e^{-\mathrm i\hat{q}\hat{s}_{M}} &amp; = \int_{s}\mathrm d\Pi(s)_{M} \qty(\mathrm e^{\mathrm i\hat{q}_{\delta}\hat{s}_{M}}\mathrm e^{-\mathrm i\hat{q}\hat{s}_{M}}) \nonumber\\ &amp; = \int_{s}\mathrm d\Pi(s)_{M}\qty(\mathrm e^{\mathrm i\hat{q}_{\delta}s}\mathrm e^{-\mathrm i\hat{q}s})\end{align*}

and substitute in equation (C1). For an upper bound on the left-hand side of this identity, it suffices to determine a lower bound on $\Re\qty(\bra{\psi}\mathrm e^{\mathrm i\hat{q}_{\delta}s}\mathrm e^{-\mathrm i\hat{q}s}\ket{\psi})$ as a function of s and integrate. For this purpose, we first express $\mathrm e^{\mathrm i\hat{q}_{\delta}s}\mathrm e^{-\mathrm i\hat{q}s}$ as a convenient conjugation by displacements. The terms for different mode pairs $a_{k}, b_{k}$ in $\hat{q}_{\delta}$ and $\hat{q}$ commute. In consideration of equation (7), the definition of δ and the relationship between $\boldsymbol{\alpha}$ and $\boldsymbol{\beta}$, we can write

\begin{align*} \mathrm e^{\mathrm i\hat{q}_{\delta}s}\mathrm e^{-\mathrm i\hat{q}s} = \prod_{k = 1}^{N}\mathrm e^{\mathrm i( \omega_{k} ( \hat{a}_{k}^{\dagger} \beta_{k} + \beta_{k}^{*} \hat{a}_{k} ) + \delta \omega_{k} ( \hat{a}_{k}^{\dagger} \hat{b}_{k} + \hat{b}_{k}^{\dagger} \hat{a}_{k} ))s} \mathrm e^{-\mathrm i( \omega_{k} ( \hat{a}_{k}^{\dagger} \beta_{k} + \beta_{k}^{*} \hat{a}_{k} ))s}.\end{align*}

For the next steps, fix $k\in\{1,\ldots,N\}$ and define

\begin{align*} \hat{q}_{a}&amp; = \hat{a}_{k}^{\dagger} \beta_{k} + \beta_{k}^{*} \hat{a}_{k}, \nonumber\\ \hat{q}_{b} &amp; = -\mathrm i(\hat{b}_{k}^{\dagger} \beta_{k} - \beta_{k}^{*} \hat{b}_{k}), \nonumber\\ \hat{B} &amp; = \hat{a}_{k}^{\dagger} \hat{b}_{k} + \hat{b}_{k}^{\dagger} \hat{a}_{k} .\end{align*}

Let $t = \omega_{k}s$ so that the mode k factor of equation (C3) can be written as

\begin{align*} \hat{F}_{k} = \mathrm e^{\mathrm it(\hat{q}_{a}+\delta \hat{B})}\mathrm e^{-\mathrm it\hat{q}_{a}}.\end{align*}

We have the following commutation relationships between $\hat{q}_{a}$, $\hat{q}_{b}$ and $\hat{B}$

\begin{align*} [\hat{q}_{a},\hat{q}_{b}] &amp; = 0 ,\nonumber\\ [\hat{q}_{a},\hat{B}] &amp; = -\mathrm i \hat{q}_{b} ,\nonumber\\ [\hat{q}_{b},\hat{B}] &amp; =\mathrm i\hat{q}_{a} ,\end{align*}

which implies that the three operators generate an affine extension of the circle group with $\hat{q}_{a}$ and $\hat{q}_{b}$ generating displacements and $\hat{B}$ generating rotations. Let $\vec{q} = (\hat{q}_{a},\hat{q}_{b})^\mathrm {T}$, and define the rotation matrix $R_{\phi} = \begin{pmatrix} \cos(\phi)&amp; -\sin(\phi)\\ \sin(\phi)&amp; \cos(\phi) \end{pmatrix} $ and the matrix $S = \begin{pmatrix} 0&amp;1\\-1&amp;0 \end{pmatrix} $.

The commutation relationships imply conjugation relationships

\begin{align*}\mathrm e^{-\mathrm i\hat{B}\phi}\vec{q}\mathrm e^{\mathrm i\hat{B}\phi} &amp; = R_{-\phi}\vec{q}, \nonumber\\ \mathrm e^{-\mathrm iu^\mathrm {T}\vec{q}}\hat{B}\mathrm e^{\mathrm i\vec{u}^\mathrm {T}\vec{q}} &amp; = \hat{B} - \vec{u}^\mathrm {T}S\vec{q}\end{align*}

for two-dimensional, real row vectors $\vec{u}$. Consequently

\begin{align*} \mathrm e^{-\mathrm i\vec{u}^\mathrm {T}\vec{q}} \mathrm e^{\mathrm i\phi \hat{B}} \mathrm e^{\mathrm i\vec{u}^\mathrm {T}\vec{q}} &amp; = \mathrm e^{\mathrm i\phi (B- \vec{u}^\mathrm {T}S\vec{q}))}\end{align*}

\begin{align*} &amp;= \mathrm e^{\mathrm i\phi \hat{B}}\mathrm e^{\mathrm i\vec{u}^\mathrm {T}(1- R_{-\phi})\vec{q}},\end{align*}

where the second line is obtained from the left-hand side of the first by moving $\mathrm e^{\mathrm i\phi \hat{B}}$ to the left and merging the two commuting exponentials that result on the right. By solving for $\vec{u}^\mathrm {T}$ in the identity $\vec{v}^\mathrm {T} = \vec{u}^\mathrm {T}(1-R_{-\phi})$, we can rewrite the identity between the two operators on the right-hand sides as

\begin{align*} \mathrm e^{\mathrm i\phi \hat{B}} \mathrm e^{\mathrm i \vec{v}^\mathrm {T}\vec{q}} = \mathrm e^{\mathrm i\phi (\hat{B} - \vec{v}^\mathrm {T}(1-R_{-\phi})^{-1}S\vec{q})},\end{align*}

provided that $R_{-\phi}\ne \unicode{x1D7D9}$. We modify $\hat{F}_{k}$ by replacing $\hat{q}_{a}$ with $\vec{w}^\mathrm {T}\vec{q}$ and compute

\begin{align*} \mathrm e^{\mathrm it( \vec{w}^\mathrm {T}\vec{q}+\delta\hat{B})}\mathrm e^{-\mathrm it \vec{w}^\mathrm {T}\vec{q}} = \mathrm e^{-\mathrm i \vec{w}^\mathrm {T}S\vec{q}/\delta}\mathrm e^{\mathrm it\delta\hat{B}}\mathrm e^{-\mathrm it\vec{w}^\mathrm {T}\vec{q}}\mathrm e^{\mathrm i\vec{w}^\mathrm {T}S\vec{q}/\delta}.\end{align*}

For this computation, we applied equation (C8) to the first factor, where we replaced $\vec{u}^\mathrm {T}$ by $\vec{w}^\mathrm {T}S/\delta$ and ϕ by δ, and exchanged commuting terms. Let $\phi = [t\delta]_{\pi} = ((t\delta+\pi)\mod(2\pi)) - \pi$ be the angle tδ expressed in the interval $[-\pi,\pi]$ modulo 2π. The term $\mathrm e^{\mathrm it\delta\hat{B}}$ is 2π periodic in tδ as an operator, so $\mathrm e^{\mathrm it\delta\hat{B}} = \mathrm e^{\mathrm i\phi\hat{B}}$. This is because $\hat{B}$ is the beam-splitter Hamiltonian and can be written as the difference of two number operators of orthogonal modes with annihilation operators $(\hat{a}_{k}\pm\hat{b}_{k})/\sqrt{2}$. Therefore

\begin{align*} \mathrm e^{\mathrm it( \vec{w}^\mathrm {T}\vec{q}+\delta\hat{B})}\mathrm e^{-\mathrm it \vec{w}^\mathrm {T}\vec{q}} &amp; = \mathrm e^{-\mathrm i \vec{w}^\mathrm {T}S\vec{q}/\delta}\mathrm e^{\mathrm i\phi\hat{B}}\mathrm e^{-\mathrm it\vec{w}^\mathrm {T}\vec{q}}\mathrm e^{\mathrm i\vec{w}^\mathrm {T}S\vec{q}/\delta} \nonumber\\ &amp; = \mathrm e^{-\mathrm i \vec{w}^\mathrm {T}S\vec{q}/\delta}\mathrm e^{\mathrm i\phi(\hat{B}+ t\vec{w}^\mathrm {T}(1-R_{-\phi})^{-1}S\hat{q})}\mathrm e^{\mathrm i\vec{w}^\mathrm {T}S\vec{q}/\delta} \nonumber\\ &amp; = \mathrm e^{-\mathrm i \vec{w}^\mathrm {T}S\vec{q}/\delta}\mathrm e^{\mathrm it\vec{w}^\mathrm {T}(1-R_{-\phi})^{-1}\vec{q}}\mathrm e^{\mathrm i\phi\hat{B}} \mathrm e^{-\mathrm it\vec{w}^\mathrm {T}(1-R_{-\phi})^{-1}\vec{q}}\mathrm e^{\mathrm i\vec{w}^\mathrm {T}S\vec{q}/\delta}.\end{align*}

To obtain the second line, we applied equation (C10) to the middle two terms. For the third line, we applied equation (C8) to the middle term. The matrix $(1-R_{-\phi})^{-1}$ evaluates to

\begin{align*} \begin{pmatrix} 1-\cos(\phi) &amp; -\sin(\phi)\\ \sin(\phi)&amp; 1-\cos(\phi) \end{pmatrix}^{-1} &amp; = \frac{1}{(1-\cos(\phi))^{2}+\sin(\phi)^{2}} \begin{pmatrix} 1-\cos(\phi) &amp; \sin(\phi)\\ -\sin(\phi)&amp;1-\cos(\phi) \end{pmatrix} \nonumber\\ &amp; = \frac{1}{2(1-\cos(\phi))} \begin{pmatrix} 1-\cos(\phi) &amp; \sin(\phi)\\ -\sin(\phi)&amp;1-\cos(\phi) \end{pmatrix} \nonumber\\ &amp; = \frac{1}{2} \begin{pmatrix} 1&amp; \sin(\phi)/(1-\cos(\phi))\\ -\sin(\phi)/(1-\cos(\phi)) &amp;1 \end{pmatrix} \nonumber\\ &amp; = \frac{1}{2} \begin{pmatrix} 1&amp; \cot(\phi/2)\\ -\cot(\phi/2) &amp;1 \end{pmatrix},\end{align*}

where we used the trigonometric identities $\sin(\phi) = 2\sin(\phi/2)\cos(\phi/2)$ and $1-\cos(\phi) = 2\sin(\phi/2)^{2}$ for the last line. We substitute $\vec{w} = (1,0)$ in equation (C12) and write

\begin{align*} \require{physics} \mathrm e^{\mathrm it( \hat{q}_{a}+\delta\hat{B})}\mathrm e^{-\mathrm it \hat{q}_{a}} &amp; = \mathrm e^{-\mathrm i (\hat{q}_{b}/\delta-t(\hat{q}_{a}+\cot(\phi/2)\hat{q}_{b})/2)}\mathrm e^{\mathrm i\phi\hat{B}} \mathrm e^{\mathrm i(\hat{q}_{b}/\delta-t(\hat{q}_{a}+\cot(\phi/2)\hat{q}_{b})/2)} \nonumber\\ &amp; = \mathrm e^{\mathrm i t\qty(\frac{1}{2}\hat{q}_{a} - \qty(\frac{1}{\delta t}-\frac{\cot(\phi/2)}{2})\hat{q}_{b})} \mathrm e^{\mathrm i\phi\hat{B}} \mathrm e^{-\mathrm i t\qty(\frac{1}{2}\hat{q}_{a} - \qty(\frac{1}{\delta t}-\frac{\cot(\phi/2)}{2})\hat{q}_{b})} \nonumber\\ &amp; = \mathrm e^{\mathrm i t\qty(\frac{1}{2}\hat{q}_{a})} \mathrm e^{-\mathrm it\qty(\frac{1}{\delta t}-\frac{\cot(\phi/2)}{2})\hat{q}_{b}} \mathrm e^{\mathrm i\phi\hat{B}} \mathrm e^{\mathrm it\qty(\frac{1}{\delta t}-\frac{\cot(\phi/2)}{2})\hat{q}_{b}} \mathrm e^{-\mathrm i t\qty(\frac{1}{2}\hat{q}_{a})} \nonumber\\ &amp; = \mathrm e^{\mathrm i t\qty(\frac{1}{2}\hat{q}_{a})} \mathrm e^{\mathrm i\qty(\phi\hat{B}+t\qty(\frac{\phi}{t\delta}-\frac{\phi}{2}\cot(\phi/2))\hat{q}_{a})} \mathrm e^{-\mathrm i t\qty(\frac{1}{2}\hat{q}_{a})}.\end{align*}

Before we continue, we evaluate $g(\phi) = (\phi/2) \cot(\phi/2)$ for $\phi\in [-\pi,\pi]$. From the double angle formula for the cotangent, $(\phi/2)\cot(\phi/2) = (\phi/4)\cot(\phi/4) -(\phi/4) \tan(\phi/4)$. Recursively applying the double angle formula to the cotangent on the right-hand side gives

\begin{align*} g(\phi) = (\phi/2^{k})\cot(\phi/2^{k}) - \sum_{j = 2}^{k}(\phi/2^{j})\tan(\phi/2^{j}).\end{align*}

The limit as y goes to zero of ycot⁡(y) is 1, so letting $k\rightarrow\infty$ we get

\begin{align*} g(\phi) = 1-\phi \sum_{j = 2}^{\infty}\tan(\phi/2^{j})/2^{j}.\end{align*}

For $z\in[0,\pi/4]$, we have $z{\unicode{x2A7D}} \tan(z) {\unicode{x2A7D}} (4/\pi)z$. Therefore, for $\phi\in [0,\pi]$

\begin{align*} \frac{1}{12} \phi = \sum_{j = 2}^{\infty}\phi \,2^{-2j} {\unicode{x2A7D}} \sum_{j = 2}^{\infty}\tan(\phi/2^{j})/2^{j} {\unicode{x2A7D}} \frac{4}{\pi}\phi\sum_{j = 2}^{\infty}2^{-2j} = \frac{1}{3\pi} \phi {\unicode{x2A7D}} \frac{1}{9}\phi.\end{align*}

Since g(ϕ) is symmetric in ϕ, we deduce that for $\phi\in[-\pi,\pi]$,

\begin{align*} \require{physics} g(\phi) \in 1-\phi^{2}\qty[\frac{1}{12},\frac{1}{3\pi}] \subseteq 1-\phi^{2}\qty[\frac{1}{12},\frac{1}{9}].\end{align*}

We return to equation (C3). For this purpose we make the mode index explicit by writing $\hat{q}_{a,k}$, $\hat{q}_{b,k}$, $\hat{B}_{k}$, $\phi_{k}$ for $\hat{q}_{a}$, $\hat{q}_{b}$, $\hat{B}$, ϕ defined above. We also write $\hat{q}_{k} = \omega_{k}\hat{q}_{a,k}$ and substitute $\omega_{k}s$ for t. Since $\alpha_{k} = i\omega_{k}\beta_{k}$ we have $\sum_{k}\omega_{k}\hat{q}_{a,k} = \hat{q}$. The quadratures $\hat{q}_{k}$ have normalization $|\alpha_{k}|^{2}$. After applying equation (C14) to each mode, equation (C3) becomes

\begin{align*} \mathrm e^{\mathrm i\hat{q}_{\delta}s}\mathrm e^{-\mathrm i\hat{q}s} = \mathrm e^{\mathrm is\hat{q}/2} \mathrm e^{\mathrm i\sum_{k}\qty( \phi_{k}\hat{B}_{k} +s\qty(\frac{\phi_{k}}{\omega_{k}s\delta} - \frac{\phi_{k}}{2}\cot(\phi_{k}/2))\hat{q}_{k} )} \mathrm e^{-\mathrm is\hat{q}/2}.\end{align*}

This is of the form $\hat{U} \mathrm e^{\mathrm i\hat{H}}\hat{U}^{\dagger}$ for the unitary $\hat{U} = \mathrm e^{\mathrm is\hat{q}/2}$ and the self-adjoint operator

\begin{align*} \hat{H} = \sum_{k} \phi_{k}\hat{B}_{k} + s u(\delta\,s)\hat{u}_{\delta\,s},\end{align*}

where $\hat{u}_{\delta\,s}$ is a normalized quadrature that commutes with the $\hat{q}_{k}$. The quadrature $\hat{u}_{\delta\,s}$ is given by

\begin{align*} \require{physics} \hat{u}_{\delta\,s} = \frac{1}{u(\delta\,s)}\sum_{k}\qty(\frac{\phi_{k}}{\omega_{k}\delta\,s} - \frac{\phi_{k}}{2}\cot(\phi_{k}/2))\hat{q}_{k}\end{align*}

with $u(\delta\,s){\unicode{x2A7E}} 0$ defined by

\begin{align*} \require{physics} u(\delta\,s)^{2} = \sum_{k}|\alpha_{k}|^{2}\qty(\frac{\phi_{k}}{\omega_{k}\delta\,s} - \frac{\phi_{k}}{2}\cot(\phi_{k}/2))^{2}.\end{align*}

For the case where the $\omega_{k}$ are identical, $\hat{u}_{\delta\,s} = \hat{q}$.

We now have

\begin{align*} \left|{\mathrm e^{-\mathrm{i}\hat{q}_{\delta}s}\ket{\psi} - \mathrm e^{-\mathrm i\theta}\mathrm e^{-\mathrm i\hat{q}s}\ket{\psi}}\right|^{2} = \bra{\psi}\qty({2-2\Re\qty(\mathrm e^{\mathrm is\hat{q}/2}\mathrm e^{\mathrm i(\hat{H}-\theta)}\mathrm e^{-\mathrm is\hat{q}/2})}) \ket{\psi}.\end{align*}

By applying equation (B4) we can bound

\begin{align*} \require{physics} 2-2\Re\qty(\hat{U} \mathrm e^{\mathrm i\hat{H}-\theta}\hat{U}^{\dagger}) {\unicode{x2A7D}} \hat{U} (\hat{H}-\theta)^{2}\hat{U}^{\dagger}.\end{align*}

For states $\ket{\psi}$ that are vacuum on the LO modes, $\ket{\psi^{^{\prime}}} = U\ket{\psi}$ is also vacuum on the LO modes. When contracting the operator $\hat{H}^{2}$ with vacuum on the LO modes, terms that are linear in each LO mode’s annihilation and creation operators are eliminated. The surviving terms are those that involve no operators on the LO modes or those that act as $\hat{b}_{k}\hat{b}_{k}^{\dagger}$ on the LO modes. Therefore

\begin{align*} &amp; \left|\mathrm e^{-\mathrm i\hat q_{\delta}s}\ket{\psi} - \mathrm e^{-\mathrm i\theta}\mathrm e^{-\mathrm i\hat{q}s}\ket{\psi}\right|^{2} \kern-2in \nonumber\\ &amp;\quad {\unicode{x2A7D}} \bra{\psi}\mathrm e^{\mathrm is\hat{q}/2} \qty(\sum_{k}\phi_{k}^{2}\hat{a}_{k}^{\dagger}\hat{a}_{k} +\qty(su(\delta\,s)\hat{u}_{\delta\,s}-\theta)^{2})\mathrm e^{-\mathrm is\hat{q}/2}\ket{\psi} \nonumber\\ &amp;\quad = \sum_{k}\phi_{k}^{2} \bra{\psi}\mathrm e^{\mathrm is\hat{q}_{k}/2} \hat{a}_{k}^{\dagger}\hat{a}_{k} \mathrm e^{-\mathrm is\hat{q}_{k}/2}\ket{\psi} +\bra{\psi}\qty(s u(\delta\,s)\hat{u}_{\delta\,s}-\theta)^{2}\ket{\psi} \nonumber\\ &amp; \quad= \sum_{k}\phi_{k}^{2} \bra{\psi} \qty(\hat{a}_{k}^{\dagger}-s\alpha_{k}^{*}/2) \qty(\vphantom{\hat{a}_{k}^{\dagger}}\hat{a}_{k}-s\alpha_{k}/2) \ket{\psi} +\bra{\psi}\qty(s u(\delta\,s)\hat{u}_{\delta\,s}-\theta)^{2}\ket{\psi} \nonumber\\ &amp; \quad {\unicode{x2A7D}} \sum_{k}\phi_{k}^{2} \qty(2\bra{\psi} \hat{n}_{k} \ket{\psi}+s^{2}|\alpha_{k}|^{2}/2) +\bra{\psi}\qty(s u(\delta\,s)\hat{u}_{\delta\,s}-\theta)^{2}\ket{\psi}.\end{align*}

For the last line, we applied the inequality $\qty(\hat{A}-x)^{\dagger} \qty(\hat{A}-x) {\unicode{x2A7D}} 2\hat{A}^{\dagger}\hat{A}+2|x|^{2}$. To see this, for states $\ket{\phi}$ in the domain of $\hat{A}$ we have

\begin{align*} \require{physics} \bra{\phi}\qty(\hat{A}-x)^{\dagger} \qty(\hat{A}-x)\ket{\phi} &amp;{\unicode{x2A7D}} \bra{\phi}\qty(\hat{A}-x)^{\dagger} \qty(\hat{A}-x)\ket{\phi} + \bra{\phi}\qty(\hat{A}+x)^{\dagger} \qty(\hat{A}+x)\ket{\phi} \nonumber\\ &amp; = 2\bra{\phi}\hat{A}^{\dagger}\hat{A}\ket{\phi} + 2|x|^{2}\braket{\phi} = \bra{\phi}\qty( 2\hat{A}^{\dagger}\hat{A}+2|x|^{2})\ket{\phi}.\end{align*}

In equation (C25), for fixed s we can optimize the choice of θ by setting $\theta = \bra{\psi}su(\delta\,s)\hat{u}_{\delta\,s}\ket{\psi}$.

The bound of equation (C25) is our most specific bound on the difference between the states obtained after evolving according to $\hat{q}_{\delta}$ or $\hat{q}$ for the fixed eigenvalue s of the apparatus operator $\hat{s}_{M}$. In principle, it suffices to substitute the bound in equation (C1) after inserting the integral over the apparatus projection-valued measure $\mathrm d\Pi(s)_{M}$. Because of the unwieldy functional dependence on s in equation (C25), this does not result in readily interpretable bounds. In order to get more interpretable bounds, we derive conservative simplified bounds. For simplicity, and because s is to be replaced by $\hat{s}_{M}$, we set θ=0. We first treat the general case and then obtain specific bounds for the case of standard pulsed homodyne, in which case $\omega_{k} = \omega = 1$.

We begin with an upper bound on $\bra{\psi}u(\delta\,s)^{2}\hat{u}_{\delta s}^{2}\ket{\psi}$. The bound in equation (C25) is infinite if $\ket{\psi}$ is not in the domain of every $\hat{n}_{k}$ with non-zero $\phi_{k}$. We therefore assume that $\bra{\psi}\hat{n}_{k}\ket{\psi}$ is finite for every k. Equivalently, we assume that the total photon number expectation is finite. Define $h_{k}(\delta\,s) = \phi_{k}/(\omega_{k}\delta\,s) - (\phi_{k}/2)\cot(\phi_{k}/2)$. Let $\hat{n}_{\delta\,s}$ be the number operator for the mode associated with the normalized quadrature $\hat{u}_{\delta\,s}$. With our conventions for quadrature normalization, we have $\hat{u}_{\delta\,s}^{2}{\unicode{x2A7D}} 4(\hat{n}_{\delta\,s}+1/2)$, see equation (B6). An annihilation operator for the mode of $\hat{u}_{\delta\,s}$ is given by

\begin{align*} \hat{a}_{u} = \frac{1}{u(\delta\,s)}\sum_{k}\alpha_{k}^{*}h_{k}(\delta\,s) \hat{a}_{k},\end{align*}

where we overloaded the suffixes of a for convenience. This gives

\begin{align*} \bra{\psi}u(\delta\,s)^{2}\hat{n}_{u}\ket{\psi} &amp; = \left| u(\delta\,s)\hat{a}_{u}\ket{\psi}\right|^{2} \nonumber\\ &amp; = \left|\sum_{k}\alpha_{k}^{*}h_{k}(\delta\,s) \hat{a}_{k}\ket{\psi}\right|^{2}.\end{align*}

We need the following version of the Cauchy–Schwarz inequality. For coefficients $\gamma_{k}$, operators $\hat{A}_{k}$ and a state $\ket{\varphi}$ in the domain of every operator $\hat{A}_{k}$, we have

\begin{align*} \left|\sum_{k}\gamma_{k}\hat{A}_{k}\ket{\varphi}\right|^{2} {\unicode{x2A7D}} \sum_{k}|\gamma_{k}|^{2} \sum_{k}\left|\hat{A}_{k}\ket{\varphi}\right|^{2}.\end{align*}

To apply this inequality, we set $\gamma_{k} = \alpha_{k}^{*}\sqrt{h_{k}(\delta\,s)}$ and $\hat{A}_{k} = \sqrt{h_{k}(\delta\,s)}\hat{a}_{k}$. This gives

\begin{align*} \bra{\psi}u(\delta\,s)^{2}\hat{n}_{\delta\,s}\ket{\psi} {\unicode{x2A7D}} \qty(\sum_{k}|\alpha_{k}|^{2}|h_{k}(\delta\,s)|) \sum_{k}|h_{k}(\delta\,s)|\bra{\psi} \hat{n}_{k}\ket{\psi}.\end{align*}

Define $\bar h(\delta\,s) = \sum_{k}|\alpha_{k}|^{2}|h_{k}(\delta\,s)|$, $\overline{h^{2}}(\delta\,s) = \sum_{k}|\alpha_{k}|^{2}h_{k}(\delta\,s)^{2} = u(\delta\,s)^{2}$ and $\overline{\phi^{2}}(\delta\,s) = \sum_{k}|\alpha_{k}|^{2}\phi_{k}^{2}$, which are weighted averages of the $|h_{k}(\delta\,s)|$, $h_{k}(\delta\,s)^{2}$ and $\phi_{k}^{2}$. Substituting in equation (C25) with θ=0 gives

\begin{align*}\left|\mathrm e^{-\mathrm i\hat q_{\delta}s}\ket{\psi} - \mathrm e^{-\mathrm i\theta}\mathrm e^{-\mathrm i\hat{q}s}\ket{\psi}\right|^{2} \kern-1in &amp;\nonumber\\ &amp;{\unicode{x2A7D}} \sum_{k}\qty(2\phi_{k}^{2}+4 s^{2}|h_{k}(\delta\,s)| \bar h(\delta\,s) ) \bra{\psi}\hat{n}_{k}\ket{\psi} + s^{2}\qty(\overline{\phi^{2}}(\delta\,s)/2 + 2\overline{h^{2}}(\delta\,s)).\end{align*}

To bound the coefficients in equation (C31) with more simple expressions, we use the following inequalities:

\begin{align*} \require{physics} \phi_{k}^{2} &amp;{\unicode{x2A7D}} (\omega_{k} \delta\,s )^{2} , \; \phi_{k}^{2} {\unicode{x2A7D}} \pi^{2}, \nonumber\\[5pt] \left|\frac{\phi_{k}}{\omega_{k}\delta\,s}-1\right| &amp;{\unicode{x2A7D}}\frac{2}{\pi^{2}}(\omega_{k}\delta\,s)^{2} ,\; \left|\frac{\phi_{k}}{\omega_{k} \delta\,s}-1\right| {\unicode{x2A7D}} 2, \nonumber\\[5pt] \left|1-\frac{\phi_{k}}{2}\cot(\frac{\phi_{k}}{2})\right| &amp;{\unicode{x2A7D}} \frac{1}{3\pi}\phi_{k}^{2} {\unicode{x2A7D}} \frac{1}{3\pi} (\omega_{k}\delta\,s)^{2} ,\; \left|1-\frac{\phi_{k}}{2}\cot(\frac{\phi_{k}}{2})\right| {\unicode{x2A7D}} 1, \nonumber\\[5pt] |h_{k}(\delta\,s)| &amp;{\unicode{x2A7D}} \left|\frac{\phi_{k}}{\omega_{k}\delta\,s} -1\right| + \left|1 - \frac{\phi_{k}}{2}\cot(\frac{\phi_{k}}{2})\right| \nonumber\\[5pt] &amp;{\unicode{x2A7D}} \frac{1}{\pi^{2}}(2+\pi/3)(\omega_{k}\delta\,s)^{2} {\unicode{x2A7D}} \frac{1}{3}(\omega_{k}\delta\,s)^{2}, \nonumber\\ |h_{k}(\delta\,s)| &amp;{\unicode{x2A7D}} 3.\end{align*}

For the last inequality for $|h_{k}(\delta\,s)|$, we simplified the factor by applying $2+\pi/3{\unicode{x2A7D}} \pi$ and π>3. Define $\overline{\omega^{2}} = \sum_{k}|\alpha_{k}|^{2}\omega_{k}^{2}$. For substitution into equation (C31) we bound the terms according to

\begin{align*} \phi_{k}^{2} &amp; {\unicode{x2A7D}} (\delta\,s)^{2}\omega _{k}^{2}, \nonumber\\[5pt] |h_{k}(\delta\,s)|\bar h(\delta\,s) &amp;{\unicode{x2A7D}} 3 |h_{k}(\delta\,s)| {\unicode{x2A7D}} (\delta\,s)^{2}\omega_{k}^{2}, \nonumber\\[5pt] \overline{\phi^{2}}(\delta\,s) &amp;{\unicode{x2A7D}} (\delta\,s)^{2}\overline{\omega^{2}} \nonumber\\[5pt] \overline{h^{2}}(\delta\,s) &amp; = \sum_{k}|\alpha_{k}|^{2}h_{k}(\delta\,s)^{2} \nonumber\\[5pt] &amp;{\unicode{x2A7D}} 3\sum_{k}|\alpha_{k}|^{2}|h_{k}(\delta\,s)| \nonumber\\[5pt] &amp; = 3 \bar{h}(\delta\,s) {\unicode{x2A7D}} (\delta\,s)^{2} \overline{\omega^{2}}.\end{align*}

Define $\hat{\Omega} = \sum_{k}\omega_{k}^{2}\hat{n}_{k}$. We obtain

\begin{align*} \left|\mathrm e^{-\mathrm i\hat q_{\delta}s}\ket{\psi} - \mathrm e^{-\mathrm i\hat{q}s}\ket{\psi}\right|^{2} {\unicode{x2A7D}} (\delta\,s)^{2}\bra{\psi} \qty(2\qty(1+2s^{2})\hat{\Omega} + s^{2}(5/2)\overline{\omega^{2}}) \ket{\psi}\end{align*}

\begin{align*} {\unicode{x2A7D}} 4(\delta\,s)^{2}\bra{\psi} \qty(\qty(1+s^{2})\hat{\Omega} + s^{2}\overline{\omega^{2}}) \ket{\psi}.\end{align*}

The last inequality is conservative for the sake of simplicity but retains the same factor for the coefficient of $\delta^{2}s^{4}\hat{\Omega}$. The second bound in theorem 5.1 is obtained by moving the leading factor of $s^{2}$ into the bra-ket expression and substituting $\hat{s}_{M}$ for s.

The bound of equation (C34) is not optimized for the case of small $\delta\,s$ in that it conservatively estimates extra factors of order $(\delta\,s)^{2}$ for small $\delta\,s$. To obtain a bound that includes these extra factors we bound the coefficients of equation (C31) according to

\begin{align*} \bar h(\delta\,s) &amp; {\unicode{x2A7D}} (\delta\,s)^{2}\overline{\omega^{2}}/3 \nonumber\\ |h_{k}(\delta\,s)|\bar h(\delta\,s) &amp;{\unicode{x2A7D}} (\delta\,s)^{4}\omega_{k}^{2}\overline{\omega ^{2}}/9 \nonumber\\ \overline{h^{2}}(\delta\,s) &amp;{\unicode{x2A7D}} (\delta\,s)^{4}\sum_{k}|\alpha_{k}|^{2}\omega_{k}^{4}/9 = (\delta\,s)^{4}\overline{\omega^{4}}/9,\end{align*}

where we introduced $\overline{\omega^{4}} = \sum_{k}|\alpha_{k}|^{2}\omega_{k}^{4}$. We get

\begin{align*} &amp;\left|\mathrm e^{-\mathrm i\hat q_{\delta}s}\ket{\psi} - \mathrm e^{-\mathrm i\hat{q}s}\ket{\psi}\right|^{2} \kern-1.5in \nonumber\\ &amp;\quad {\unicode{x2A7D}} (\delta\,s)^{2}\bra{\psi} \qty(2\qty(1+(2/9)s^{2}(\delta\,s)^{2}\overline{\omega^{2}})\hat{\Omega} + s^{2}\qty((1/2)\overline{\omega^{2}} +(2/9)(\delta\,s)^{2}\overline{\omega^{4}})) \ket{\psi} \nonumber\\ &amp; \quad = 2(\delta\,s)^{2}\bra{\psi} \qty(\hat{\Omega} + (1/4)s^{2}\overline{\omega^{2}}) \ket{\psi} + O((\delta\,s)^{4})\end{align*}

Next we specialize to the case of standard pulsed homodyne to obtain tighter bounds. For standard pulsed homodyne, $\omega_{k} = \omega$ for all k, and with our conventions, $\omega = \omega_{1} = 1$. This gives $\phi_{k} = \phi = [\delta\,s]_{\pi}$ and $h_{k}(\delta\,s) = h(\delta\,s) = \phi/(\delta\,s)-(\phi/2)\cot(\phi/2)$ for all k. As noted when we defined $\hat{u}_{\delta\,s}$, we now have $\hat{u}_{\delta\,s} = \hat{q}$. According to the definitions, $u(\delta\,s)^{2} = h(\delta\,s)$. From equation (C32), $\phi^{2}{\unicode{x2A7D}} (\delta\,s)^{2}$ and $|h(\delta\,s)|{\unicode{x2A7D}} (\delta\,s)^{2}/3$. Substituting in the bound of equation (C25) with θ=0 gives

\begin{align*} \left|\mathrm e^{-\mathrm i\hat q_{\delta}s}\ket{\psi} - \mathrm e^{-\mathrm i\hat{q}s}\ket{\psi}\right|^{2} {\unicode{x2A7D}} (\delta\,s)^{2}\qty(2\bra{\psi}\hat{n}_{\mathrm{tot}}\ket{\psi} +s^{2}\qty(\frac{1}{9}(\delta\,s)^{2}\bra{\psi}\hat{q}^{2}\ket{\psi}+\frac{1}{2})).\end{align*}

For comparison in the regime of small $\delta\,s$ and s, we determine lower bounds on the minimum over angles θ of $|\mathrm e^{-\mathrm i\hat{q}_{\delta}\hat{s}_{M}}\ket{\psi} - \mathrm e^{-\mathrm i\theta}\mathrm e^{-\mathrm i\hat{q}\hat{s}_{M}}\ket{\psi}|^{2}$, where $\ket{\psi}$ is in a family of coherent states. We begin by considering a generic mode with the operators defined in equation (C4), where we omit the mode index k. Without loss of generality, we can assume that α and β are non-negative real. For $\vec{\gamma} = (\gamma_{a},\gamma_{b})^\mathrm {T}$ with $\gamma_{a}$ and $\gamma_{b}$ real, let $\ket{\vec{\gamma}} = \ket{\mathrm i\gamma_{a},\gamma_{b}}$ be the state with amplitudes $i\gamma_{a}$ and $\gamma_{b}$ on modes a and b. We have

\begin{align*} \mathrm e^{-\mathrm i\vec{u}^\mathrm {T}\vec{q}}\ketbra{\gamma}{\gamma}\mathrm e^{\mathrm i\vec{u}^\mathrm {T}\vec{q}} &amp; = \ketbra{\vec{\gamma}-\beta \vec{u}}{\vec{\gamma}-\beta \vec{u}}, \nonumber\\ \mathrm e^{-\mathrm i\phi \hat{B}}\ketbra{\vec{\gamma}}{\vec{\gamma}}\mathrm e^{\mathrm i\phi \hat{B}} &amp; = \ketbra{R_{\phi}\vec{\gamma}}{R_{\phi}\vec{\gamma}}.\end{align*}

We consider initial states of the form $\ket{\vec{\gamma}_{0}} = \ket{i\gamma_{a},0}$ with $\vec{\gamma}_{0} = (\gamma_{a},0)^\mathrm {T}$ that are vacuum on the LO mode. We apply equation (C11) to determine

\begin{align*} \mathrm e^{\mathrm it( \vec{w}^\mathrm {T}\vec{q}+\delta\hat{B})}\mathrm e^{-\mathrm it \vec{w}^\mathrm {T}\vec{q}} \ket{\vec{\gamma}_{0}} = \mathrm e^{\mathrm i\varphi} \ket{R_{-\phi}(\vec{\gamma}_{0}-\beta t \vec{w}) + (\beta/\delta) (1-R_{-\phi}) S \vec{w}}\end{align*}

for some angle φ, where $\phi = [t\delta]_{\pi}$ as defined after equation (C11). Substituting $\vec{w} = (1,0)^\mathrm {T}$ gives

\begin{align*} \mathrm e^{\mathrm it( \hat{q}_{a}+\delta\hat{B})}\mathrm e^{-\mathrm it \hat{q}_{a}}\ket{\gamma_{0}} &amp; = \mathrm e^{\mathrm i\varphi} \big|i(\cos(\phi)(\gamma_{a}-\beta t) + (\beta/\delta)\sin(\phi)), \nonumber\\ &amp;\phantom{ = \mathrm e^{\mathrm i\varphi}\big|\;} -\sin(\phi)(\gamma_{a}-\beta t) -(\beta/\delta)(1-\cos(\phi))\big\rangle.\end{align*}

Accordingly, $\left|\bra{\gamma_{0}}\mathrm e^{\mathrm it( \hat{q}_{a}+\delta\hat{B})}\mathrm e^{-\mathrm it \hat{q}_{a}}\ket{\gamma_{0}}\right| = \mathrm e^{-|\Delta(\gamma_{a})|^{2}/2}$ with

\begin{align*} \Delta(\gamma_{a}) = \begin{pmatrix} (\cos(\phi)-1)\gamma_{a}+(\sin(\phi)/(\delta t)-\cos(\phi))\beta t\\ -\sin(\phi)(\gamma_{a}-\beta t) -(\beta/\delta)(1-\cos(\phi)) \end{pmatrix}.\end{align*}

We now reintroduce the mode index. Let $\gamma_{k}$ be the coherent amplitude $\gamma_{a_{k}}$ and $\Delta_{k}(\gamma_{k})$ the corresponding amplitude difference vector. Let $\ket{\psi}$ be the coherent state whose amplitudes on mode $a_{k}$ are $i\gamma_{a_{k}}$ and vacuum on the LO modes. Multiplying over the modes gives

\begin{align*} \left|\bra{\psi} \mathrm e^{\mathrm i\hat{q}_{\delta}s}\mathrm e^{-{\mathrm i}\hat{q}s}\ket{\psi}\right| = \mathrm e^{-\sum_{k}|\Delta_{k}(\gamma_{k})|^{2}/2},\end{align*}

where

\begin{align*} \Delta_{k}(\gamma_{k}) = \begin{pmatrix} (\cos(\phi_{k})-1)\gamma_{k}+(\sin(\phi_{k})/(\omega_{k}\delta\,s)-\cos(\phi)_{k})\alpha_{k}s\\ -\sin(\phi_{k})(\gamma_{k}-\alpha_{k} s) -(\alpha_{k}s/(\omega_{k} \delta\,s))(1-\cos(\phi_{k})) \end{pmatrix}.\end{align*}

To compare with the bound of equation (C37), we consider small $\delta\,s$ and s, and choose $\gamma_{k}$ to witness a lower bound approaching that of equation (C37). Consider the case where for all k, $\omega_{k}\delta\,s \in [-\pi/4,\pi/4]$, so that $\phi_{k} = \omega_{k}\delta\,s$. The second coordinate of $\Delta_{k}(\gamma_{k})$ has contributions of order $\delta\,s$ and $\delta\,s^{2}$ and dominate the contributions of order $(\delta\,s)^{2}$ of the first coordinate. We neglect the first coordinate for the bound

\begin{align*} |\Delta_{k}(\gamma_{k})|^{2} &amp;{\unicode{x2A7E}} \qty(\sin(\phi_{k})(\gamma_{k}-\alpha_{k} s) +(\alpha_{k}s/\phi_{k})(1-\cos(\phi_{k})))^{2} \nonumber\\ &amp; = \qty(\sin(\phi_{k})\gamma_{k} +\alpha_{k}s((1-\cos(\phi_{k}))/\phi_{k}-\sin(\phi_{k})))^{2}.\end{align*}

The expression on the right-hand side that is being squared is odd in $\phi_{k}$. For $0{\unicode{x2A7D}} x{\unicode{x2A7D}} \pi/4$, we have

\begin{align*} \sin(x)&amp;{\unicode{x2A7E}} (4/(\sqrt{2}\pi))x{\unicode{x2A7E}} (9/10)x, \nonumber\\ (1-\cos(x)) &amp;{\unicode{x2A7D}} x^{2}/2 \nonumber\\ \sin(x) - (1-\cos(x))/x &amp; {\unicode{x2A7E}} (4/(\sqrt{2}\pi) - 1/2)x {\unicode{x2A7E}} (2/5)x.\end{align*}

We choose $\gamma_{k}$ to have the opposite sign of s, so that $\sin(\phi_{k})\gamma_{k}$ and $\alpha_{k}s((1-\cos(\phi_{k}))/\phi_{k}-\sin(\phi_{k}))$ have the same sign. Then, omitting the cross term when squaring,

\begin{align*} |\Delta_{k}(\gamma_{k})|^{2} &amp;{\unicode{x2A7E}} (9/10)^{2}\phi_{k}^{2}|\gamma_{k}|^{2} + (2/5)^{2}\phi_{k}^{2}|\alpha_{k} s|^{2} \nonumber\\ &amp; = (9/10)^{2}(\delta\,s)^{2} \qty(\omega_{k}^{2}|\gamma_{k}|^{2} + (4/9)^{2} \omega_{k}^{2}|\alpha_{k}|^{2}s^{2}).\end{align*}

We sum this over k for

\begin{align*} \sum_{k} |\Delta_{k}(\gamma_{k})|^{2} {\unicode{x2A7E}} (9/10)^{2}(\delta\,s)^{2}\bra{\vec{\gamma}_{0}} \hat{\Omega} + (4/9)^{2} s^{2}\overline{\omega^{2}} \ket{\vec{\gamma}_{0}}.\end{align*}

By applying $\mathrm e^{-x^{2}/2} = 1-x^{2}/2+O(x^{4})$, we can now estimate

\begin{align*} \min_{\theta}|\mathrm e^{-\mathrm i\hat{q}_{\delta}\hat{s}_{M}}\ket{\psi} - \mathrm e^{-\mathrm i\theta}\mathrm e^{-\mathrm i\hat{q}\hat{s}_{M}}\ket{\psi}|^{2} &amp; = 2\qty(1- {e}^{-\sum_{k}|\Delta_{k}(\gamma_{k})|^{2}/2}) \nonumber\\ &amp; = (9/10)^{2}(\delta\,s)^{2}\bra{\vec{\gamma}_{0}} \hat{\Omega} + (4/9)^{2} s^{2}\overline{\omega^{2}} \ket{\vec{\gamma}_{0}} + O((\delta\,s)^{4}).\end{align*}

This may be compared to equation (C37), which gives an upper bound of the same form up to order $(\delta\,s)^{4}$ with somewhat larger constants.

## Appendix D Bounds for standard pulsed homodyne

We establish the results of section 5 for standard pulsed homodyne to obtain better bounds. See section 7 for comparisons. The proofs parallel those of section 5, and we point out the differences as needed. Throughout this section, we set $\omega_{k} = \omega = 1$. The total photon number operator for modes $\boldsymbol{a}$ is $\hat{n}_{\mathrm{tot}}$.
Theorem D.1 (Theorem 5.1 for standard pulsed homodyne). Let $\ket{\psi}$ be a joint state of the signal, LO modes, the apparatus M and any other relevant systems including those needed for purifying the state. Assume that these states are vacuum on the LO modes. Then

\begin{align*} \left|\mathrm e^{-\mathrm i\hat q_{\delta}s}\ket{\psi} - \mathrm e^{-\mathrm i\hat{q}s}\ket{\psi}\right|^{2} {\unicode{x2A7D}} (\delta\,s)^{2}\qty(2\bra{\psi_{s}}\hat{n}_{\mathrm{tot}}\ket{\psi_{s}} +s^{2}\qty(\frac{1}{9}(\delta s)^{2}\bra{\psi_{s}}\hat{q}^{2}\ket{\psi_{s}}+\frac{1}{2})).\end{align*}

For every self-adjoint apparatus operator $\hat{s}_{M}$,

\begin{align*} \left|\mathrm e^{-\mathrm i\hat q_{\delta}\hat{s}_{M}}\ket{\psi} - \mathrm e^{-\mathrm i\hat{q}\hat{s}_{M}}\ket{\psi}\right|^{2} {\unicode{x2A7D}} \delta^{2}\bra{\psi_{s}}\hat{s}_{M}^{2}\qty(2\hat{n}_{\mathrm{tot}} +\hat{s}_{M}^{2}\qty(\frac{1}{9}\delta^{2}\hat{s}_{M}^{2}\hat{q}^{2}+\frac{1}{2}))\ket{\psi_{s}}.\end{align*}

Proof. The inequality of equation (D1) is identical to that of equation (C38). The proof of equation (D2) parallels the proof of the corresponding bound in theorem 5.1, which justifies substituting the operator $\hat{s}_{M}$ for $\hat{s}$ in equation (D1). □
Theorem D.2 (Theorem 5.2 for standard pulsed homodyne). Let $\hat{s}_{M}$ have non-degenerate spectrum so that the apparatus Hilbert space has a representation $L^{2}(\mathbb{R}, \mathrm d\mu(s))$, and let $\ket{g(\boldsymbol{\cdot})}$ be the initial state of the apparatus. Define

\begin{align*} b_{g,2l} = \int_{s\in\mathbb{R}} \mathrm d\mu(s) s^{2l}|g(s)|^{2}.\end{align*}

Let $\ket{\psi}$ the initial state of the signal, LO modes and systems other than the apparatus, where $\ket{\psi}$ is vacuum on the LO modes. Then, with $\ket{\phi_{\delta}}$ as in equation (10), we have

\begin{align*} \left|\ket{\phi_{\delta}}-\ket{\phi_{0}}\right|^{2} {\unicode{x2A7D}} \delta^{2}\qty( {2} b_{g,2}\bra{\psi}\hat{n}_{\mathrm{tot}}\ket{\psi} + \frac{1}{9}\delta^{2}b_{ g,6}\bra{\psi}\hat{q}^{2}\ket{\psi}+ \frac{1}{2}b_{ g,4} ).\end{align*}

Proof. The proof parallels that of theorem 5.2. □
Proposition D.3 (Proposition 5.3 for standard pulsed homodyne). Suppose that f(x) is the Fourier transform of a bounded complex measure $\mathrm d\tilde\mu(\kappa)$. Write $\mathrm d\tilde\mu(\kappa) = \mathrm e^{\mathrm i\theta(\kappa)}\mathrm d\mu(\kappa)$ for a bounded positive measure $\mathrm d\mu(\kappa)$. Define $\tilde{f}_{l} = \int_{\kappa\in\mathbb{R}} \mathrm d\mu(\kappa) |\kappa|^{l}$. Then for all states $\ket{\psi}$ of the signal and LO modes and other relevant systems, where $\ket{\psi}$ is vacuum on the LO modes, we have the following bound:

\begin{align*} \left|f(\hat{q}_{\delta})\ket{\psi}-f(\hat{q}_{0})\ket{\psi}\right|^{2} {\unicode{x2A7D}} \delta^{2}(\tilde f_{0}+\tilde f_{2})\qty( 2\bra{\psi}\hat{n}_{\mathrm{tot}}\ket{\psi} + \frac{1}{9}\delta^{2}\tilde f_{4}\bra{\psi}\hat{q}^{2}\ket{\psi} + \frac{1}{2}\tilde f_{2}).\end{align*}

Proof. The proof of proposition 5.3 needs to be modified starting at the third to last line of equation (26), where we now apply equation (D1) with s replaced by −κ:

\begin{align*}&amp;\left|\bra{\phi}f(\hat{q}_{\delta}) - f(\hat{q}_{0})\ket{\psi}\right|^{2} \kern-1in \nonumber\\ &amp;\quad {\unicode{x2A7D}} \int_{\kappa\in\mathbb{R}}\mathrm d\mu(\kappa)(1+\kappa^{2}) \int_{\kappa\in\mathbb{R}}\mathrm d\mu(\kappa)\frac{1}{1+\kappa^{2}} \left|\qty(\mathrm e^{\mathrm i\kappa\hat{q}_{\delta}}- \mathrm e^{\mathrm i\kappa\hat{q}_{0}})\ket{\psi}\right|^{2} \nonumber\\ &amp;\quad {\unicode{x2A7D}} \delta^{2}(\tilde f_{0}+\tilde f_{2}) \int_{\kappa\in\mathbb{R}}\mathrm d\mu(\kappa)\frac{1}{1+\kappa^{2}}\kappa^{2} \qty(2 \bra{\psi}\hat{n}_{\mathrm{tot}}\ket{\psi} + \kappa^{2}\qty(\frac{1}{9}\delta^{2}\kappa^{2}\bra{\psi}\hat{q}^{2}\ket{\psi} + \frac{1}{2}) ) \nonumber\\ &amp;\quad {\unicode{x2A7D}} \delta^{2}(\tilde f_{0}+\tilde f_{2})\qty( 2\bra{\psi}\hat{n}_{\mathrm{tot}}\ket{\psi} + \frac{1}{9}\delta^{2}\tilde f_{4}\bra{\psi}\hat{q}^{2}\ket{\psi} + \frac{1}{2}\tilde f_{2}).\end{align*}

The result then follows as before. □

## Appendix E A generalization of proposition 5.3

In general, defining functions of two non-commuting self-adjoint operators is problematic and requires operator ordering conventions and care with the operators’ projection valued measures. A systematic approach can be based on multiple operator integrals as explained in [29]. Because for δ>0 $\hat{q}_{\delta}$ has discrete spectrum, for bounded measurable functions f(x,y) we can directly define

\begin{align*} f(\hat{q}_{\delta},\hat{q})\ket{\psi} = \int_{x\in\mathbb{R},y\in\mathbb{R}} f(x,y)\mathrm d\Pi_{\delta}(x)\mathrm d\Pi(y)\ket{\psi} = \sum_{l}\Pi_{\delta,l}\int_{y\in\mathbb{R}}f(x_{l},y)\mathrm d\Pi(y)\ket{\psi},\end{align*}

where $\{x_{l}\}_{l}$ enumerates the distinct values in the spectrum of $\hat{q}_{\delta}$, and $\Pi_{\delta,l}$ are the corresponding eigenspace projectors. Because f(x,y) is bounded, we have that $f(\hat{q}_{\delta},\hat{q})$ is a bounded operator.

The following proposition generalizes proposition 5.3 with a better bound, and its proof expands on that of the latter proposition. We consider multiple BBP homodyne measurements in parallel. For this purpose we vectorize the measurement operators. We consider m independent pairs of signal and LO modes and let $\boldsymbol{\hat{q}}_{\delta}$ be the vector whose j‘th entry consists of the j‘th pair’s operator $\hat{q}_{\delta}$, which we write as $\hat{q}_{j,\delta}$. We let $\mathrm d\Pi_{\delta}(\boldsymbol{x})$ be the joint projection valued measure for $\boldsymbol{\hat{q}}_{\delta}$. Let $\hat{\Omega}_{j}$ be the operator $\hat{\Omega}$ defined in theorem 5.1 for the j‘th pair, and $\overline{\omega_{j}^{2}}$ the quantity $\overline{\omega^{2}}$ for the j‘th pair.
Proposition E.1. Suppose that $f(\boldsymbol{x},\boldsymbol{y})$ is a function on $\mathbb{R}^{m}\times\mathbb{R}^{m}$ that is the Fourier transform of a bounded complex measure $\mathrm d\tilde\mu(\boldsymbol{\kappa},\boldsymbol{\kappa}^{^{\prime}})$ and satisfies $f(\boldsymbol{x},\boldsymbol{x}) = 0$. Write $\mathrm d\tilde\mu(\boldsymbol{\kappa},\boldsymbol{\kappa}^{^{\prime}}) = \mathrm e^{\mathrm i\theta(\boldsymbol{\kappa},\boldsymbol{\kappa}^{^{\prime}})}\mathrm d\mu(\boldsymbol{\kappa},\boldsymbol{\kappa}^{^{\prime}})$ with $\mathrm d\mu(\boldsymbol{\kappa},\boldsymbol{\kappa}^{^{\prime}})$ positive. Define the marginal distributions $\mathrm d\mu(\boldsymbol{\kappa}) = \int_{\boldsymbol{\kappa}^{^{\prime}}\in\mathbb{R}^{m}}\mathrm d\mu(\boldsymbol{\kappa},\boldsymbol{\kappa}^{^{\prime}})$ and $\mathrm d\mu(\kappa_{j}) = \int_{\boldsymbol{\kappa}\in\mathbb{R}^{m},\boldsymbol{\kappa}^{^{\prime}}\in\mathbb{R}^{m}:(\boldsymbol{\kappa})_{j} = \kappa_{j}}$ $ \mathrm d\mu(\boldsymbol{\kappa},\boldsymbol{\kappa}^{^{\prime}})$. Let $\tilde{f}_{j,l} = \int_{\kappa_{j}\in\mathbb{R}} \mathrm d\mu(\kappa_{j}) |\kappa_{j}|^{l}$, $\tilde{f}_{\mathrm{tot},l} = \sqrt{\sum_{j = 1}^{m}\tilde{f}_{j,l}^{2}}$, $\hat{\Omega}_{\mathrm{tot}} = \sum_{j = 1}^{m}\hat{\Omega}_{j}$, and $\overline{\omega^{2}_{\mathrm{tot}}} = \sum_{j = 1}^{m}\overline{\omega^{2}_{j}}$. Then for all states $\ket{\psi}$ of the signal and LO modes and other relevant system that are vacuum on the LO modes

\begin{align*} |f(\hat{\boldsymbol{q}}_{\delta},\hat{\boldsymbol{q}})\ket{\psi}|^{2} {\unicode{x2A7D}} 4\delta^{2} \bra{\psi}\qty( \qty(\tilde f_{\mathrm{tot},1}^{2}+\tilde f_{\mathrm{tot},2}^{2})\hat{\Omega}_{\mathrm{tot}} +\tilde f_{\mathrm{tot},2}^{2}\overline{\omega^{2}_{\mathrm{tot}}})\ket{\psi} .\end{align*}

Before we prove the proposition, to see that this bound improves on that in proposition 5.3, apply the Cauchy–Schwarz inequality twice for

\begin{align*}\tilde f_{\mathrm{tot},1}^{2} &amp; = \sum_{j = 1}^{m}\qty(\int_{\kappa_{j}\in\mathbb{R}}|\kappa_{j}|\mathrm d\mu(\kappa_{j}))^{2} \nonumber\\ &amp;{\unicode{x2A7D}} \sum_{j = 1}^{m}\int_{\kappa_{j}\in\mathbb{R}}\mathrm d\mu(\kappa_{j}) \int_{\kappa_{j}\in\mathbb{R}}|\kappa_{j}|^{2}\mathrm d\mu(\kappa_{j}) \nonumber\\ &amp; = \sum_{j = 1}^{m}\tilde f_{j,0}\tilde f_{j,2} \nonumber\\ &amp;{\unicode{x2A7D}} \sqrt{\sum_{j = 1}^{m}\tilde f_{j,0}^{2}}\sqrt{\sum_{j = 1}^{m}\tilde f_{j,2}^{2}} \nonumber\\ &amp; = \tilde f_{\mathrm{tot},0}\tilde f_{\mathrm{tot},2}.\end{align*}

Therefore, the coefficient of $\hat{\Omega}_{\mathrm{tot}}$ is bounded by $(\tilde f_{\mathrm{tot},0}+\tilde f_{\mathrm{tot},2})\tilde f_{\mathrm{tot},2}$, and so is the coefficient of $\overline{\omega^2_\textrm{tot}}$.
Proof. The quantity $ |f(\hat{\boldsymbol{q}}_{\delta},\hat{\boldsymbol{q}})\ket{\psi}|$ can be expressed as

\begin{align*} |f(\hat{\boldsymbol{q}}_{\delta},\hat{\boldsymbol{q}})\ket{\psi}| = \max_{\ket{\phi}}\Re(\bra{\phi}f(\hat{\boldsymbol{q}}_{\delta},\hat{\boldsymbol{q}})\ket{\psi}).\end{align*}

In computing this maximum it suffices to consider $\ket{\phi}$ such that $\bra{\phi}f(\hat{\boldsymbol{q}}_{\delta},\hat{\boldsymbol{q}})\ket{\psi}$ is real. Consider an arbitrary such $\ket{\phi}$. We have

\begin{align*} \bra{\phi}f(\hat{\boldsymbol{q}}_{\delta}, \hat{\boldsymbol{q}})\ket{\psi} &amp; = \int_{\boldsymbol{x}\in\mathbb{R}^{m},\boldsymbol{y}\in\mathbb{R}^{m}}f(\boldsymbol{x},\boldsymbol{y})\bra{\phi}\mathrm d\Pi_{\delta}(\boldsymbol{x})\mathrm d\Pi(\boldsymbol{y})\ket{\psi}, \nonumber\\ &amp; = \int_{\boldsymbol{x}\in\mathbb{R}^{m},\boldsymbol{y}\in\mathbb{R}^{m}}\qty(\int_{\boldsymbol{\kappa}\in\mathbb{R}^{m},\boldsymbol{\kappa}^{^{\prime}}\in\mathbb{R}^{m}}\mathrm d\tilde\mu(\boldsymbol{\kappa},\boldsymbol{\kappa}^{^{\prime}}) \mathrm e^{\mathrm i(\boldsymbol{\kappa}\cdot \boldsymbol{x}+\boldsymbol{\kappa}^{^{\prime}}\cdot\boldsymbol{y})}) \bra{\phi}\mathrm d\Pi_{\delta}(\boldsymbol{x})\mathrm d\Pi(\boldsymbol{y})\ket{\psi}.\end{align*}

The integrand in this integral is bounded and in view of the comments at the beginning of the proof of proposition 5.3 and taking advantage of the discrete spectrum of $\hat{\boldsymbol{q}}_{\delta}$ for δ>0, the expression $d\tilde \mu(\boldsymbol{\kappa},\boldsymbol{\kappa}^{^{\prime}}) \bra{\phi}\mathrm d\Pi_{\delta}(\boldsymbol{x})\mathrm d\Pi(\boldsymbol{y})\ket{\psi}$ defines a bounded complex measure on $\mathbb{R}^{m}\times \mathbb{R}^{m}$. We can therefore apply the Fubini-Tonelli theorem to change order of integration. We obtain

\begin{align*} \bra{\phi}f(\hat{\boldsymbol{q}}_{\delta}, \hat{\boldsymbol{q}})\ket{\psi} = \int_{\boldsymbol{\kappa}\in\mathbb{R}^{m},\boldsymbol{\kappa}^{^{\prime}}\in\mathbb{R}^{m}}\mathrm d\tilde\mu(\boldsymbol{\kappa},\boldsymbol{\kappa}^{^{\prime}}) \qty(\int_{\boldsymbol{x}\in\mathbb{R}^{m},\boldsymbol{y}\in\mathbb{R}^{m}} \mathrm e^{\mathrm i(\boldsymbol{\kappa}\cdot \boldsymbol{x}+\boldsymbol{\kappa}^{^{\prime}}\cdot\boldsymbol{y})} \bra{\phi}\mathrm d\Pi_{\delta}(\boldsymbol{x})\mathrm d\Pi(\boldsymbol{y})\ket{\psi}).\end{align*}

The inner integral is

\begin{align*} \int_{\boldsymbol{x}\in\mathbb{R}^{m},\boldsymbol{y}\in\mathbb{R}^{m}} \mathrm e^{\mathrm i(\boldsymbol{\kappa}\cdot \boldsymbol{x}+\boldsymbol{\kappa}^{^{\prime}}\cdot \boldsymbol{y})} \bra{\phi}\mathrm d\Pi_{\delta}(\boldsymbol{x})\mathrm d\Pi(\boldsymbol{y})\ket{\psi} \kern-1in &amp;\nonumber\\ &amp; = \bra{\phi} \mathrm e^{\mathrm i\boldsymbol{\kappa}\cdot \hat{\boldsymbol{q}}_{\delta}} \mathrm e^{\mathrm i\boldsymbol{\kappa}^{^{\prime}}\cdot \hat{\boldsymbol{q}}}\ket{\psi} \nonumber\\ &amp; = \bra{\phi}\mathrm e^{\mathrm i(\boldsymbol{\kappa}+\boldsymbol{\kappa}^{^{\prime}})\cdot \hat{\boldsymbol{q}}}\ket{\psi} + \bra{\phi}\qty(\mathrm e^{\mathrm i\boldsymbol{\kappa}\cdot \hat{\boldsymbol{q}}_{\delta}} -\mathrm e^{\mathrm i\boldsymbol{\kappa}\cdot \hat{\boldsymbol{q}}}) \mathrm e^{\mathrm i\boldsymbol{\kappa}^{^{\prime}}\cdot \hat{\boldsymbol{q}}}\ket{\psi}.\end{align*}

To continue, we chain the triangle inequality as follows: Define $\hat{\boldsymbol{q}}_{\delta|j}$ so that the $j^{^{\prime}}$‘th entry of $\hat{\boldsymbol{q}}_{\delta|j}$ is $\hat{q}_{j^{^{\prime}},0}$ for $j^{^{\prime}}{\unicode{x2A7D}} j$ and $\hat{q}_{j^{^{\prime}},\delta}$ otherwise. Then $\hat{\boldsymbol{q}}_{\delta|0} = \hat{\boldsymbol{q}}_{\delta}$ and $\hat{\boldsymbol{q}}_{\delta|m} = \hat{\boldsymbol{q}}$. Consequently

\begin{align*} \mathrm e^{\mathrm i\boldsymbol{\kappa}\cdot\hat{q}_{\delta}}-\mathrm e^{\mathrm i\boldsymbol{\kappa}\cdot\hat{q}} &amp; = \sum_{j = 0}^{m-1} \mathrm e^{\mathrm i\boldsymbol{\kappa}\cdot\hat{\boldsymbol{q}}_{\delta|j}} -\mathrm e^{\mathrm i\boldsymbol{\kappa}\cdot\hat{\boldsymbol{q}}_{\delta|j+1}} \nonumber\\ &amp; = \sum_{j = 0}^{m-1} \qty(\mathrm e^{\mathrm i\kappa_{j+1}\hat{q}_{j+1,\delta}}-\mathrm e^{\mathrm i\kappa_{j+1}\hat{q}_{j+1,0}}) \mathrm e^{\mathrm i\qty(\boldsymbol{\kappa}\cdot\hat{\boldsymbol{q}}_{\delta|j+1}-\kappa_{j+1}\hat{q}_{j+1,0})}.\end{align*}

After substituting into the last summand of equation (E7) we get a sum of terms of the form

\begin{align*} \bra{\phi} \qty(\mathrm e^{\mathrm i\kappa_{j+1}\hat{q}_{\delta|j}}-\mathrm e^{\mathrm i\kappa_{j+1}\hat{q}_{j}})\hat{V}_{j+1} \ket{\psi},\end{align*}

where $\hat{V}_{j}$ is unitary and does not affect the vacuum state of the LO modes of the j‘th pair of signal and LO modes. Each term can therefore be bounded by applying equation (17) for

\begin{align*} \left|\bra{\phi} \qty(\mathrm e^{\mathrm i\kappa_{j+1}\hat{q}_{\delta,j}}-\mathrm e^{\mathrm i\kappa_{j+1}\hat{q}_{j}})\hat{V}_{j} \ket{\psi}\right| {\unicode{x2A7D}} 2\delta|\kappa_{j+1}|\sqrt{\bra{\psi} \qty(\hat{\Omega}_{j+1} + \kappa_{j+1}^{2}\qty(\hat{\Omega}_{j+1}+\overline{\omega_{j+1}^{2}})) \ket{\psi}},\end{align*}

with $j\in\{0,\ldots, m-1\}$. Continuing from equation (E7) we obtain

\begin{align*}\int_{\boldsymbol{x}\in\mathbb{R}^{m},\boldsymbol{y}\in\mathbb{R}^{m}} \mathrm e^{\mathrm i(\boldsymbol{\kappa}\cdot \boldsymbol{x}+\boldsymbol{\kappa}^{^{\prime}}\cdot \boldsymbol{y})} \bra{\phi}\mathrm d\Pi_{\delta}(\boldsymbol{x})\mathrm d\Pi(\boldsymbol{y})\ket{\psi} \kern-1in &amp;\nonumber\\ &amp;\in \bra{\phi}\mathrm e^{\mathrm i(\boldsymbol{\kappa}+\boldsymbol{\kappa}^{^{\prime}})\cdot \hat{\boldsymbol{q}}}\ket{\psi} + \boldsymbol{B}_{1} 2\delta\sum_{j = 1}^{m} |\kappa_{j}|\sqrt{\bra{\psi} \qty(\hat{\Omega}_{j} + \kappa_{j}^{2}\qty(\hat{\Omega}_{j}+\overline{\omega_{j}^{2}})) \ket{\psi}},\end{align*}

with $\boldsymbol{B}_{1}$ the unit disk in the complex plain. Substituting the right-hand side into equation (E6) and applying the bounds gives

\begin{align*} \bra{\phi}f(\hat{\boldsymbol{q}}_{\delta}, \hat{\boldsymbol{q}})\ket{\psi} &amp;{\unicode{x2A7D}} \left|\int_{\boldsymbol{\kappa}\in\mathbb{R}^{m},\boldsymbol{\kappa}^{^{\prime}}\in\mathbb{R}^{m}}\mathrm d\tilde\mu(\boldsymbol{\kappa},\boldsymbol{\kappa}^{^{\prime}}) \bra{\phi}\mathrm e^{\mathrm i(\boldsymbol{\kappa}+\boldsymbol{\kappa}^{^{\prime}})\hat{\boldsymbol{q}}}\ket{\psi}\right| \nonumber\\ &amp;\phantom{{}{\unicode{x2A7D}} \int} {}+2\delta\sum_{j = 1}^{m}\int_{\boldsymbol{\kappa}\in\mathbb{R}^{m},\boldsymbol{\kappa}^{^{\prime}}\in\mathbb{R}^{m}}\mathrm d\mu(\boldsymbol{\kappa},\boldsymbol{\kappa}^{^{\prime}}) |\kappa_{j}|\sqrt{\bra{\psi} \qty(\hat{\Omega}_{j} + \kappa_{j}^{2}\qty(\hat{\Omega}_{j}+\overline{\omega_{j}^{2}})) \ket{\psi}}.\end{align*}

The integral under the absolute value evaluates to

\begin{align*} \int_{\boldsymbol{\kappa}\in\mathbb{R}^{m},\boldsymbol{\kappa}^{^{\prime}}\in\mathbb{R}^{m}}\mathrm d\tilde\mu(\boldsymbol{\kappa},\boldsymbol{\kappa}^{^{\prime}}) \bra{\phi}\mathrm e^{\mathrm i(\boldsymbol{\kappa}+\boldsymbol{\kappa}^{^{\prime}})\hat{\boldsymbol{q}}}\ket{\psi} &amp; = \int_{\boldsymbol{x}\in\mathbb{R}^{m}}\int_{\boldsymbol{\kappa}\in\mathbb{R}^{m},\boldsymbol{\kappa}^{^{\prime}}\in\mathbb{R}^{m}} \mathrm d\tilde\mu(\boldsymbol{\kappa},\boldsymbol{\kappa}^{^{\prime}})\mathrm e^{\mathrm i(\boldsymbol{\kappa}+\boldsymbol{\kappa}^{^{\prime}})\cdot\boldsymbol{x}} \bra{\phi}\mathrm d\Pi(\boldsymbol{x})\ket{\psi} \nonumber\\ &amp; = \int_{\boldsymbol{x}\in\mathbb{R}^{m}} f(\boldsymbol{x},\boldsymbol{x}) \bra{\phi}\mathrm d\Pi(\boldsymbol{x})\ket{\psi} \nonumber\\ &amp; = 0.\end{align*}

For the second integral we get

\begin{align*} 2\delta\sum_{j = 1}^{m} \int_{\boldsymbol{\kappa}\in\mathbb{R}^{m},\boldsymbol{\kappa}^{^{\prime}}\in\mathbb{R}^{m}}\mathrm d\mu(\boldsymbol{\kappa},\boldsymbol{\kappa}^{^{\prime}}) |\kappa_{j}|\sqrt{\bra{\psi} \qty(\hat{\Omega}_{j} + \kappa_{j}^{2}\qty(\hat{\Omega}_{j}+\overline{\omega_{j}^{2}})) \ket{\psi}} \kern-2.5in &amp;\nonumber\\ &amp;{\unicode{x2A7D}} {2}\delta \sum_{j = 1}^{m}\int_{\kappa_{j}\in\mathbb{R}} \mathrm d\mu(\kappa_{j})\, |\kappa_{j}|\qty(\sqrt{ \bra{\psi}\hat{\Omega}_{j}\ket{\psi}} + |\kappa_{j}| \sqrt{\bra{\psi}\hat{\Omega}_{j}\ket{\psi}+\overline{\omega_{j}^{2}}}) \nonumber\\ &amp; = {2}\delta\sum_{j = 1}^{m} \qty(\tilde f_{j,1}\sqrt{\bra{\psi}\hat{\Omega}_{j}\ket{\psi}}+ \tilde f_{j,2} \sqrt{\bra{\psi}\hat{\Omega}_{j}+\overline{\omega_{j}^{2}}\ket{\psi}}) \nonumber\\ &amp;{\unicode{x2A7D}} {2}\delta\qty( \tilde f_{\mathrm{tot},1}\sqrt{\bra{\psi}\hat{\Omega}_{\mathrm{tot}}\ket{\psi}} + \tilde f_{\mathrm{tot},2}\sqrt{\bra{\psi}\hat{\Omega}_{\mathrm{tot}} +\overline{\omega^{2}_{\mathrm{tot}}}\ket{\psi}} ),\end{align*}

where we used the inequality of equation (B2) for the second line and applied the the Cauchy–Schwarz inequality to each summand for the last line. The bound in the proposition follows by substitution and the inequality of equation (B3). □

## Appendix F Simulation of GKP fidelity

Figure F1 shows the performance of square GKP qubits with different average photon numbers. The infidelities for recovery maps after displacement noise applied to the GKP qubit is shown. Two recovery maps are considered. The first is a numerically optimized recovery map based on [32]. The second is recovery based on teleported error correction. For the second we use an analytic approximation from [33]. The numerical optimization was implemented using the Julia programming language [34]. The code used for these simulations was also used in [32] to obtain fidelities of rotation-symmetric codes against the simultaneous loss-dephasing channel.

The noise channel $\mathcal N(\hat\rho)$ we consider here is Gaussian distributed random displacement noise which can be obtained by solving the master equation

\begin{align*} \dot {\hat{\rho}} = \kappa\left(\mathcal D[\hat a] + \mathcal D[\hat a^\dagger]\right)(\hat\rho)\end{align*}

where $\mathcal D[\hat L] (\hat\rho) = \hat L \hat\rho \hat L^\dagger - \frac{1}{2} \hat L^\dagger \hat L \hat\rho - \frac{1}{2} \hat\rho \hat L^\dagger \hat L$. The solution to this master equation can be obtained by exponentiating the superoperator representation $\mathcal{L}[\hat{L}]$ of the Lindbladian $\mathcal{D}[\hat{L}]$. The superoperator representation is given by $\mathcal{L}[\hat{L}] = \hat L^* \otimes \hat L - \frac{1}{2} \hat I\otimes \hat L^\dagger \hat L - \frac{1}{2} (\hat L^\dagger \hat L)^T \otimes \hat I$. The noise channel evolved for time t is then given by

\begin{align*} \mathcal{N}_{\kappa t}(\hat{\rho}) = \left[\mathrm e^{\kappa t(\mathcal L[\hat{a}] + \mathcal L[\hat{a}^\dagger])}\right] (\hat{\rho})\;.\end{align*}

where $[\cdot](\hat{\rho})$ represents application of the given functional to $\hat{\rho}$. Thus κt represents the unitless strength of the random displacement rate. This noise strength can be related to the variance of the random displacement distribution given by the Kraus-operator representation of this channel

\begin{align*} \mathcal N_\sigma(\hat\rho) = \int \frac{\dd[2]{\alpha}}{\pi\sigma^2} \, \exp(-\frac{\abs{\alpha}^2}{\sigma^2}) \hat{D}_{\alpha}\, \hat{\rho}\, \hat{D}^\dagger_{\alpha}\,.\end{align*}

By writing equation (F2) and equation (F3) in terms of the characteristic function, we find that $\kappa t = \sigma^2$ which sets the x-coordinates in figure (F1). Note that for canonical quadratures, this channel corresponds to noise with variance $\sigma^2$ for both $\hat{x}$ and $\hat{p}$.

For the numerically optimized recovery operation, the total quantum channel we consider is

\begin{align*} \mathcal{L} = \mathcal R^{\mathrm{SDP}} \circ \mathcal N_{\sigma} \circ \mathcal E,\end{align*}

where $\mathcal{E}$ is the encoding isometry from a qubit into the GKP qubit space, $\mathcal N_{\sigma}$ is the noise map ( equation (F3)), $\mathcal R^{\mathrm{SDP}}$ is the numerically optimized recovery isometry from the GKP qubit space back to a qubit. The encoder is given by $\mathcal{E}[\hat\Pi](\hat\rho) = \hat\Pi \hat\rho {\hat\Pi}^\dagger$ where $\hat\Pi = \ket{0_L}\bra{0} + \ket{1_L}\bra{1}$ is the codespace projector while $\{\ket{i_L}\}_{i}$ is a basis for the logical subspace. We quantify the performance of the recovery map $\mathcal{R}^{\mathrm{SDP}}$ using the entanglement fidelity

\begin{align*} F_\mathrm e(\mathcal L) = \frac{1}{d^{2}}\sum_k \abs{\tr(\hat M_k)}^2\, ,\end{align*}

where $\{\hat M_k\}_{k}$ are the Kraus operators for $\mathcal{L}$ and d is the dimension of the channel’s input/output Hilbert space. For $\mathcal{L}$ given by equation (F4), d=2 and so the y-coordinate in figure F1 gives the entanglement infidelity $1 - F_\mathrm e(\mathcal{L})$ of the logical qubit channel.

We numerically implement the procedure in [35] which uses a semidefinite program (SDP) to compute the recovery $\mathcal R^\mathrm{SDP}$ that optimizes the channel fidelity in equation (F5). The finite energy GKP codewords are numerically represented in the Fock basis with a finite occupation cutoff using structures provided by the QuantumOptics.jl package [36]. They are created by superposing coherent states on a square grid in phase space with amplitudes exponentially damped by their distance from the origin. This procedure results in only approximately orthogonal code states $\bra{1_L}\ket{0_L} \neq 0$ and at large damping factors, this nonzero codeword overlap limits the achievable channel fidelity. To avoid this issue, we enforce orthogonality by removing the overlapping component from $\ket{1}_L$ and renormalizing. The resulting code states then always have zero mean $\bra{i_L}\hat{q}_{\theta}\ket{i_L} = 0$ for all quadratures $\hat{q}_{\theta}$.

We take advantage of the algorithm for sparse matrix exponentiation in [37, 38] to efficiently numerically compute equation (F2) and obtain the channel with noise strength κt. In the Fock basis representation, the tensor products used to form the superoperator representation $\mathcal{L}[\hat{L}]$ can be realized using the usual Kronecker matrix product and applied through right multiplication with the column-stacked vectorized $|\rho\rangle\rangle$. The SDP is then a function of $\mathcal{L}[\hat{L}]$ and $\hat{\Pi}$ with both numerically represented in the Fock basis. To solve this SDP, we use the COSMO.jl [39] convex optimizer via the JuMP.jl [40] interface, both of which are Julia-based packages. We set the relevant numerical tolerances for convergence of the solver to $10^{-8}$, well beyond the logical infidelities shown in figure F1. We furthermore ensure that the Fock cutoff is sufficiently large so that its effect on the calculations remains negligible. This cutoff is set so that there is at most $10^{-6}$ of infidelity between the codespace projectors calculated at the actually used cutoff versus the maximum cutoff (set to 400). This allows for dynamically scaling the cutoff based on the amount of GKP squeezing, which greatly reduces the simulation time and memory usage. We checked that this dynamic cutoff does not impact the optimal recovery infidelity by comparing the 14.9 dB square GKP with $\sigma_{\mathrm{noise}} = 0.1$ at the dynamically chosen cutoff of 220 to a maximum cutoff of 300, finding a fractional infidelity difference of less that 0.1%. The optimal recovery infidelity data along with the script generating figure F1 can be found in the Supplemental Material.

We also compute an analytic approximation of the channel fidelity for the linear optical recovery circuit shown in figure 4. This analytic approximation is based on section V.B.1. and appendix D of [33] along with appendix A of [41]. The approximation relies on displacement twirling, which allows approximation of a finitely squeezed GKP codeword as an incoherent mixture of infinite energy GKP codewords. The amount of squeezing is described using an envelop damping operator $\exp[-\Delta^2\hat{n}]$ applied to infinite energy GKP. Meanwhile the incoherent mixture of infinite energy GKP codewords is approximated as arising due to application of equation (F3) to an infinite energy GKP codeword $\mathcal{N}_{\sigma_{\mathrm{gkp}}}(\ket{s}\bra{s}_{\mathrm{gkp}})$. For these approximate GKP codewords, the probability of successful recovery using the closest lattice-point decoder for extracting the syndrome of one quadrature is given by

\begin{align*} p_{\mathrm{succ}}(\sigma_{\mathrm{eff}}) = \frac{1}{\sqrt{2\pi\sigma_{\mathrm{eff}}^2}}\sum_{n\in\mathbb{Z}} \int_{(2n-\frac{1}{2})\sqrt{\pi}}^{(2n+\frac{1}{2})\sqrt{\pi}}\dd{z}\, \mathrm e^{-\frac{z^2}{2\sigma_{\mathrm{eff}}^2}} \;.\end{align*}

For an isotropically squeezed GKP codespace, the entanglement fidelity of the logical channel is then given by $F_\mathrm e = [p_{\mathrm{succ}}(\sigma_{\mathrm{eff}})]^2$ where we set the effective noise variance according to $\sigma_{\mathrm{eff}}^2 = 3\sigma_{\mathrm{gkp}}^2 + \sigma_{\mathrm{noise}}^2$ for the analytic curves in figure F1. For the figure F1 legend, we give several alternative metrics besides $\sigma^2$ for quantifying the energy of a GKP code. The average photon number is given by $\bar{n} = \Tr(\hat{n}\Pi_L)$ while the amount of GKP squeezing in decibels is given by $-10\log_{10}(2\sigma^2)\,\mathrm{dB}$. The relationships between these parameters and the GKP envelope damping parameter Δ is given by $\bar{n} = 1/(2\Delta^2)$ and $\sigma_{\mathrm{gkp}}^2 = (1{-}\mathrm e^{-\Delta^2})/(1+\mathrm e^{-\Delta^2})$.
